# Genome-wide association analysis reveals a novel pathway mediated by a dual-TIR domain protein for pathogen resistance in cotton

**DOI:** 10.1186/s13059-023-02950-9

**Published:** 2023-05-10

**Authors:** Yihao Zhang, Yaning Zhang, Xiaoyang Ge, Yuan Yuan, Yuying Jin, Ye Wang, Lihong Zhao, Xiao Han, Wei Hu, Lan Yang, Chenxu Gao, Xi Wei, Fuguang Li, Zhaoen Yang

**Affiliations:** 1 Zhengzhou Research Base, National Key Laboratory of Cotton Bio-breeding and Integrated Utilization, Zhengzhou University, Zhengzhou, 450000 China; 2 National Key Laboratory of Cotton Bio-breeding and Integrated Utilization, Institute of Cotton Research, Chinese Academy of Agricultural Sciences, Anyang, 455000 China; 3 Western Agricultural Research Center, Chinese Academy of Agricultural Sciences, Changji, 831100 Xinjiang China

**Keywords:** Verticillium fungi; GWAS; Introgression; NLR receptors; Autoactivity; Self-association

## Abstract

**Background:**

Verticillium wilt is one of the most devasting diseases for many plants, leading to global economic loss. Cotton is known to be vulnerable to its fungal pathogen, *Verticillium dahliae*, yet the related genetic mechanism remains unknown.

**Results:**

By genome-wide association studies of 419 accessions of the upland cotton, *Gossypium hirsutum*, we identify ten loci that are associated with resistance against Verticillium wilt. Among these loci, *SHZDI1*/*SHZDP2*/*AYDP1* from chromosome A10 is located on a fragment introgressed from *Gossypium arboreum*. We characterize a large cluster of Toll/interleukin 1 (TIR) nucleotide-binding leucine-rich repeat receptors in this fragment. We then identify a dual-TIR domain gene from this cluster, *GhRVD1*, which triggers an effector-independent cell death and is induced by *Verticillium dahliae*. We confirm that *GhRVD1* is one of the causal gene for *SHZDI1*. Allelic variation in the TIR domain attenuates GhRVD1-mediated resistance against *Verticillium dahliae*. Homodimerization between TIR1-TIR2 mediates rapid immune response, while disruption of its αD- and αE-helices interface eliminates the autoactivity and self-association of TIR1-TIR2. We further demonstrate that GhTIRP1 inhibits the autoactivity and self-association of TIR1-TIR2 by competing for binding to them, thereby preventing the resistance to *Verticillium dahliae*.

**Conclusions:**

We propose the first working model for TIRP1 involved self-association and autoactivity of dual-TIR domain proteins that confer compromised pathogen resistance of dual-TIR domain proteins in plants. The findings reveal a novel mechanism on *Verticillium dahliae* resistance and provide genetic basis for breeding in future.

**Supplementary Information:**

The online version contains supplementary material available at 10.1186/s13059-023-02950-9.

## Background

Host-adapted microbes have evolved complex systems to produce effectors that suppress pattern recognition receptor (PRR)-triggered immunity (PTI) better to parasitize host plants. In response, plants have evolved intracellular nucleotide-binding site leucine-rich repeat (NLR) receptors, also known as *R* genes, to trigger a second line of resistance that relies on intracellular effector recognition named effector-triggered immunity (ETI) [1, 2]. In some cases, the physical NLR-effector interaction underlies the specificity of disease resistance [3–5]. Programmed host cell death (PCD), a hallmark of ETI, is categorized as a hypersensitive response (HR) at the infection site to limit the spread of the pathogen [6].

Pathogenic fungi can cause disease symptoms in many plant species and lead to serious agricultural and economic losses [7]. For example, Verticillium wilt (VW), caused by the soil-borne vascular fungi *Verticillium dahliae*, is considered a menace for many plants; it has been found in more than 400 plant species, including economically important crops such as cotton, cabbage, cauliflower, lettuce, potatoes, strawberries, tomatoes, and watermelon [8, 9]. *V. dahliae* enters plants through the roots and propagates massively in the vascular system, eventually displaying typical VW symptoms such as desiccated leaf mesophyll and severe browning of the vascular bundle [10]. Due to its complicated pathogenic mechanisms and long-term persistence in the soil, *V. dahliae* is difficult to control and has become a major threat to cotton production [11].

Understanding the genetic basis of plant-pathogen interactions is crucial for controlling plant diseases. However, since most disease tolerance traits are controlled by many genes that each produce small effects, only a small number of loci or genes have been identified. This includes the tomato immune receptor *Ve1*, which confers resistance against race 1 strains of *V. dahliae* [12]. Genome-wide association study (GWAS) is a method to uncover the genetic architecture underlying the host’s innate immune system on a population-level scale. GWAS have been used to identify the genetic basis for different plant-pathogen interactions, including *Arabidopsis thaliana-Botrytis cinerea*, *Oryza sativa-Magnaporthe oryzae*, and *Zea mays-Cochliobus heterostrophus* [13]*.* Although several studies have reported different genotyping associated with VW resistance in upland cotton (*Gossypium hirsutum*), much is still unknown about genes underlying VW resistance owing to a lack of functional validation [14–17].

To improve plant resistance against pathogenic fungi, introgression breeding has been successfully used to develop improved cultivars in many crops, including wheat [18], barley [19], tomatoes [20], rice [21], and maize [22]. Similar efforts have been invested in cotton, though the narrow genetic base of *G. hirsutum* has been a bottleneck for cotton breeding, especially for resistance breeding [23, 24]. Still, triple hybridization (*Gossypium thurberi* × *G. arboreum* × *G. hirsutum*) has been successfully used in the Pe-Dee germplasm program, and the Pe-Dee germplasm resources have been shown to have good resistance to VW [25]. However, identification of the functional introgression is time-consuming and requires high-quality genomes from both donor and recipient species, and most of these introgression breeding efforts were performed long before cotton genome sequences were available [26]. To date, unknown exotic fragments and causal genes responsible for these traits are scattered among different germplasms and across different genomic regions. Due to a lack of well-characterized quantitative trait loci (QTL) or causal genes linked to these exotic fragments for VW, it is difficult to use these exotic introgressed fragments in molecular marker-assisted selection. Therefore, identifying exotic fragments and their causal genes is essential for both resistance breeding and for studying host–pathogen interactions.

We performed GWAS on VW disease index (DI) and disease plant percentage (DP) in two environments, and a total of 10 major loci were identified. Of these, the locus on A10 carrying a large *TIR-NBS-LRR* gene cluster was introgressed from *G. arboreum*. The dual-TIR domain protein *GhRVD1* was filtered as a plausible causal gene underlying the A10 locus. Overexpression and knockdown of *GhRVD1* in cotton confirmed that *GhRVD1* is associated with VW resistance and induces significant constitutive defense activation which is commonly observed in autoimmune mutants. Allelic variation in the TIR domain attenuated the *GhRVD1-*mediated *V. dahliae* resistance. Further experimental evidence demonstrated that self-association and autoactivity of TIR1-TIR2 depends on the αD- and αE-helices (i.e., the DE) interface, and that self-association of TIR1-TIR2 was mediated by physical interaction between the TIR1 domain and TIR2 domain in different TIR1-TIR2. We found that *TIRP1* in the FK506 Binding protein (*FKBP*) family could inhibit autoactivity and self-association of TIR1-TIR2 by competing for binding to it, and negatively regulates cotton resistance to *V. dahliae* by inhibition of *GhRVD1* oligomerization. We suggest the first *TIRP1*-regulated model of the molecular events for self-association, autoactivity, and pathogen resistance of dual-TIR domain protein in plants. Germplasm resource developed here can be used for both functional genomics research and applied cotton breeding.

## Results

### Genome-wide association studies of Verticillium wilt resistance in a core collection of *G. hirsutum*

To identify loci responsible for VW resistance in upland cotton, we collected the VW resistance phenotypes from a core collection of *G. hirsutum* germplasm resources in the disease nursery at two different environments Anyang and Shihezi. Both DP and DI were used to evaluate the VW resistance of each accession in the panel. The terms AYDP and SHZDP represent the DP in Anyang and Shihezi, respectively. Similarly, AYDI and SHZDI represent DI in Anyang and Shihezi. The Pearson correlation showed strong correlation (*r* values ranging from 0.59 to 0.88) among the four traits (Additional file 1: Fig. S1c).

**Fig. 1:**
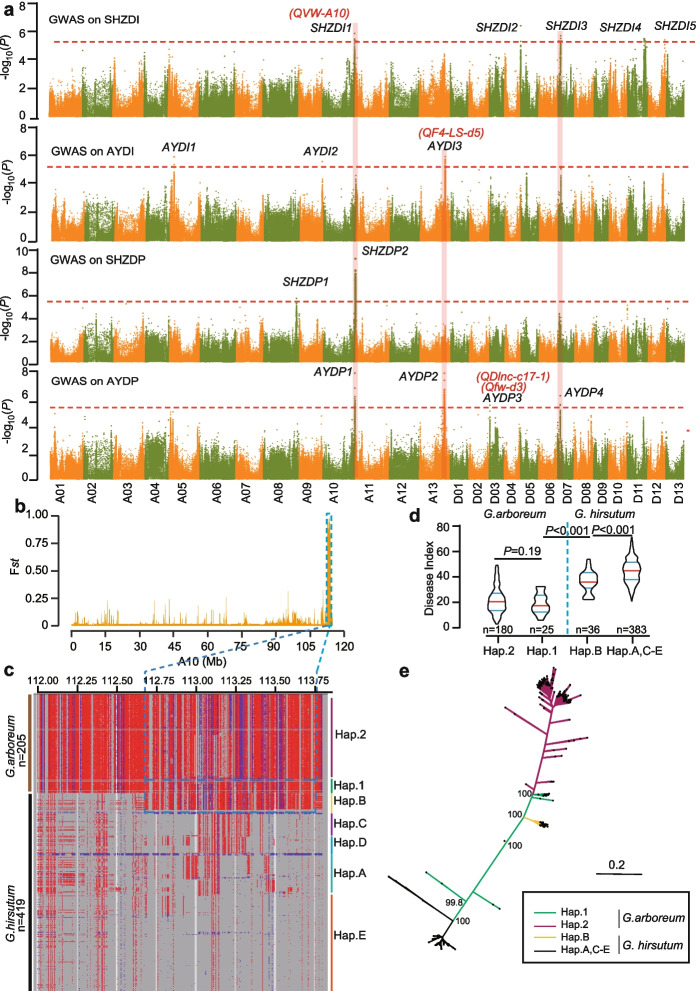
Genome-wide association using SNPs and introgression analysis on *SHZDI1*/*SHZDP2*/*AYDP1.* **a** Manhattan plot for GWAS on the Verticillium wilt for disease percentage (DP) and disease index (DI) in samples from Shihezi and Anyang. High-confidence QTLs and previously reported QTLs are marked in black and red words, respectively. **b** F_*st*_ analysis of A10 between the tolerant group and susceptible group based on lead SNP of *SHZDI1*. **c** SNP schematic around *SHZDIP1* showing 2 haplotypes (Hap. 1 and Hap. 2) in *G. arboreum* and 5 haplotypes (Hap. A to Hap. E) in *G. hirsutum*. Number of materials is marked on the left. Hap.B of *G. hirsutum* carrying the introgressed *G. arboreum* fragments of Hap.1. Blue box indicates an introgressed region. **d** DI of haplotypes in greenhouse-grown *G. arboreum* and *G. hirsutum*. Significant difference was measured between different groups (Student’s *t* test). *G. hirsutum* carrying the introgressed fragments showed significantly enhanced VW resistance compared to materials without these fragments. *G. arboreum* variants carrying either Hap. 1 or Hap. 2 were more tolerant than *G. hirsutum.* **e** SNP-based phylogenetic tree showing that Hap.B lines of *G. hirsutum* harbor the introgression from A10 of *G. arboreum*

We next performed GWAS on AYDP, AYDI, SHZDP, and SHZDI using EMMAX [27] to identify the loci underlying each trait. The latest high-quality *G. hirsutum* TM-1 genome was used as the reference genome (CRI-TM-1, version1.0) [28]. First, all the traits’ genome-wide significance thresholds were set to a uniform threshold (5.03E − 6, *P* = 1/*n*, where *n* is the effective number of independent SNPs). As a result, 4 loci (*AYDP1*, *AYDP2*, *AYDP3*, and *AYDP4*) from A10, A13, D03, and D07, respectively, were identified for AYDP. For SHZDP, 2 loci (*SHZDP1* and *SHZDP2*) from A08 and A10, respectively, were identified. For AYDI and SHZDI, 3 and 5 loci were detected respectively (Fig. 1a, Additional file 1: Fig. S2b and S3a, and Additional file 2: Table S1). Remarkably, *AYDP1*, *AYDP2*, and *AYDP3* overlapped with previously reported QTL regions associated with VW or Fusarium wilt resistance (Fig. 1a) [15, 29]. Additionally, *SHZDI1* was co-located with *SHZDP2* and *AYDP1*, *SHZDI3* was co-located with *AYDP4*, and *AYDI3* was co-located with *AYDP2*, which provides important loci for targeted-genotyping-based selection.

### *SHZDI1* originated from an exotic introgressed fragment of *G. arboreum*

Based on the allelic genotypes of the lead SNP of *SHZDI1/SHZDP2/AYDP1* (Additional file 1: Fig. S1d), we divided the GWAS panel into 2 groups: tolerant and susceptible. We found that the *F*_*st*_ value between the two groups significantly increased on A10 ranging from 112.54 to 114.07 Mb (F_*st*_ peak = 0.982) (Fig. 1b). This indicates that the tolerant and susceptible groups demonstrated strong differentiation in this region. Since exotic introgression can result in strong differentiation, we speculated whether this observation is caused by introgression or not.

The triple hybrid (*G. arboreum* × *G. thurberi* × *G. hirsutum*) and *Gossypium barbadense* have been widely used in upland cotton breeding [25, 30]. First, given the knowledge that *G. thurberi* is a diploid D-genome cotton, we can safely assume that any introgressions from it will be vastly more likely into the upland cotton Dt subgenome (i.e., than the At subgenome). Given that the introgression-of-interest in our study located on chromosome A10 of the At subgenome, we made the assumption that *G. thurberi* was exceedingly unlikely to be the donor for A10 introgressions, thus narrowing the field of donors to *G. arboreum* and *G. barbadense*. Following a recent reported method for identification introgression [26, 31], we separately analyzed the introgressed fragments from *G. arboreum* and from *G. barbadense*. Fortunately, this analysis indicated that *SHZDI1* shared overlap with a fragment from *G. aboreum* and shared no overlap with *G. barbadense* (Additional file 2: Table S8), findings supporting that the introgressions are from *G. arboreum.* A total of 36 accessions carried introgressions were identified in the GWAS panel. To further confirm the above results, we identified the SNPs between a core collection of *G. arboreum* germplasm resources generated by our previous report [32] with the GWAS panel on A10 (i.e., SNP-A10). The genotypes in the *SHZDI1/SHZDP2/AYDP1* locus can be divided into 2 haplotypes (Hap. 1 and Hap. 2) in *G. arboreum* and into 5 haplotypes (Hap. A to Hap. E) in *G. hirsutum* (Fig. 1c). We found that Hap. B is highly similar to Hap.1, indicating Hap. B introgressed from *G. arboreum*. We next constructed a SNP-based phylogenetic tree using the SNPs from the putatively introgressed genomic region (highlighted in the blue box in Fig. 1c). The phylogenetic tree indicated that accessions carrying Hap. B were clustered with accessions carrying Hap. 1, further supporting that Hap. B is introgressed from *G. arboreum* (Fig. 1e and Additional file 1: Fig. S2a). In previous studies, *G. arboreum* possesses *Verticillium dahliae*, *Fusarium oxysporum vasinfectum*, and cotton leaf curl virus resistance characteristics unavailable in the tetraploid cultivated cotton gene pool [33]. Compared with accessions carrying Hap. 1, the accessions carrying Hap. 2 demonstrated no difference in VW resistance (Fig. 1d). However, when compared with *G. hirsutum*, accessions carrying either Hap. 1 or Hap. 2 were more tolerant to *V. dahliae*, indicating that *G. arboreum* is more tolerant to *V. dahliae* than *G. hirsutum* (Fig. 1d). We also observed that the accessions carrying Hap. B were more tolerant than those carrying Hap. A, C, D, or E (Fig. 1d and Additional file 1: Fig. S1f). Our results indicated that the introgression from *G. arboreum* can increase the tolerance of *G. hirsutum* to *V. dahliae*. Given that only 8.5% of accessions in the core collection of *G. hirsutum* germplasm carry these introgressed fragments, this favorable allele has not been widely used in cotton breeding and could be an important resource for improving VW resistance in cotton.

### A large *TIR-NBS-LRR* gene cluster on *SHZDI1* contributes to Verticillium wilt resistance

**Fig. 2:**
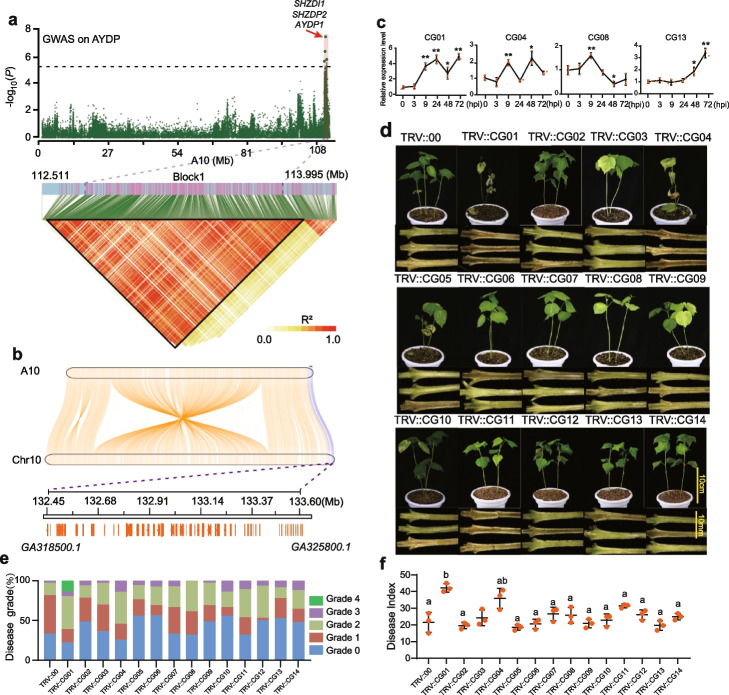
Identification of candidate genes associated with Verticillium wilt resistance on introgressed fragments (SHZDI1/*SHZDP2*/*AYDP1*). **a** Manhattan plot for *AYDP1* on chromosome A10 and haplotype blocks (A10:112.511–113.995 Mb) around *SHZDI1/SHZDP2/AYDP1* (A10:112.70–113.75 Mb) was estimated using pairwise LD correlations (*R*^2^). **b** Collinearity analysis of A10 of *G. hirsutum* and Chr10 of *G. arboreum* is shown in mauve. *SHZDI1/SHZDP2/AYDP1* (A10:112.70–113.75 Mb) in *G. hirsutum* corresponded to 132.45 to 133.60 Mb on Chr10 of *G. arboreum.* Corresponding homology to *SHZDI1/SHZDP2/AYDP1* is marked on the purple line. **c** Expression patterns of 4 of 14 candidate genes at 0, 3, 9, 24, 48, and 72 h post-inoculation (hpi) with *V. dahliae* strain Vd080 in introgressed Hap. B (ZZ2), measured by qRT-PCR. **d–f** Silencing of candidate genes in cotton seedlings inoculated with Vd080. Plant wilt phenotype and stem browning symptoms were photographed at 3 weeks post-inoculation. Percentages were counted for each disease grade (increasing disease severity from grade 0 to 4). DI was the mean value of three independent experiments and was measured at 21 days post-inoculation (dpi). TRV::*00* was used as a negative control, and one-way ANOVA was used in the statistical analysis, and means labeled with different letters indicate significant difference at *α* = 0.05. Error bars represent the SD of three biological replicates

The genomic spectrum of *SHZDI1/SHZDP2/AYDP1* ranges from 112.7 to 113.75 Mb on A10 (Fig. 2a). To find the corresponding homologous section on *G. arboreum*, we extracted 2 Mb genomic sequences (A10:112–114 Mb) of TM-1 and cut them into 1 kb fragments to blast with *G. arboreum* genomic sequence. The *SHZDI1* locus in *G. hirsutum* corresponded to 132.45 to 133.60 Mb region on Chr10 of *G. arboreum* (Fig. 2a, b). A total of 74 genes locate in this region, of which 42 genes belong to the Toll/interleukin 1 receptor (TIR) NLR (TNL) family or its truncated genes that play an integral role in the host immune system by triggering ETI response [2, 34] (Additional file 2: Table S4 and S5).

Since inducible defense signaling pathways play important roles in cotton defense against *V. dahliae* [10, 35], we set to identify candidate genes within the introgressed fragments (Additional file 1: Fig. S4a and Additional file 2: Table S6). By using the mRNA-seq data from plants under *V. dahliae* treatment, we identified 13 genes (CG1 to CG13) that were categorized as differentially expressed genes (DEGs) (adjusted *P* < 0.01 and absolute fold-change > 2). The expression levels of these genes were further confirmed by quantitative PCR (Fig. 2c and Additional file 1: Fig. S4e), which showed that CG01 had the highest upregulation with a 4.92-fold-change (Fig. 2c).

N-terminal region of functional TNLs, which contains a TIR domain, executes cell death response upon effector recognition, of which truncated TIR domain alone can signal effector-independent cell death response when expressed ectopically in tobacco leaves [36–38]. We annotated TIR domain in the 42 truncated or typical TNLs, and 28 genes encoding TIR domain were identified (Additional file 1: Fig. S4b and Additional file 2: Table S5). Next, the TIR domain from 28 genes as well as the positive (*RPP1_NdA*^TIR^) and negative controls (empty vector or buffer) were transiently expressed in tobacco leaves respectively. It is interesting that only the truncated TIR domain from GA10G321300 (CG01), GA10G322400 (named CG14 but not belong to 13 DEGs), and the positive control triggered effector-independent cell death, which suggests that CG01 and CG14 have potential functions in ETI signaling pathways (Additional file 1: Fig. S4b).

To validate the potential function of the 14 candidate genes (CG01-CG14), we performed virus-induced gene silencing (VIGS) experiments. qPCR results showed that CG14 had no detectable expression, and the expression of the other 13 candidate genes was reduced significantly in cotton (Additional file 1: Fig. S4c). Plants carrying TRV::CG01 or TRV::CG04 displayed typical symptoms of VW (Fig. 2d-f). Considering the possibility of off-target silencing, the expression of two potential off-target sites with the highest homology were measured by qPCR; the transcripts of these genes were not affected in VIGS experiments (Additional file 1: Fig. S4d). Additionally, the silencing of CG01 made the introgressed resistant accession B061 (ZZ2) susceptible to *V. dahliae* with a highest disease index of 42.1 (Fig. 2f).

In whole, functional validation results indicate that the VW resistance mediated by introgressed fragments was associated with multiple genes in this region. Given the highly upregulated expression after inoculation, potential functions in ETI response, and the severe knockdown symptoms, CG01 was identified as one of the major VW-associated genes in this candidate region. Hereafter, we will refer to CG01 as *Resistant to Verticillium dahliae 1* in *Gossypium hirsutum* (*GhRVD1*).

### Allelic variation in TIR domain attenuates *GhRVD1*-mediated *VW* resistance

**Fig. 3:**
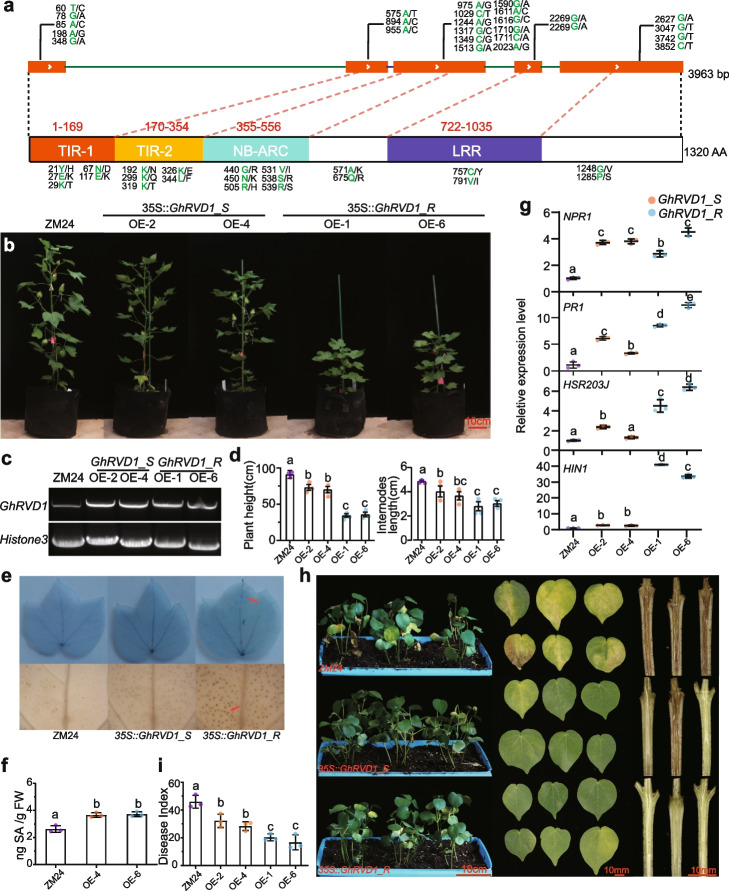
Highly divergent *GhRVD1 *between Hap. B and Hap. E. **a** An illustration of a 26 base point mutation in the CDS, and 22 nonsynonymous mutations in amino acid sequences among *GhRVD1_R* and *GhRVD1_S*; R and S are shown in green and black words, respectively. Mutation positions are marked on the top and bottom. **b** Phenotype of *GhRVD1_R* and *GhRVD1_S* overexpression cotton lines. **c** Transcript detection of both *GhRVD1* genotypes using semi-quantitative PCR with universal primers; *GhHiston3* was used as an internal control. **d** Phenotypes with a significant reduction of plant height and internode distances. Different letters indicate significant difference at *α* = 0.05 level via one-way ANOVA analysis. **e** Trypan blue staining to detect dead cells (top), and DAB staining to detect accumulation of ROS species (bottom) in 10-week-old sample leaves. **f** SA content of 10-week-old sample leaves via HPLC. **g** Expression of SAR markers (e.g., *NPR1* and *PR*1) and HR markers (e.g., *HIN1* and *HSR203J*) in *GhRVD1* overexpression lines from leaves collected from 10-week-old plants. **h** Plant wilt phenotype, leaves wilt phenotype, and stem browning symptoms were photographed at 3 weeks post-inoculation. **i** DI was the mean value of three independent experiments and was measured at 21 dpi

*GhRVD1* was cloned from Hap. B (B014, ZZ2, and F045) and Hap. E (B009, B010, and ZM24) for sequence analysis. As expected, the sequences of *GhRVD1* were highly divergent between Hap. B and Hap. E, with 22 nonsynonymous mutations out of 26 SNPs that were named *GhRVD1_R* (resistant) and *GhRVD1_S* (susceptible), respectively (Fig. 3a). Three domains, including the TIR domain (PFAM01582), NB-ARC domain (CL26397), and LRR domain (PFAM12799), were identified in *GhRVD1* (Fig. 3a and Additional file 1: Fig. S5a). The two haplotypes of *GhRVD1* were overexpressed in *Arabidopsis thaliana* to generate the *GhRVD1_R* and *GhRVD1_S* overexpression lines (Additional file 1: Fig. S5c). *GhRVD1_R* overexpression lines demonstrated enhanced resistance to *V. dahliae*, compared with WT and *GhRVD1_S* lines. Interestingly, *GhRVD1_R* lines demonstrated an obvious dwarfing phenotype (Additional file 1: Fig. S5c). Subsequently, two artificial transcripts of a combination of *GhRVD1_S* and *GhRVD1_R, GhRVD1_R/S* and *GhRVD1_S/R*, were also overexpressed in *A. thaliana*. *GhRVD1_R/S* was developed by replacing the TIR1-TIR2 (1-354AA) from *GhRVD_S* with that of *GhRVD_R*. Likewise, *GhRVD1_S/R* was developed by replacing the TIR1-TIR2 from *GhRVD1_R* with that of *GhRVD_S*. We found that overexpression of *GhRVD1_R/S,* like overexpression of *GhRVD1_R*, conferred *A. thaliana* VW resistance, while overexpression of *GhRVD1_S/R* showed susceptible phenotyping when compared with transgenic *A. thaliana* carrying *GhRVD1_R* (Additional file 1: Fig. S5e-g)*.* The results indicated that the first 10 nucleotide substitutions at the position of TIR domain in the background of *GhRVD1_R* attenuated the acquired *V. dahliae* resistance.

### Overexpression of *GhRVD1* shows constitutive defense activation in cotton

We also overexpressed the two *GhRVD1* haplotypes in cotton and found that 35S::*GhRVD1_R* induced an obvious dwarfing phenotype with a significant reduction of plant height by 61.3% and of internode distances by 40.2% (Fig. 3b–d). Dwarfing mutants have been found in cotton as well as many other plants, and brassinolide as well as gibberellin (GA) are the two major hormones associated with dwarfing [39–41]. Moreover, hyperactivity of plant innate immune receptors often cause ectopic defense activation, also called autoimmunity, which can manifest as severe growth retardation and spontaneous lesion formations. This phenotype is most widely studied in *A. thaliana snc1* dwarfing mutant, a gain-of-function NLR mutant with constitutive defense activation [42–44]. Similar to the autoimmunity, overexpression of *GhRVD1* induced a constitutive accumulation of reactive oxygen species (ROS) and salicylic acid (SA) and caused spontaneous lesions in 35S::*GhRVD1_R* (Fig. 3e, f). Hypersensitive responses (HR) induced by R genes are accompanied by several active physiological responses that restrict pathogen colonization and activate the expression of HR markers such as *HIN1* and *HSR203J* [43, 45], both of which were significantly upregulated in all *GhRVD1* overexpression lines. Moreover, the expression of *HSR203J* and *HIN1* in 35S::*GhRVD1_R* was significantly enhanced compared to 35S::*GhRVD1_S* (Fig. 3g). The R gene-mediated HR also often triggers a secondary resistance response known as systemic acquired resistance (SAR), which is characterized by upregulation of *NPR1* and *PR1* [43], and we found that the expression of *NPR1* and *PR1* in *GhRVD1* overexpression lines was significantly enhanced compared to WT (Fig. 3g). These results support that the accumulation of *GhRVD1* transcripts, especially of the *GhRVD1_R* haplotypes, result in HR responses and an active SAR signaling, thus conferring the observed autoimmune phenotypes.

Subsequently, 35S::*GhRVD1_S*, 35S::*GhRVD1_R*, and ZM24 (negative control) plants were infected with Vd080 to evaluate the gene function. Different from the case for *A. thaliana*, overexpression *GhRVD1_R* or *GhRVD1_S* can both enhance resistance to VW in *G. hirsutum*, but a more significant enhancement of VW resistance was observed in *GhRVD1_R* overexpression lines (Fig. 3h, i).

### Allelic variation in the TIR domain affects autoactivity of *GhRVD1*

Plant NLRs characterized by a multi-domain architecture consisting of either an N-terminal coiled-coil (CC) or TIR domain, a central NBS domain, and C-terminal LRR domain [46]. Typical TNLs usually have one TIR domain in the N-terminal of a protein. However, unlike the typical TNLs, GhRVD1 has two consecutive TIR domains (PF01582) located in the first 354 amino acids that share 56.4% amino acid similarity with each other, which arose by gene amplification inside cotton (Fig. 3a and Additional file 1: Fig. S6a). We screened the identified TIR domains for a more detailed comparison [36]. The first one located in the first 169 amino acids of the N-terminal, and the second TIR domain located adjacent to the first one (from 170 to 354 AA). These two TIRs from *GhRVD1_R* were designated as R-TIR1 and R-TIR2. Likewise, the dual-TIR domains from *GhRVD1_S* were named S-TIR1 and S-TIR2 in the same pattern (Fig. 3a and Additional file 1: Fig. S5b). In whole, this 354 AA region includes the TIR1 and TIR2 domains, designated as “TIR1-TIR2_R” or “TIR1-TIR2_S”.

TIR domains with known 3D structures consist of a five-stranded parallel β-sheet (βA–βE) surrounded by five α-helical regions [36]. Protein modeling with Phypre2 at 90% accuracy indicated that TIR1-TIR2_R and TIR1-TIR2_S contain most of the secondary structures in known 3D structures, except for the βB-sheet of TIR1-TIR2 and βE-sheet for TIR1 (Additional file 1: Fig. S5b). Moreover, two haplotypes of TIR1-TIR2 were detected with 10 nonsynonymous mutations in amino acid sequences (Fig. 3a). The combined mutations are predicted to result in the disappearance of αE1-helices and completely change the 3D structure of TIR1-TIR2 (Additional file 1: Fig. S5b and 6b).

**Fig. 4:**
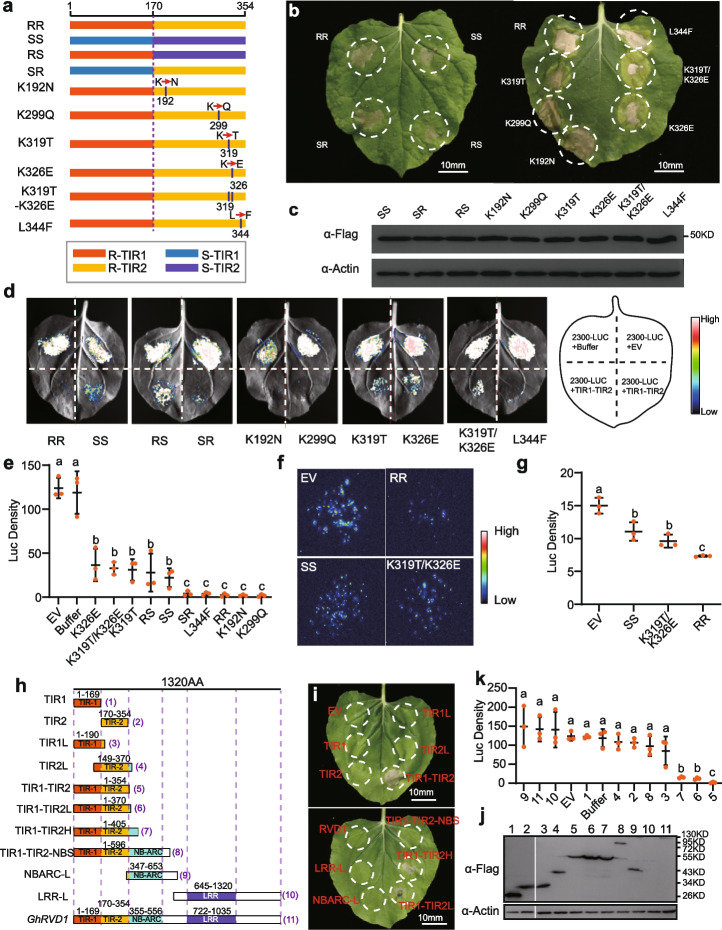
Nonsynonymous SNP sites of 319 and 326 in TIR1-TIR2 caused differences in HR strength between the two haplotypes, and the minimum functional region is TIR1-TIR2. **a,b** HR phenotypes associated with chimeras or site-directed mutants of TIR1-TIR2_R and TIR1-TIR2_S alleles. RS and SR are artificial constructs of a combination of R-TIR1-S-TIR2 and S-TIR1-R-TIR2, respectively. Site-directed mutants were converted to S alleles at the nonsynonymous SNP sites in a TIR1-TIR2_R background. **c** Evaluation of protein expression using a Flag antibody, and immunoblotting of plant actin with α-actin was used as a loading control. **d,e** LUC activity within the region co-transformed with a LUC plasmid and TIR1-TIR2 variants. Fluorescence intensity was captured and data represent means ± SE of three independent experiments. Different letters indicate significant difference at *α* = 0.05 level via one-way ANOVA analysis. **f,g** Cell death phenotype in cotton protoplast for cell viability assays. Protoplast preparation and construct transformation used 14-day-old plant leaves. Fluorescence intensity was captured. Three independent experiments are represented. **h** Schematic diagram of the *GhRVD1* domain structure. Individual domains are presented in a colored box, and the boundaries of truncated sequences are marked on the top. The numbers and names of truncated sequences are marked on the right and left, respectively. **i** HR phenotypes of truncated derivatives of *GhRVD1_R*.** j** Evaluation of protein expression using a Flag antibody, immunoblotting of plant actin with α-actin was used as a loading control. **k** LUC activity within the region co-transformed with a LUC plasmid and a series of truncated derivatives of *GhRVD1.* Numbers on the top represent corresponding truncated sequences

In previous experiments, the autoactivity of TIR1-TIR2_R (CG01^TIR^) was first identified in cotton NLRs (Additional file 1: Fig. S4b), and we further investigated the divergence of autoactivity between R and S haplotypes using fusion proteins and a series of mutations. We found the intensity of cell death caused by TIR1-TIR2_S (SS) is significantly weaker than that caused by TIR1-TIR2_R (RR). To confirm whether a single TIR or both TIRs were responsible for the difference in cell death intensity, we artificially created two mutants: TIR1-S-TIR2-R (SR) and TIR1-R-TIR2-S (RS) in TIR1-TIR2_R background. When TIR1-R was mutated to TIR1-S by mutating 5 nucleotides, the cell death intensity of the first mutant SR has no difference with that of RR. However, when TIR2-R was mutated to TIR2-S by mutating 5 nucleotides, the cell death intensity of the second mutant RS significantly decreased (Fig. 4a,b and Additional file 1: Fig. S7a). Trypan blue detection of death cells also showed that RR and SR induced higher intensity of cell death compared with both SS and RS (Additional file 1: Fig. S8). The firefly Luciferase (LUC) reporter gene system has been a powerful tool to monitor the cell viability through quantifying the fluorescence activity in many studies [47–50]. Here, we also co-expressed the candidate proteins and LUC to perform the cell death assay. Both the fluorescence signal and luciferase activity again confirm that RR and SR induced stronger intensity of cell death than both SS and RS (Fig. 4d,e and Additional file 1: Fig. S6h). Viewed together, our results suggest that the HR response’s intensity enhanced in a manner depended on the mutations between the TIR2-R and TIR2-S.

Following this reasoning, K192N, K299Q, K319T, K326E, K319T/K326E, and L344F were mutated at the nonsynonymous SNP sites of TIR2 in a TIR1-TIR2_R background (Fig. 4a) and were expressed ectopically in tobacco leaves. The results demonstrate that amino acid substitutions at positions 319 or 326 attenuated the HR response (Fig. 4b and Additional file 1: Fig. 7a), which locate in α-helix E1 and α-helix E2 of TIR2, respectively (Additional file 1: Fig. S6b), but the strength of cell death was not further attenuated in K319T/K326E. Mutations at these two amino acids caused differences in HR strength between the two haplotypes of TIR1-TIR2. Protein immunoblotting demonstrated that all fusion or mutant proteins were properly expressed in tobacco leaves (Fig. 4c). Subsequently, trypan blue assay (Additional file 1: Fig. S8) and fluorescence intensity (Fig. 4d,e) again supported that amino acid substitutions at position 319 or 326 of TIR2, respectively, attenuated the HR response.

To verify that TIRs can result in cell death in cotton, TIR1-TIR2 variants and 35S::Luciferase were simultaneously expressed in cotton protoplasts. The decrease of the fluorescence signal indicates that TIR1-TIR2 also caused cell death in cotton protoplasts, and the cell death caused by SS and K319T/K326E was significantly weaker than that of RR (*P* < 0.01) (Fig. 4f,g). Our results indicate that the two haplotypes of TIR1-TIR2 from *GhRVD1* can induce cell death, and nonsynonymous SNP sites 319 and 326 of TIR1-TIR2 play an important role in controlling the intensity of cell death.

### Defining the functional boundaries of the *GhRVD1* TIR domain

To clarify the functional boundaries of the TIR domain, a series of truncated *GhRVD1* derivatives were constructed and transiently expressed in tobacco leaves (Fig. 4h). We found that NBS-ARC sequences connected after the dual-TIR domains can significantly inhibit cell death, which was similar to the previous report assessing *RPP1_NdA* [37]. Notably, neither TIR1 nor TIR2 alone could induce cell death, and the smallest fragment that induced cell death was amino acid 1–354 of *GhRVD1*, which precisely covered the full length of the TIR1 and TIR2 domain sequences (Fig. 4h–j and Additional file 1: Fig. S7b). With the exception of LRR-L, all proteins were detectable by protein immunoblotting, although the steady-state accumulation of each protein differed substantially (Fig. 4j). Our results suggest that this 354 AA region, comprising “TIR1-TIR2,” is the minimal sequence required for autoactivity and was inhibited by sequence ligation of the NB-ARC domain.

### Mutations at the predicted DE interface disrupt autoactivity and self-association of TIR1-TIR2

**Fig. 5:**
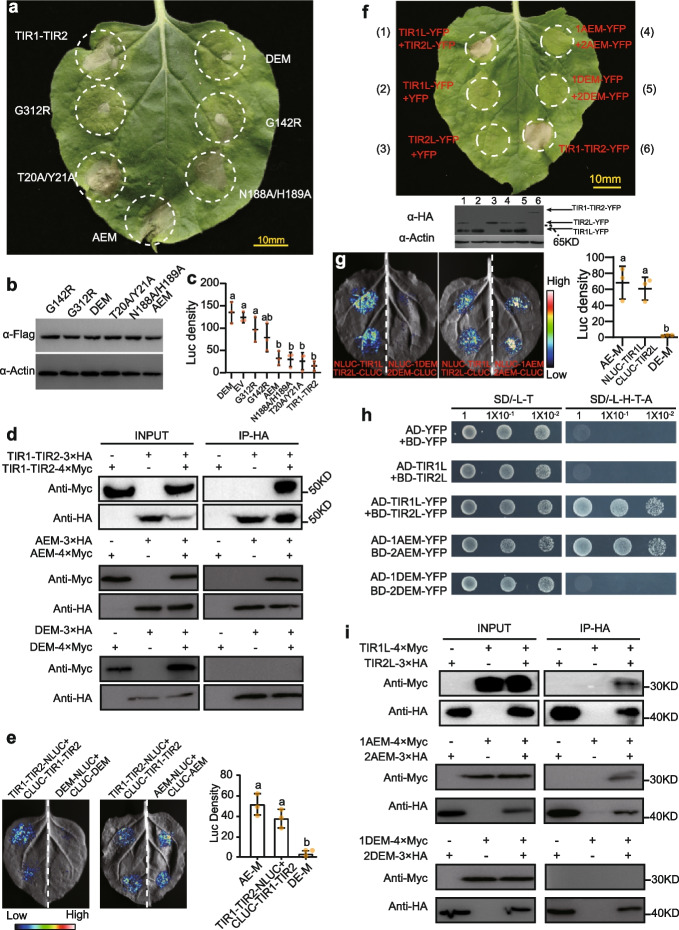
Self-association of TIR1-TIR2 and physical interaction between TIR1 and TIR2 depend on the DE interface and cause cell death. **a** HR phenotypes associated with site-directed mutants of TIR1-TIR2_R, T20A/Y21A, and N188A/H189A disrupting the AE interface of TIR1 and TIR2, respectively; G142R and G312 disrupting the DE interface of TIR1 and TIR2, respectively. AEM disrupts the AE interface of TIR1 and TIR2, and DEM disrupts the DE interface of TIR1 and TIR2. Autoactivity of TIR1-TIR2_R is disrupted by mutations in the predicted DE interface. **b,c** Evaluation of protein expression using a Flag antibody, immunoblotting of plant actin with α-actin was used as a loading control. LUC activity within the region is co-transformed with a LUC plasmid and TIR1-TIR2_R variants. Different letters indicate significant difference at *α* = 0.05 level via one-way ANOVA analysis. **d** Coimmunoprecipitation analysis of self-association of TIR1-TIR2_R and its variants. The indicated proteins were transiently expressed in *N. benthamiana* leaf cells by agroinfiltration. Total protein extracts were used for coimmunoprecipitation and immunoblot analyses. **e** Split luciferase complementation shows an interaction between TIR1-TIR2_R (left) and the interaction strength between TIR1-TIR2_R variants (right). Fluorescence signal intensity is indicated. **f** HR phenotypes associated with co-inoculation of TIR1L_R-YFP-HA and TIR2L_R-YFP-HA and that of their variants (1AEM and 2AEM disrupts the AE interface of TIR1L and TIR2L, respectively; 1DEM and 2DEM disrupts the DE interface of TIR1L and TIR2L, respectively). TIR1-TIR2-YFP-HA was used as positive control (top). Evaluation of protein expression using a HA antibody, and immunoblotting of plant actin with α-actin was used as a loading control (bottom). **g** Split luciferase complementation indicates the interaction between TIR1L_R and TIR2L _R (left), and the interaction strength between their variants (right). Fluorescence signal intensity is indicated. **h** Growth of yeast cells co-expressing AD-TIR1L_R-YFP and BD-TIR2L_R-YFP fusions, and their variants, on synthetic media lacking leucine and tryptophan (− LT) or selective media additionally lacking histidine and adenine (− LTHA). A series of dilutions are shown at the top of the figure. Autoactivation and toxicity of the fusion protein were first excluded, and AD-YFP and BD-YFP were used as a negative control for self-association activity. **i** Coimmunoprecipitation analysis of the interaction between TIR1L_R and TIR2L_R and that of their variants

Plant TNL activation leads to homodimerization of the intracellular TIR domain and initiates the downstream signaling pathway [2, 51]; this self-association is also present in the effector-independent cell death of the TIR domain autoactivation phenotype [1, 36, 46]. In a previous study, predicted αA- and αE-helices (AE) and DE interfaces were found to be involved in the homodimerization of the plant TIR domain [1, 36, 52]. Based on homologous alignment with the well-characterized TIR domains of *L6* [36] and *RPS4* [52], we identified key amino acid residues involved in the formation of the AE interface (T20 and Y21 from TIR1, and N188 and H189 from TIR2) or DE interface (G142 from TIR1, and G312 from TIR2) (Additional file 1: Fig. S5b). We next made a series of constructs of AE/DE mutagenesis derivatives to check the autoactivity of TIR1-TIR2 (Additional file 1: Fig. S5b), including those with the mutation of T20 and Y21 disrupting the AE interface of TIR1 (T20A/Y21A); the mutation of N188 and H189 disrupting the AE interface of TIR2 (N188A/H189A); the mutation of G142 disrupting the DE interface of TIR1 (G142R); the mutation of G312 disrupting the DE interface of TIR2 (G312R); the mutations of T20, Y21, N188, and H189 disrupting the AE interface of TIR1 and TIR2 (AEM); and the mutation of G142 and G312 disrupting the DE interface of TIR1 and TIR2 (DEM). Mutations of G312R or G142R/G312R (DEM) that disrupt the DE interface of TIR2 can abolish cell death induced by TIR1-TIR2. Although G142R of the DE interface of TIR1 failed to completely abolish autoactivity, a significantly weak autoactivation phenotype was observed in the injected area (Fig. 5a–c and Additional file 1: Fig. S7c). In contrast, mutations in the AE interfaces of both TIR1 and TIR2 do not affect the autoactivity of TIR1-TIR2 (Fig. 5a–c and Additional file 1: Fig. S7c).

We next examined the self-association of TIR1-TIR2 with luciferase complementation assays (LCA) and coimmunoprecipitation assays (Co-IP). In Co-IP assays, we immunoprecipitated TIR1-TIR2-HA proteins with anti-HA and immunoblotting with anti-Myc, and the TIR1-TIR2-Myc protein was detected only in the precipitate from the leaves that co-expressed both TIR1-TIR2-HA and TIR1-TIR2-Myc, suggesting that TIR1-TIR2 can form self-associations (Fig. 5d). Furthermore, the LCA assays demonstrated that TIR1-TIR2-NLUC was associated with CLUC-TIR1-TIR2, supporting that the TIR1-TIR2 complex can assembly by self-association (Fig. 5e).

To test whether the AE or DE interfaces were involved in TIR1-TIR2 self-association, key residues required for interface formation of dual-TIR domains were mutated (AEM and DEM). Mutations in the AE interface do not affect the TIR1-TIR2 self-association in LCA and Co-IP assays. In contrast, mutations in the DE interface were found to disrupt TIR1-TIR2 self-association in both LCA and Co-IP assays (Fig. 5d,e). In summary, our results from mutating residues responsible for homodimerization suggest that self-association is required for TIR1-TIR2 autoactivity and that this process depends on the DE interface but not necessarily the AE interface.

### Physical interaction between TIR1 and TIR2 depends on the DE interface

Many studies have demonstrated that TNLs with only a single N-terminal TIR domain require the TIRs to heterodimerize in order to induce the autoactivation phenotype [53]. However, it remains unclear how dual-TIR domains from TNL function for autoactivity. To gain further insight into the function of TIR1 and TIR2, a series of mutant TIR1 and TIR2 were transiently expressed in tobacco leaves. TIR1L was amino acids 1–190 of GhRVD1, which comprised all of TIR1 and 20 N-terminal amino acids; the extra amino acids were included to ensure TIR1 was stably and functionally expressed [36]. Similarly, TIR2L was amino acids 150–370 of GhRVD1, which comprised all of TIR2 and 16 C-terminal amino acids as well as 20 N-terminal amino acids. Additionally, we used a C-terminal YFP fusion since previous reports have demonstrated that a C-terminal YFP fusion can facilitate autoactivity of the TIR domains (Additional file 1: Fig. S9a-b) [37, 54]. When TIR1L-YFP and TIR2L-YFP were transiently expressed alone, no cell death phenotypes were induced. However, when these proteins were co-inoculated for transient expression, cell death phenotypes similar to that of the TIR1-TIR2_R domain were observed (Fig. 5f and Additional file 1: Fig. S7d). This indicates that TIR1 and TIR2 work synergistically when inducing cell death.

To verify the interaction between TIR1L and TIR2L, LCA, Co-IP, and yeast two-hybrid assays (Y2H) (with an YFP flag) were performed. We found that TIR1L was strongly associated with TIR2L in these experiments (Fig. 5g–i), while we found that TIR1L self-associated weakly, and no self-association was observed for TIR2L (Additional file 1: Fig. S6g).

To test whether the AE or DE interface was required for the interaction between TIR1L and TIR2L, key residues involved in forming the AE interface (T20, Y21, N188, and H189 from TIR1L; and N188 and H189 from TIR2L) were mutated to obtain TIR1L-AEM (1AEM) and TIR2L-AEM (2AEM). Similarly, we created constructs with mutation of key residues involved in forming the DE interface (G142 from TIR1L and G312 from TIR2L) to obtain TIR1L-DEM (1DEM) and TIR2L-DEM (2DEM). LCA, Co-IP, and Y2H assays consistently indicated that mutations in the DE interface but not the AE interface, disrupted the interaction between TIR1L and TIR2L (Fig. 5g–i).

Subsequently, mutagenesis proteins of TIR1L and TIR2L were co-inoculated for transient expression in tobacco leaves. The mutation in the DE interface was able to completely abolish cell death phenotypes induced by TIR1L and TIR2L co-inoculation. Interestingly, the AE interface mutations also abolished the cell death phenotypes in co-inoculation (Fig. 5f and Additional file 1: Fig. S7d). Our results suggest that the physical interaction between TIR1L and TIR2L requires a functional DE interface, but this alone is not sufficient for the induction of cell death when co-inoculated.

In summary, the TIR1 and TIR2 domains are necessary for the autoactivation phenotype; meanwhile, the interface which the self-association of TIR1-TIR2 depends on is highly in accordance with the interface of physical interaction between TIR1L and TIR2L. This indicates that TIR1 and TIR2 interaction mediates the self-association of TIR1-TIR2 and the subsequent signaling activity.

### A *FKBP* family protein, *TIRP1*, binds to TIR1-TIR2 *in vivo*

TIR domain-mediated immune responses are often tightly regulated in plant. Besides NBS-mediated self-inhibition, the formation of N-terminal heterogenous complexes attenuates TNL homodimerization to suppress pathogen resistance [38, 55]. In our study, 35S::*GhRVD1_R* resulted in a dwarf phenotype which was not observed in plants transformed with *G. hirsutum* expressed under its native promoter, indicating that *GhRVD1* was potentially negative regulated in *G. hirsutum* (Additional file 1: Fig. S6d). Subsequently, TIR1-TIR2 was used as bait in a Y2H library screening experiment. Sequence analysis revealed that the TIR1-TIR2 interacting protein was a homolog of *FKBP17-2* from *A. thaliana* and *Theobroma cacao* (GenBank ID: NP_564048.1 and XP_007036779.2, respectively) [56] (Additional file 2: Table S7), and it was designated as *G. hirsutum TIR domain partner protein 1* (*GhTIRP1*).

**Fig. 6:**
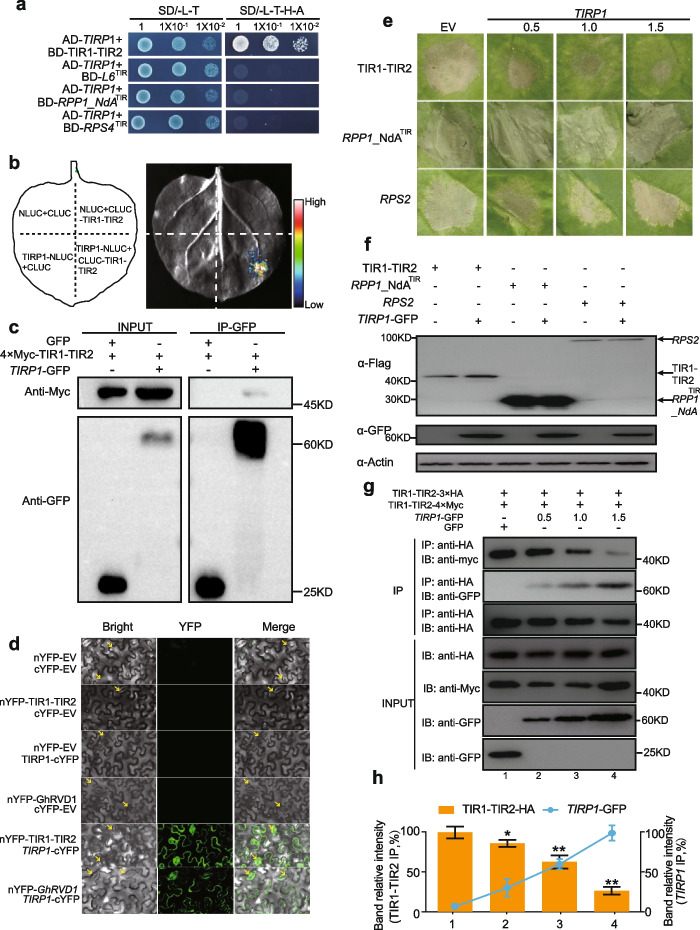
*GhTIRP1* binds to TIR1-TIR2_R in vivo and inhibits autoactivity and self-association of TIR1-TIR2_R by competing for binding to it. **a** Growth of yeast cells co-expressing AD-*TIRP1* and BD-TIR fusions on synthetic media; TIR domains of *L6* (residues 29–248), TIR domains of *RPP1_NdA* (residues 1–254), and TIR domains of *RPS4* (residues 1–183). Yeast cells co-expressing AD-*TIRP1* and BD-TIR1-TIR2 can grow on the SD/ − LTHA plates. **b** Split luciferase complementation indicates an interaction between *TIRP1* and TIR1-TIR2_R. **c** Heterodimerization of *TIRP1* and TIR1-TIR2_R detected by Co-IP in *N. benthamiana* leaves; GFP was used as a negative control and actin was used as a loading control. **d** Interaction between nYFP-TIR1-TIR2 and *TIRP1*-cYFP and between nYFP-*GhRVD1* and *TIRP1*-cYFP induced fluorescence in *N. benthamiana* leaves. EV, empty vector. Defense cells are marked by red arrows. **e, f** HR phenotypes associated with co-inoculation of *TIRP1* and plant TNL^TIR^; increasing concentrations of *TIRP1* (OD600 = 0, 0.5, 1.0, and 1.5) gradually alleviated the cell death phenotype induced by TIR1-TIR2; GFP was used as a negative control. Evaluation of protein expression using α-Flag (for TIR1-TIR2, *RPP1_NdA*^TIR^, and *RPS2*) and α-GFP (for *TIRP1*). Immunoblotting of plant actin with α-actin was used as a loading control. **g, h** *TIRP1* outcompetes TIR1-TIR2_R for binding to TIR1-TIR2_R in a concentration-dependent manner in *N. benthamiana* leaves, as determined by coimmunoprecipitation. A series of *Agrobacterium* concentrations of *TIRP1*-GFP are shown (OD600 = 0, 0.5, 1.0, and 1.5) and co-inoculated with a constant concentration of TIR1-TIR2 (OD600 = 1.5) into leaf tissues; GFP was used as a negative control. Blotting of IP groups from different lanes were measured by ImageJ, and the ratio between the maximum values (relative intensity) is represented in the histogram. Three independent experiments were analyzed, and between-group comparisons were made using Student’s *t* test

We next used point-to-point experiments to confirm that *TIRP1* interacted with TIR1-TIR2. The Y2H assay revealed a strong interaction between *TIRP1* and TIR1-TIR2 (Fig. 6a). This interaction, however, did not occur between *TIRP1* and the other TIR proteins (L6, RPP1_NdA and RPS4) which is the same as TIR1-TIR2 in autoactivity and self-association [1, 36, 37] (Fig. 6a). Subsequently, LCA, Co-IP, and bimolecular fluorescence complementation (BiFC) assays consistently indicated an interaction between *TIRP1* and TIR1-TIR2 (or *GhRVD1*) (Fig. 6b–d). In BiFC assays, specific YFP fluorescence was detected exclusively in the plasma membrane of *Nicotiana benthamiana* cells (Fig. 6d), which was consistent with the subcellular localization of *TIRP1* or TIR1-TIR2 (Additional file 1: Fig. S6c). This fluorescence induced by interaction compound was more intensive in defense cells compared with individual YFP fusion proteins. Our results confirm that *TIRP1* interacted with TIR1-TIR2 in vivo on the plasma membrane.

### *TIRP1* inhibits autoactivity and self-association of TIR1-TIR2 by competing for binding to it

To determine the effects of the interaction between TIR1-TIR2 and *TIRP1* on TIR1-TIR2 autoactivity, we co-inoculated TIR1-TIR2 with different concentrations of *TIRP1* in tobacco leaves. As concentrations of *TIRP1* increased, the cell death phenotype induced by TIR1-TIR2 gradually weakened (Fig. 6e and Additional file 1: Fig. S7e), which was further measured by Luciferase reporter (Additional file 1: Fig. S9c). NLR immune receptors have been classified into TNL and coiled-coil (CC) NLRs (CNL). The TIR domain of *RPP1_NdA*, TNL immune receptors from *A. thaliana*, which can trigger cell death when ectopically expressed in *Nicotiana* species [53]. Cell death induced by *AtRPS2*, which encodes a *CNL*, requires different signaling components to mount the ETI response than TNLs [50, 53, 57]. To test whether *TIRP1* also inhibits the typical NLR immune receptors, the *RPP1_NdA*^TIR^ and *AtRPS2* were taken to co-inoculate with different concentrations of *TIRP1* in tobacco leaves*.* Interestingly, increasing concentrations of *TIRP1* was not effective in reducing cell death triggered by *RPP1_NdA*^TIR^ and *RPS2* (Fig. 6e,f and Additional file 1: Fig. S7e), which was further measured by Luciferase reporter (Additional file 1: Fig. S9c), indicating that inhibition of NLR autoactivity by *TIRP1* is not universal in plants. Collectively, our results suggest that *TIRP1* affects the autoactivity of TIR1-TIR2 when co-expressed.

**Fig. 7:**
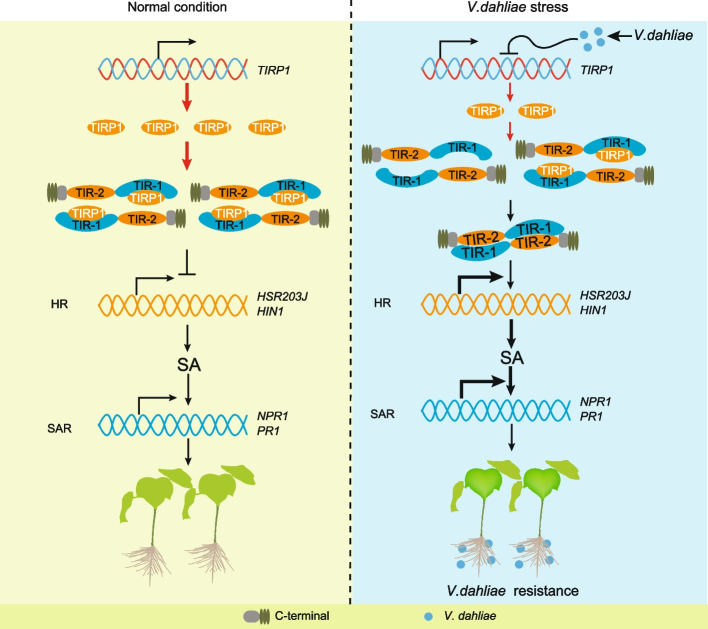
A working model for *TIRP1* regulating self-association, autoactivity, and VW resistance of dual-TIR domain protein in the plant. Under normal growth conditions, the constitutive expression of *TIRP1* mediates accumulation of sufficient protein to competitively bind to TIR1-TIR2, thus inhibiting their self-association, and subsequent immune response mediated by GhRVD1. The downregulation of *TIRP1* expression after *V. dahliae* invasion releases the TIRP1 from TIR1-TIR2 domain of GhRVD1 and indirectly leads to increased TIR1-TIR2 self-association. Furthermore, increasing dimer of TIR1-TIR2 contributes to activate the transcription of hypersensitive response-related gene *HSR203J*/*HIN1* and systemic acquired resistance-related gene *NPR1*/*PR1*, thus enhancing cotton resistance against *V. dahliae.* The width of the arrows indicates the strength of the downstream signaling. Blue circles represent molecules associated with *V. dahliae* infection

Co-IP assays were used to determine whether the self-association of TIR1-TIR2 is affected by interactions with *TIRP1*. In the input group, increasing concentrations of TIRP1-GFP were detected in precipitate from the leaves that co-expressed *TIRP1*-GFP, TIR1-TIR2-Myc, and TIR1-TIR2-HA (Fig. 6g). In the IP group, increasing concentrations of *TIRP1*-GFP weakened TIR1-TIR2 self-association, and additional interactions between *TIRP1*-GFP and TIR1-TIR2 were detected. GFP was used as a negative control with no effect on TIR1-TIR2 self-association (Fig. 6g, h). Our results support a competition model of *TIRP1* and TIR1-TIR2, which predicts that *TIRP1* antagonizes the function of TIR1-TIR2 when regulating cell death (Fig. 7).

### *GhTIRP1* negatively regulates cotton resistance to *V. dahliae* by inhibiting *GhRVD1* oligomerization

In VW-resistant accession ZZ2 from Hap. B, a significant downregulation expression of *TIRP1* was observed in both leaves and roots at 12 and 24 h post-inoculation (hpi) with *V. dahliae* compared with the control (Additional file 1: Fig. S9d). We then used VIGS to knockdown the expression of *GhRVD1*, *TIRP1*, and *GhRVD1-TIRP1* in ZZ2. As previously observed, knockdown of *GhRVD1* expression resulted in typical symptoms of VW (Additional file 1: Fig. S9e). However, TRV::*TIRP1* plants showed enhanced resistance to *V. dahliae* colonization (Additional file 1: Fig. S9e). Interestingly, when the expression of *GhRVD1* was transiently suppressed in the background of TRV::*TIRP1*, VW resistance attenuated and presented with symptoms of disease susceptibility consistent with the *GhRVD1*-silenced plants. The expression of target genes was significantly reduced at the transcript level, and knockdown of *GhTIRP1* expression did not affect the transcriptional activity of *GhRVD1* (Additional file 1: Fig. S9f). These results indicate that *GhTIRP1* negatively regulates cotton resistance to *V. dahliae* and is highly dependent on the normal expression of *GhRVD1* which could result from the inhibition of *GhRVD1* oligomerization by *TIRP1*.

## Discussion

We performed GWAS on VW resistance based on a large-scale upland cotton germplasm resource, and 10 loci were identified. Among them, *SHZDI1/SHZDP2/AYDP1* was introgressed from *G. arboreum*, which likely derived from triple hybridization (*G. thurberi* × *G. arboreum* × *G. hirsutum*). Note that in a core panel of 419 accessions, only 36 accessions carried these introgressed fragments, indicating that this valuable resource has not been widely used in cotton breeding. We know that upland cotton was domesticated from Mexico, prior to further domestication and improvement in the USA; this history is evident in the narrow genetic background and relatively low genetic diversity of this crop species [58]. Distant hybridization breeding between diploid cotton and tetraploid cotton is understood as an effective means to increase the genetic diversity of upland cotton. Our successful identification of *SHZDI1*/*SHZDP2*/*AYDP1* offers an example for how an introgressed fragment has contributed to major improvements in VW resistance in upland cotton. The locus *SHZDI1*/*SHZDP2*/*AYDP1* could be developed into a molecular marker that would allow breeders to directly select this locus when seeking to improve the VW resistance trait in elite lines.

Compared with marker-assisted breeding, biological breeding is a new trend for major crops, and uncovering key genes underlying important traits is crucial for this new breeding approach. Here, we found a large *TIR-NBS-LRR* gene cluster located on *SHZDI1/SHZDP2/AYDP1*, of which multiple genes could have mediated the introgressed fragments with additional VW resistance in *G. hirsutum*. A previously identified QTL, *QVW-A10* in *G. hirsutum*, partially coincided with this introgression segment and *GhDSC1* is likely the candidate gene for resistance against *V. dahliae* [13, 59]. Based on the homologous alignment, *GhDSC1* was a homolog of *GA10G323100* from introgressed fragments, including the TIR domain and NBS domain. Differential expression after inoculation and autoactivity of *GA10G323100* were not detected in Hap. B.

*GhRVD1* with a novel dual-TIR domain which contributed most to VW resistance was used for in-depth functional validation. We confirmed the important contribution of *GhRVD1* to plant VW resistance. Considering polymorphism and introgression at this locus, there could have been longstanding coevolution between *GhRVD1* and *Verticillium* spp. [45]. Additionally, overexpression of *GhRVD1* in cotton resulted in a constitutive defense activation and further enhanced resistance to *V. dahliae*. In plants, NLR-mediated immune responses are often tightly regulated and driven by the nucleotide-bound status of the central NBS domain [54, 60, 61]. Excessive accumulation of *GhRVD1* appears to result in an unequal equilibrium between negative regulators (e.g., *TIRP1*) and *GhRVD1*. Moreover, the negative correlation between the size of the *snc1* mutant and the degree of immunity makes it an important immune-related screening material [62], which could be an additional application for 35S::*GhRVD1* cotton. In the background of cotton, overexpression of *GhRVD1_R* or *GhRVD1_S* can both enhance resistance to *V. dahliae*, while *GhRVD1_S-*mediated resistance is completely abolished in *A. thaliana*, which is attributed to a potential specific negative regulation. The newly identified *GhRVD1* uncovers important strategies for biological breeding. Importantly, overexpression of *GhRVD1_R* not only conferred VW resistance to transgenic cotton, but it also affected the plant architecture. In Xinjiang—the largest cotton production area in the world, both dwarfism and VW resistance germplasm resources are urgently needed to improve crops [63]. The overexpression lines we created address these issues and are expected to contribute to the cultivation of novel cotton varieties.

The N-terminal TIR domain of TNLs was identified as the region mediating downstream signaling, efforts to define the molecular events associated with cell death induction by TNL initially focused on the N-terminal region [2, 36, 37, 52]. We are the first report on identifying a functional TNL protein with a dual-TIR domain on the N-terminal, since no studies have previously assessed its biological significance. Unlike TNLs with a single TIR domain [36, 37], neither TIR1 nor TIR2 could induce cell death on their own, and the minimal autoactive sequence was TIR1-TIR2, indicating that both TIR1 and TIR2 mediated downstream signaling. This result was further supported when co-inoculations of TIR1L-YFP and TIR2L-YFP induced PCD reactions.

Phyre2 uses advanced remote homology detection methods to build 3D models [64], which are considered a suite of tools available in many studies [65]. We used Phyre2 at 90% accuracy to build the model for TIR1-TIR2, which was based on a template with the best-aligned 3D structures. Actually, four out of the five-stranded parallel β-sheet and all five α-helical regions, which are well-characterized in known TIR structures, were also well predicted in TIR1 and TIR2 of GhRVD1. However, without experimental support, there is potentially some uncertainty regarding the accuracy of the predicted model. For example, the absence of the βB-sheet in TIR1 and TIR2 needs further validation through experimental techniques such as X-ray crystallography or cryo-electron microscopy.

Mutations in either the AE or DE interface region disrupt the cell death signaling activity of *SNC1*, *L6*, and *RPS4* TIR domains, and the self-association of *L6* and *RPS4* TIR domain [1]. The autoactivity of TIR1-TIR2, however, only depends on DE interface, which mediates its self-association. This independence in the AE interface is likely due to special pre-existing form of self-association mediated by physical interaction between TIR1 and TIR2. Cell death induced by TIR1L and TIR2L co-inoculation restored dependence on the AE interface and a C-terminal YFP fusion was required to maintain their function, much like some single-TIR domain TNLs [54], which indicates that a dual-TIR domain TNL have an advantage in signal activation.

Considering the formation of TIR1-TIR2 self-association, two possible models initially existed, the first of which is forward arrangement that depends on the physical association between the homeodomains, while only weak self-association was found for TIR1L and was not able to mediate subsequent signaling activity. Consequently, an antiparallel dimer model mediated by physical interaction between one or two groups of TIR1 and the TIR2 domain was supported by experimental results and initiated the downstream signaling pathway.

*TIRP1*, a *FKBP* family protein, co-localizes to the plasma membrane and subsequently inhibits autoactivity and self-association of TIR1-TIR2 by competing for binding to it. Interestingly, *TIRP1* was not effective in reducing cell death triggered by *RPP1_NdA* ^TIR^ and *RPS2*, indicating a specific inhibition induced by physical TIR domain interaction. Moreover, autoactivity inhibition is likely to mediate the negative regulation of *TIRP1* on VW resistance, which is released after *V. dahliae* inoculation or after the knockdown of *TIRP1*. This prediction was further supported by the finding that knockdown of *GhRVD1* abolished the VW resistance of TRV::*TIRP1*. Thus, the *TIRP1–GhRVD1* module provides new insight into how dual-TIR-domain genes confer VW resistance in plants, substantially deepening our understanding of mechanisms used by plants to cope with fungi infection.

## Conclusions

In conclusion, a *TNL* introgressed from *G. arboreum* results in allelic variation in the TIR domain that provides stronger VW resistance and stronger activation of constitutive defense than the native orthologous gene of *G. hirsutum*. The dual-TIR domain on the N-terminal confers intensive autoactivity on *GhRVD1*, which depends on a special homodimerization formation-mediated physical interaction between TIR1 and TIR2 domain. *GhTIRP1* inhibits the autoactivity and self-association of TIR1-TIR2 by competing for binding and negatively regulates cotton resistance to *V. dahliae* by inhibiting GhRVD1 oligomerization (Fig. 7). Our study highlights the role of *GhTIPR1*-*GhRVD1* in cotton VW disease. The resources created for this research can promote genetic approaches to improve VW resistance in upland cotton.

## Methods

### Plant materials and Verticillium wilt resistancephenotyping 

A total of 419 *G. hirsutum* accessions with genetically diverse phenotypic traits were collected from major cotton-growing countries from the China National Gene Bank, Cotton Research Institute, Chinese Academy of Agriculture Sciences, Anyang, Henan Province [66] (Additional file 2: Table S3).

All 419 accessions were planted in the disease nursery at Anyang, Henan, China and Shihezi, Xinjiang, China. *V. dahliae* strains Vd076, Vd080, and Vd991 were inoculated in advance, and the susceptible control (JM11) displayed typical symptoms of Verticillium wilt with susceptible disease indexes of 51.05, while the resistance control (ZZ2) had resistance disease indexes of 20.83.

Field experiments of 419 accessions followed a randomized complete block design with three replicates. Each replicate was seeded in a row of 6 m with 20–30 plants, and cotton plants grew at a distance of 20 cm within each row and 50 cm between rows. The Verticillium wilt disease index (DI) and disease percent (DP) evaluations were conducted according to the Chinese Technical Specification for Evaluating Resistance to Verticillium wilt of Cotton (NY/T 2952–2016) [11].

### Genome-wide association study

The latest high-quality *G. hirsutum* TM-1 genome was used as reference genome (CRI-TM-1, version1.0). Genotypic data with a 6.55-fold-coverage depth sequencing were obtained from a previous study [66]. Clean reads were mapped to the TM-1 genome by BWA, and SNPs were called by GATK3.8 based on a previously reported pipeline [32]. We performed GWAS for VW using 1,944,369 SNPs markers with a minor allele frequency > 0.05, a missing rate < 20% and heterozygous rate < 10%. The Efficient Mixed-Model Association eXpedited (EMMAX) software [27] was used for GWAS. The *P-*value threshold was calculated with a suggested threshold of *P* = 1/*n* (*n* = 198,736, the effective number of independent SNPs) [67], while the significant − log_10_ (*P*) threshold was approximately 5.3. Quantile–quantile (QQ) plots and Manhattan plots were visualized using the method described by Turner [68].

We extracted the SNPs located within 1 M on either side of lead SNP from the genotyping file and calculated the haplotype block using LDBlockShow (version 1.40) and default parameters [69]. High-confidence genes around significant SNPs were used for candidate gene analysis based on ANNOVAR v1.0 with gene annotations [70]. All the nonsynonymous polymorphisms in the candidate region were identified.

### Evolutionary analyses

The exotic introgression analysis followed a previously described procedure [26, 31]. The two potential donors for introgressed fragments, *G. barbadense* and *G. arboreum*, were evaluated in the introgression analysis. Only fragments from *G. arboreum* were identified in *SHZDI1/SHZDP2/AYDP1*, supporting that *SHZDI1* was introduced by *G. arboreum* introgression. A total of 36 samples were identified as accessions carrying the introgression on A10 ranging from 112.7 to 113.75 Mb. The SNP tree method was used to confirm the above result. The SNPs from 215 *G. arboreum* and our GWAS panel were called by GATK 3.8 software followed by our previous report [32]. A total of 7062 SNPs was identified within the introgressed fragment on *SHZDI1/SHZDP2/AYDP1*. SNPs were used to construct phylogenetic tree using FastTree with default parameters [71]. A phylogenetic tree showed that 36 upland cotton accessions carried the *G. arboreum* fragment clustered together with *G. arboreum*. We also extracted the SNPs from *GhRVD1* to construct another phylogenetic tree. The topology of this tree again confirmed that 36 upland cotton accessions carried *G. arboretum* fragments clustered together with *G. arboreum* (Additional file 1: Fig. S6i). The F_*st*_ value between population 1 (Hap. B) and population 2 (Hap. A, C-E) was evaluated by Vcftools [72] with a 50 kb window and a 10 kb step. For collinearity analysis, a fragment of TM-1 on A10 ranging from 112 to 114 Mb were cut into 1 kb fragments to blast with *G. arboreum* genomic sequence to find the syntenic block between the two species. We found the genomic spectrum of *SHZDI1/SHZDP2/AYDP1* (ranges from 112.7 to 113.75 Mb on A10) corresponded to 132.45 to 133.60 Mb on Chr10 of *G. arboreum*.

### Quantitative real-time-PCR

Total RNA was extracted from cotton seedling leaves and *A. thaliana* plants using a RNAprep Pure Plant Kit (Tiangen). cDNA was synthesized using PrimeScript™ RT Reagent Kit with gDNA Eraser (Takara, China), and qRT-PCR analyses were conducted using the SYBR^®^ Premix Ex Taq™ (Takara, China) on an ABI 7900 Real-Time PCR System (Applied Biosystems, USA). We used Primer Premier 6.0 software to design all qRT-PCR primers (Additional file 2: Table S2). Three biological replicates and three technical replicates for each sample were performed. The dissociation curves for each reaction were analyzed, and the 2^−ΔΔCT^ method was used to calculate the relative expression levels of each target gene [11].

### mRNA-seq analysis

Cotton roots were harvested 24 h post-inoculation (hpi) with Vd080 or water for both JM11 (susceptible) and ZZ2 (resistance) plants. mRNA-seq data was used for screening of candidate gene in each locus. Clean reads were obtained by removing reads containing adapters and poly-N, which were low-quality reads from raw read data. The filtered reads were mapped to the *G. hirsutum* reference genome (CRI-TM-1, version1.0) using Hisat2 software [73]. Moreover, roots of ZZ2 plants with an introgressed fragment were further harvested at 9 h and 72 hpi with Vd080 or water and mapped to a *G. arboreum* reference genome. mRNA-seq data was used for screening of candidate gene on *SHZDI1/SHZDP2/AYDP1*.

### *Verticillium dahliae* culture and plant inoculation

The *Verticillium dahliae* strains Vd080 (isolation from Hebei, China), Vd076 (isolation from Henan, China), and Vd991 were obtained from the Institute of Cotton Research of the Chinese Academy of Agricultural Sciences. *V. dahliae* was prepared according to a general protocol [11]. The *V. dahliae* isolates were grown on potato dextrose agar medium at 25 °C for 10 days. The colonies were then inoculated with Czapek’s liquid medium for 4 days of growth at 25 °C with shaking (180 rpm). Cotton seeds were sown in vermiculite and sand (6:4 v/v ratio) and grown at 28 °C with a 16 h/8 h light/dark photoperiod for 3 weeks. Using the root dip method, each plant was inoculated with 10 ml of *V. dahliae* suspension (~ 1 × 10^7^conidia/ml) [11]. Control plants were inoculated with an equal volume of sterile distilled water.

*A. thaliana* sterile seedlings were grown on plates of Murashige and Skoog (MS) medium for 2 weeks, after which 10 μL of conidial suspension (~ 1 × 10^7^conidia/ml) was inoculated on *A. thaliana* plants roots. Control plants in each treatment were inoculated with an equal volume of sterile distilled water. Verticillium wilt phenotypes were investigated 10 days post-inoculation. The disease severity of *A. thaliana* plants was graded from 0 to 5, and the DI was calculated as previously described [74].

### Vector construction

All vectors were constructed using a one-step cloning method. A pair of primers with double homologous arms and double restriction sites were used to amplify fragment by Phanta Super-Fidelity DNA Polymerase (Vazyme, China), which was then ligated to the vector with the same homologous arms and restriction sites by a Mut Express II Fast Mutagenesis Kit V2 (Vazyme, China). All point mutation sequences and fusion sequences were produced by PCR-driven overlap extension [75] and double mutation sequences were produced by PCR-driven overlap extension based on a single mutant template. All point mutations and fusion sequences were first cloned into pENTR/D-TOPO, and other vectors involved in mutation experiments were amplified from corresponding mutant pENTR/D-TOPO vectors. Except for NH188189AA mutants of TIR1L, other mutant vectors were constructed with the same primers of wild-type construction but with different vector templates.

### Virus-induced gene silencing (VIGS) in upland cotton

For the VIGS assays, 16 fragments (200–300 bp) amplified from introgressed materials (ZZ2) cDNA were integrated into the vector pYL156 (TRV) to construct TRV::CG01/CG02/CG03/CG04 /CG05/CG06/CG07/CG08/CG09/CG10/CG11/CG12/CG13/CG14/*TIRP1* and introduced into the *Agrobacterium tumefaciens* strain GV3101. The primer pairs are shown in Additional file 2: Table S2. *Agrobacterium* strains harboring pYL156 plasmids combined with strains harboring pYL192 vectors were mixed in a 1:1 ratio and co-infiltrated into the cotyledons of 2-week-old cotton plants (ZZ2); TRV::*CLA1* (*cloroplastos alterados 1* gene) was used as a positive control [76]. We cloned silence fragments of *GhTIRP1* and *GhRVD1* with corresponding specific primers (Additional file 2: Table S2), which were inserted into pYL156 to form the TRV::*GhRVD1-TIRP1* vector using the PCR-driven overlap extension method [75, 77]. One week later, the leaves of three plants were harvested for RNA extraction and qRT-PCR analyses to identify target gene expression. Each plant was subsequently inoculated with 10 ml of *V. dahliae* suspension (~ 1 × 10^7^conidia/ml) or water using the root dip method [11]. VW phenotypes were investigated 9 days post-inoculation, and it was fully developed 3 weeks post-inoculation.

### *A. thaliana *transformation and evaluation of Verticillium wilt resistance 

*A. thaliana* seeds were sown on compost and vernalized for 4 days (dark, 4 °C). Seedlings were grown under controlled conditions: 21–23 °C; 10 h light/14 h dark; 75% humidity. The pCambia2300 vector under a control of 35S promoter was used to generate the overexpressed transgenic lines. The *GhRVD1_R* and *GhRVD1_S* sequences were amplified from the cDNA of ZZ2 and ZM24 using the same primer pairs. The *Agrobacterium* strain GV3101 containing the *GhRVD1_R* and *GhRVD1_S* overexpression vector was used to transform the ecotype Col-0 of *A. thaliana* by the floral dip method [78]. T_0_ transgenic seeds were obtained and selected on the MS medium containing kanamycin (50 μg/mL). OE-*GhRVD1_R* and OE-*GhRVD1_S* T_2_ single-copy lines were selected to study the population of VW phenotypes.

### Chemical staining

DAB staining, leaves were vacuum-infiltrated for 30 min with 1 mg/mL DAB solution, placed in the dark for 16 h, and then destained in 90% ethanol before imaging. For cell death assays, leaves were soaked in trypan blue dye (1 g phenol, 1 mg trypan blue, 1 ml lactic acid, and 1 ml glycerol dissolved in 1 ml sterile distilled water) and then stained by boiling. After cooling to room temperature, samples were decolorized with a chloral hydrate solution (2.5 g/ml).

### Measurements of free salicylic acid

The free SA content was determined via LC–MS (Agilent 1260 Infinity-Agilent 6420A) (Nanjing, China) as previously described [79].

### Observation of autoactivation responses by transient expression

Constructs for transient expression were prepared using the primers listed in Additional file 2: Table S2. In general, sequences were first restriction-cloned into pENTR/D-TOPO vectors (QBV3) or modified pENTR/D-TOPO with a 3′ sequences encoding 3 × Flag epitope tag (QBV3C) [80, 81]. Next, these entry clones were recombined into the pEarleygate vector pEG100 or PEG101 (-YFP-HA) using LR Clonase (Invitrogen, Carlsbad, CA) [50]. Finally, the target fusion protein with a 3 × Flag epitope tag (QBV3C to PEG100) and fusion proteins with YFP and HA epitope tag (QBV3 to PEG101) were obtained. All the primers are described in Additional file 2: Table S2.

*Nicotiana benthamiana* were grown in a controlled environment with a 10 h light/14 h dark at 24 °C for 4 weeks. *Agrobacterium tumefaciens* cultures containing pEG vector were grown overnight in LB with the selection at 28 °C on a shaker (OD600 = 1.5). Cells were pelleted by centrifugation at 4000* g* and resuspended in infiltration buffer (10 mM MgCl_2_, 10 mM MES, 150 μM acetosyringone). The cells were diluted to the appropriate OD600 and infiltrated into leaf tissue using a needleless syringe; phenotypes were observed 7 days post-inoculation [82]. Percentage of necrosis area was measured in six biological replicates.

### *Nicotiana benthamiana* leaves viability assays

The firefly luciferase gene (LUC) (clone from pGWB435 Vector) was constructed under a 35S promoter control (pCambia2300). The *Agrobacterium* strain GV3101 containing the Luciferase overexpression vector (OD600 = 1.4) was co-inoculated with cultures containing the pEG vector for transient expression. Fluorescence intensity was captured 2 days post-inoculation using a Tanon 5200 Multi Chemiluminescent Imaging System (Tanon, Shanghai, China). Fluorescence signaling was measured in three independent biological replicates to support the statistical significance (*P* < 0.05). In addition, luciferase activity was evaluated using the luciferase assay system (Promega, USA) following the manufacturer’s protocol.

### Cotton protoplast viability assays

Cotton protoplasts were isolated from 14-day-old plant leaves using a plant protoplast preparation and transformation kit (Real-Times Biotechnology, Beijing) [83]. LUC expressed under the 35S promoter was the same as *N. benthamiana* leaf viability assays. Specific constructs were co-transfected with the LUC plasmid into cotton protoplasts using the polyethylene glycol method [83]. Fluorescence intensity was captured 40 h after transfection using a Tanon 5200 Multi Chemiluminescent Imaging System (Tanon, Shanghai, China). Decreases in luminescence were compared with controls co-transfected with an empty vector (EV). Concentrations of the protoplasts and plasmids used for transformation were maintained at the same level for each experiment. The experiments were repeated at least three times and produced the same results.

### Protein extraction and Western blotting

For transient expression analysis, 2.5 g *N. benthamiana* leaves expressing the indicated proteins were ground into a powder with liquid nitrogen 48 h after inoculation. An equal volume of protein isolation buffer (1 mM EDTA pH 8.0, 20 mM Tris–HCl pH 7.5, 5 mM dithiothreitol, 150 mM NaCl, 0.1% sodium dodecyl sulfate [SDS], 10% glycerol, and 1 × protease inhibitor cocktail) was then added into the powder and allowed to stand for 30 min at 4℃. The mixture was centrifuged at 4℃ for 10 min at 13,000 × *g*. The supernatant was transferred to a new tube and boiled in protein sample buffer (Beijing, Solarbio) for 5 min. Proteins were separated on 10% SDS–polyacrylamide gel electrophoresis (SDS-PAGE) and immunoblotted with different antibodies according to methods used by a previous study [84].

### Coimmunoprecipitation assays (Co-IP)

Constructs for Co-IP were prepared using the primers listed in Additional file 2: Table S2. In general, sequences were cloned into modified pWBHS with 3′ or 5′ sequences encoding 3 × HA, 4 × Myc, and GFP epitope tags using a one-step cloning method. Protein extraction was performed using the procedure described above. Target proteins were immunoprecipitated by protein A/G beads (TransGen) combined with different antibodies (MBL, Japan). Beads were washed three times with lysis buffer and boiled in protein sample buffer for 5 min [84]. Separation and immunoblot were performed using the methods described above.

### Yeast two-hybrid assays (Y2H)

Y2H assays were performed using the GAL4-based two-hybrid system, and sequences were cloned into pGADT7 and pGBKT7 to generate pGAD-Preys and pGBD-Baits. Corresponding primers are listed in Additional file 2: Table S2. pGAD-Preys and pGBD-Baits Fusion proteins containing YFP were constructed with PEG101 (-YFP-HA) vector templates.pGBD-Baits and pGAD-Preys were transformed into Y2HGold. Transformed cells were grown on a synthetically defined (SD) medium lacking Leu or Trp (SD/-LT) for 4 days. Target sequences were then detected, and the yeast cells were screened on an SD medium lacking Leu, Trp, Ade, and His (SD/-LHTA). The yeast transformation and growth assays were performed as described in the Matchmaker Gold Yeast Two-Hybrid System User Manual (Takara).

### Luciferase complementation assays (LCA)

Sequences were cloned into pCAMBIA1300-nLUC and pCAMBIA1300-cLUC, and corresponding primers are listed in Additional file 2: Table S2. The *Agrobacterium* strain GV3101 containing nLUC or cLUC recombinant vectors was co-infiltrated into *N. benthamiana* leaves. The empty nLUC and cLUC recombinant vectors were used as negative controls. Fluorescence intensity was captured and measured using the aforementioned method.

### Systematic Y2H library screening by matingand sequencing 

Library construction and mating were performed as described in the Matchmaker Gold Yeast Two-Hybrid System User Manual (Takara). In general, library cDNA of *Gossypium hirsutum* roots (Hap. E) was constructed into pGADT7 to generate the pGAD-Preys library. Subsequently, library plasmids were transformed into Y187 yeast strains to obtain a secondary yeast library. The constructed bait vector (pGBKT7-TIR1-TIR2) was transformed into AH109 yeast strain and was tested for autoactivity by growth SD plates depleted of tryptophan, adenine, and histidine (SD-Trp/Ade/His).

Yeast strains AH109 containing pGBKT7-TIR1-TIR2 were mated with a library of the Y187 yeast strains. Mating was performed according to the manufacturer’s instructions (Takara). Diploids carrying both plasmids and dimerization of pGAD-Preys and pGBD-Baits were tested by growth on selection media (SD-Trp/Leu/Ade/His) [85]. Colonies from SD-Trp/Leu/Ade/His plates were used for prey identification by Sanger sequencing after colony PCR using T7 and 3′ AD primers.

### Subcellular localization assays

Sequences were cloned into pCAMBIA2300-GFP and transformed into *A. tumefaciens* GV3101 [86]. Strains containing recombinant vectors were transiently expressed in 4-week-old *N. benthamiana*, and green fluorescence was captured 40 h after transfection using an LSM780 confocal microscope.

### *V. dahliae* recovery assays

To further evaluate resistance to VW, *V. dahliae* from the first stem nodes of TRV::*00*, TRV::*GhRVD1*, TRV::*TIRP1*, and TRV::*GhRVD1-TIRP1* plants were isolated. Stem segments (4.5 cm) were surface disinfested for 5 min in 5% NaClO and then sliced into five parts. The stem fragments were placed on potato dextrose agar (PDA) plates and incubated at 25 °C for 6 days.

### Statistics 

All presented *P* values correspond to two-sided *P* values using Student’s *t* test. One-way ANOVA was used in the statistical analysis, and means labeled with different letters indicate significant difference at *α* = 0.05. Differential expression analysis between two groups was performed using the EdgeR.

## Supplementary Information

**Additional file 1: Figure S1.** Essential information of GWAS and phenotypic variation in cotton. **Figure S2.** Introgression analysis on *SHZDI1/SHZDP2/AYDP1* and haplotype blocks and candidate genes expression of located QTLs from SHZDP and AYDP. **Figure S3.** Haplotype blocks and candidate genes expression of located QTLs from SHZDI and AYDI. **Figure S4.** Screening candidate genes of *SHZDI1/SHZDP2/AYDP1*. **Figure S5.** Alignment of TNL^TIR^ and GhRVD1 overexpression A. thaliana lines. **Figure S6.** Phylogenetic tree of TNL^TIR^, 3D structures of TIR1-TIR2 and functional verification of *GhRVD1*. **Figure S7.** Statistical of HR necrosis area. **Figure S8.** Trypan blue staining of HR necrosis area. **Figure S9.** GhTIRP1 inhibiting acquired *V. dahliae* resistance from GhRVD1.

**Additional file 2: Table S1.** Details of lead SNP in each locus. **Table S2.** Primer sequences used in this study. **Table S3.** Verticillium wilt phenotyping data of a core collection of upland cotton used in GWAS. **Table S4.** Gene annotation of candidate genes locating in the introgressed fragment. **Table S5.** The conserved domain information of TIR-NBS-LRR genes. **Table S6.** Gene expression profile of an introgressed line under 9- and 72-hour post inoculation of V. dahliae. R1, R2 and R3 represent the three biological replicates. **Table S7.** Result of systematic Y2H library screening sequencing. **Table S8.** Exotic introgression analysis of *G. hirsutum *on chromosome A10.

**Additional file 3.** Uncropped western blot images related to Figs. 3, 4, 5 and 6 and Additional file 1: Fig. S5.

**Additional file 4.** Review history.

## Data Availability

The raw mRNA-seq data from this study are deposited in the NCBI SRA under PRJNA953671 [87]. The published sequencing data for the *G. arboreum* were downloaded from NCBI SRA database under PRJNA349094 [88]. The published sequencing data used for GWAS in *G. hirsutum* were downloaded from NCBI database under PRJNA399050 [89]. The published sequencing data for the *G. barbadense* were downloaded from NCBI database under project PRJNA414461 [90]. The genome sequences for *G. hirsutum* and *G. arboreum* were downloaded from GRAND database [91].
