# Supplementary material for: Genome-wide association analysis reveals a novel pathway mediated by a dual-TIR domain protein for pathogen resistance in cotton
Source: Genome Biol. 2023 May 10;24:111. doi: 10.1186/s13059-023-02950-9 (PMC10170703; doi:10.1186/s13059-023-02950-9)
Supplement: Supplementary file 1 — Additional file 1: Figure S1. Essential information of GWAS and phenotypic variation in cotton. Figure S2. Introgression analysis on SHZDI1/SHZDP2/AYDP1 and haplotype blocks and candidate genes expression of located QTLs from SHZDP and AYDP. Figure S3. Haplotype blocks and candidate genes expression of located QTLs from SHZDI and AYDI. Figure S4. Screening candidate genes of SHZDI1/SHZDP2/AYDP1. Figure S5. Alignment of TNLTIR and GhRVD1 overexpression A. thaliana lines. Figure S6. Phylogenetic tree of TNLTIR, 3D structures of TIR1-TIR2 and functional verification of GhRVD1. Figure S7. Statistical of HR necrosis area. Figure S8. Trypan blue staining of HR necrosis area. Figure S9. GhTIRP1 inhibiting acquired V. dahliae resistance from GhRVD1. [file 13059_2023_2950_MOESM1_ESM.docx]

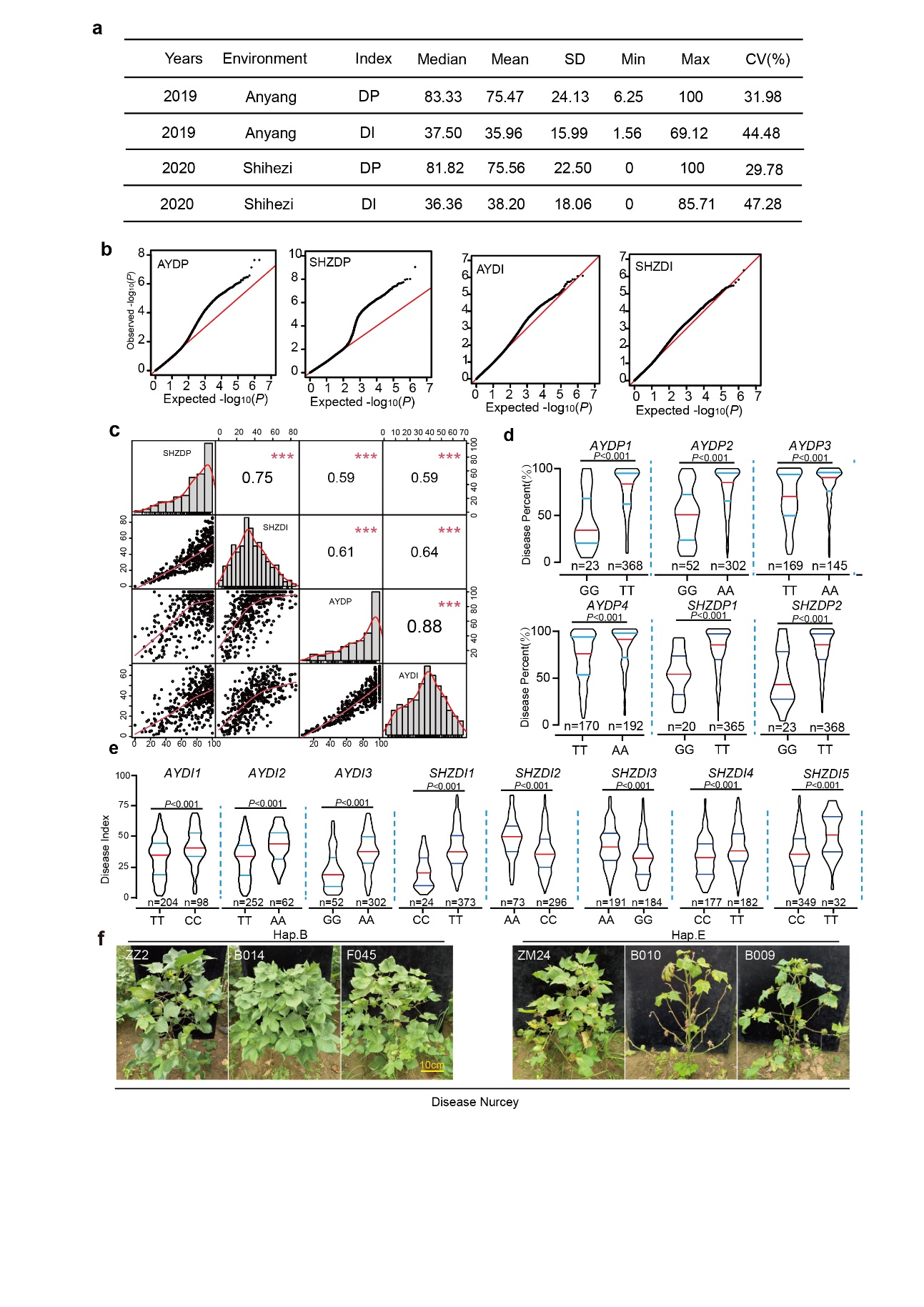


**Fig. S1 Essential information of GWAS and phenotypic variation in cotton. a**. Verticillium wilt disease index (DI), and disease percentage (DP) of accessions in the disease nursery at Anyang and Shihezi. SD, standard deviation; CV (%), coefficient of variation. **b.** Quantile–quantile plot for AYDP, SHZDP, AYDI and SHZDI. **c,** Correlation analysis of Verticillium wilt resistance in different conditions. *** indicates extremely significant difference at *P* = 0.001. **d-e**, DP of different alleles at the peaks for *AYDP1*, *AYDP2*, *AYDP3*, *AYDP4*, *SHZDP1* and *SHZDP2*. DI of different alleles at the peaks for *AYDI1*, *AYDI2*, *AYDI3, SHZDI1, SHZDI2, SHZDI3, SHZDI4* and *SHZDI5* (Student’s t-test). Alleles were marked on the abscissa, the median was marked on the red horizontal line, and the number of materials was marked above the abscissa. **f,** Plant wilt phenotype of *G. hirsutum* accessions carrying Hap. B (introgressed fragments) and Hap. E in disease nursery.


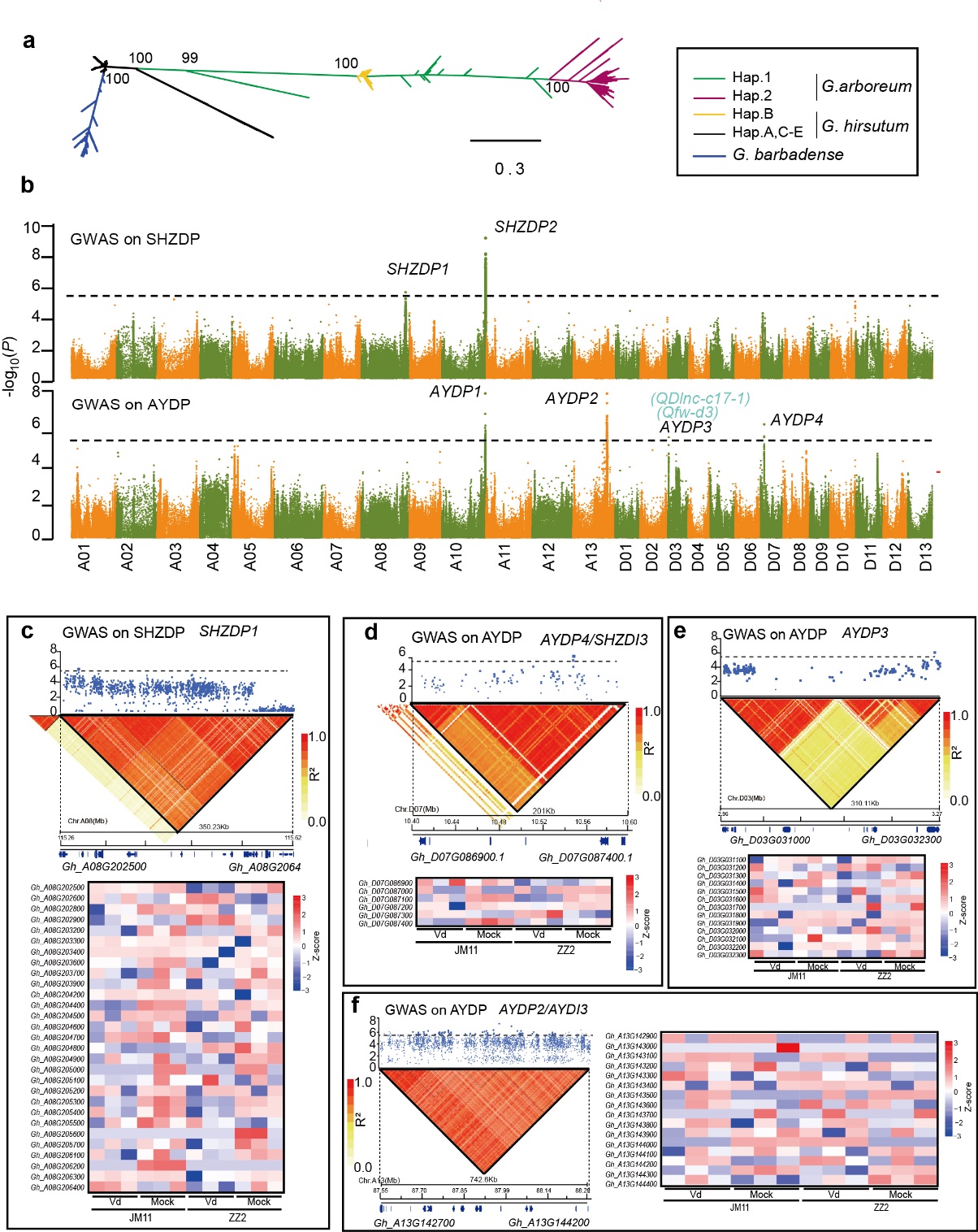


**Fig. S2** **Introgression analysis on *SHZDI1*/*SHZDP2*/*AYDP1* and haplotype blocks and candidate genes expression of located QTLs from SHZDP and AYDP.**

**a,** SNPs located in the putatively introgressed genomic region were used to construct the SNP-based phylogenetic tree composed of upland cotton, *G.barbadense* and *G.arboreum*. **b**, Manhattan plot for SHZDP and AYDP, High-confidence QTLs are marked in black and blue words.**c-f**, haplotype blocks around *SHZDP1, AYDP2, AYDP3* and *AYDP4* was estimated using pairwise LD correlations (R^2^); mRNA-seq data of CGs from different QTLs at 24 hours post inoculation with Vd080 in the roots of ZZ2 (resistant) and JM11 (susceptible), Z-score of FPKM was indicated on the heat map.


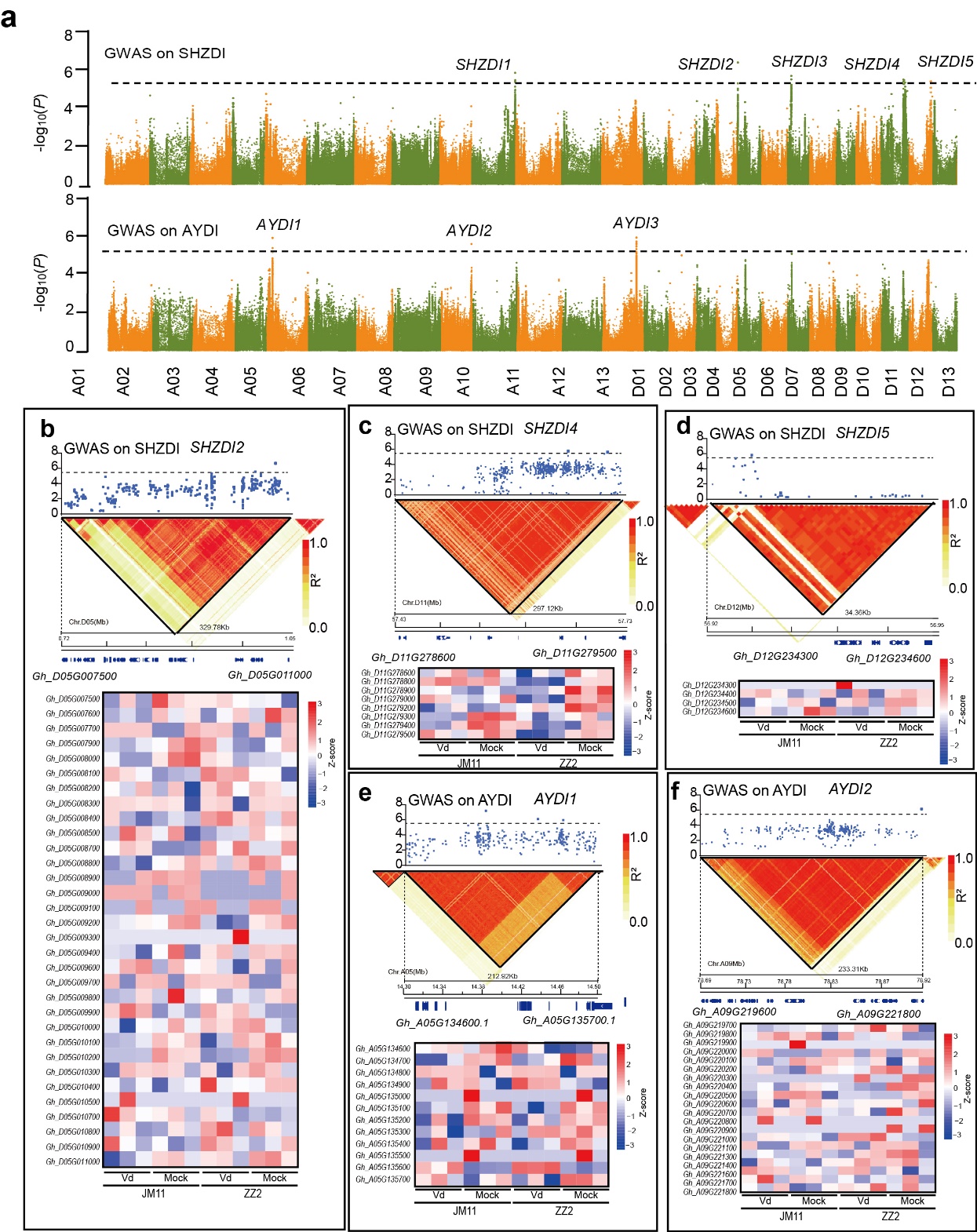


**Fig. S3 Haplotype blocks and candidate gene expression of located QTLs from SHZDI and AYDI.**

**a**, Manhattan plot for SHZDI and AYDI, High-confidence QTLs are marked in black and red words. **b-f**, haplotype blocks around *SHZDI2, SHZDI4, SHZDI5, AYDI1* and *AYDI2* was estimated using pairwise LD correlations (R^2^); mRNA-seq data of CGs from different QTLs at 24 hours post inoculation with Vd080 in the roots of ZZ2 (resistant) and JM11 (susceptible), Z-score of FPKM was indicated on the heat map.


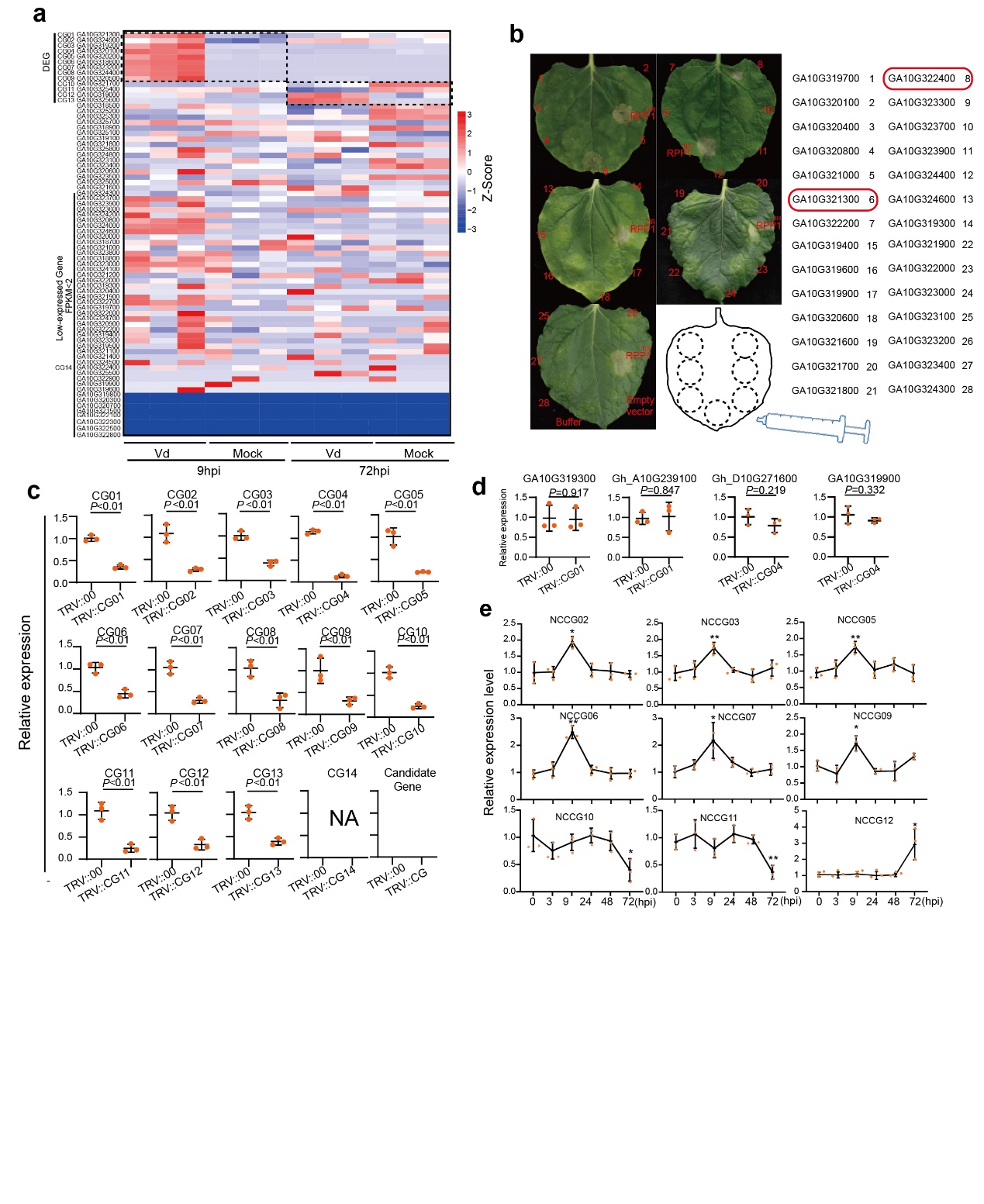


**Fig. S4 Screening candidate genes of *SHZDI1/SHZDP2/AYDP1*. a,** mRNA-seq data of introgressed fragments genes in introgressed Hap. B (ZZ2). FPKM was indicated on the heat map (row normalization was performed). Different expression of candidate genes (CG01-CG13) at 9 or 72hpi with Vd080 in the roots; water was used for the mock inoculation. Three replicates are represented. **b,** HR phenotypes associated with TIR-domain from 28 TNLs as well as the positive (*RPP1_NdA*^TIR^) and negative control (empty vector or buffer). **c**, Expression of 14 target genes was significantly reduced at the transcript level. Significant differences were measured between TRV::*00* and TRV::CG (Student’s t-test). **d**, Expression of potential off-target sites in silencing cotton plant. **e**, Expression patterns of 9 of the14 candidate genes at 0, 3, 9, 24, 48 and 72 hpi with *V. dahliae* strain Vd080 in introgressed Hap. B (ZZ2), measured by qRT-PCR. Significance level *P* < 0.05 is represented by *, and *P* < 0.01 is represented by ** (Student’s t-test).


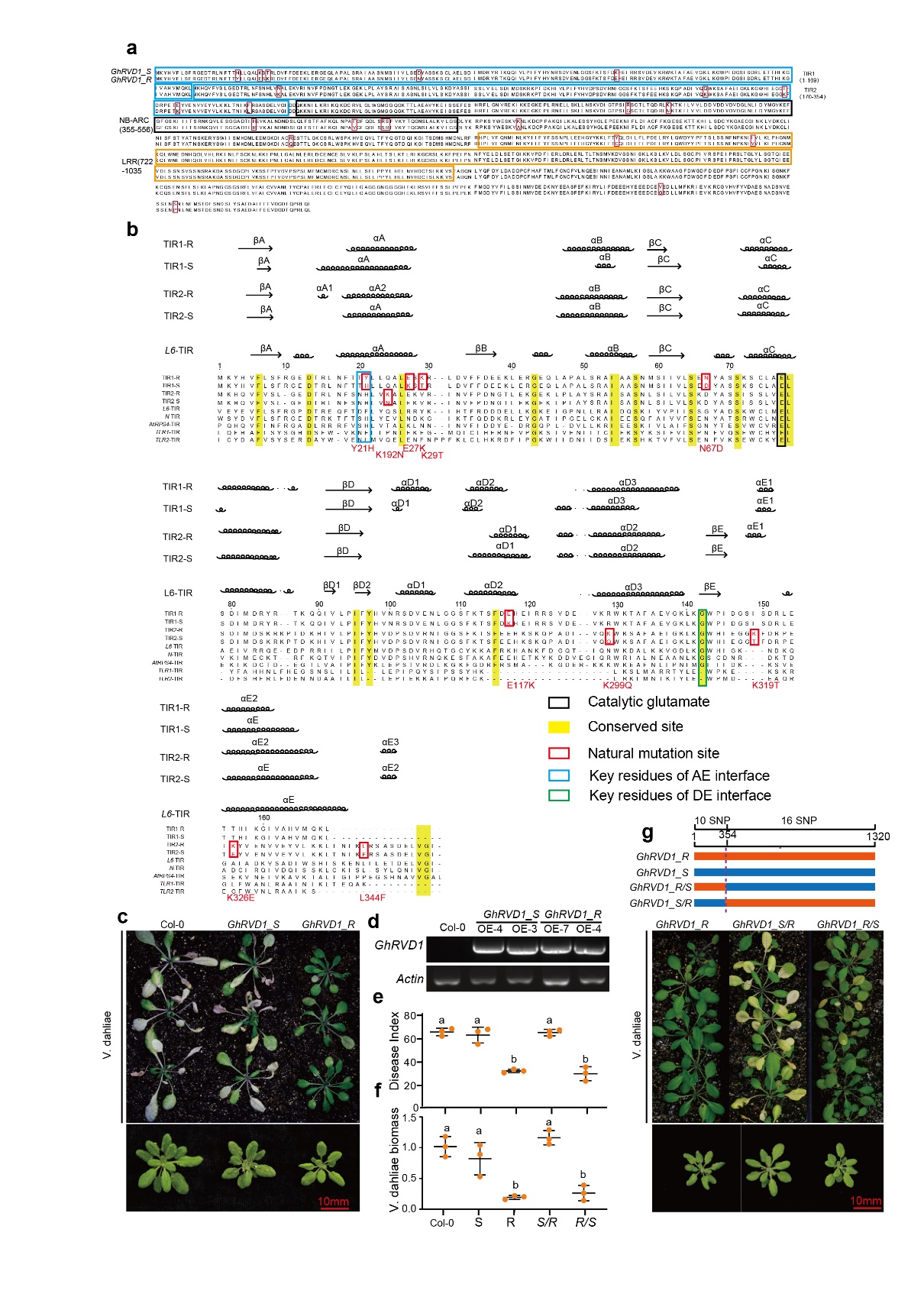


**Fig. S5 Alignment of *TNL*^TIR^ and *GhRVD1* overexpression *Arabidopsis thaliana* lines.** **a.** Two types of *GhRVD1* alleles were detected with 22 nonsynonymous mutations in the amino acid sequences. Mutations are displayed by a red box. **b.** Multiple sequence alignment of TIR domains. Amino acid sequences from the TIR domains of *L6* (residues 59–240), *N* (10–191), and *RPS4* (15–191) were aligned with the sequences of the TIR domains using MUSCLE. Phyre2 was used to build the model for TIR1-TIR2, which was based on a template with the best aligned 3D structures: *NbRoq1* (7JLX) and *VrRPV1* (5KU7). The positions of the secondary structure elements in TIR1, TIR2, and *L6* are shown at the top, center and bottom, respectively. **c**, VW phenotype of *GhRVD1_R* and *GhRVD1_S* overexpression *A.thaliana* lines (OE-3 of 35S::*GhRVD1_S* and OE-7 of 35S::*GhRVD1_R*) in the background of ecotype Col-0. **d**, Exogenous transcripts detected in both genotypes using semi-quantitative PCR. **e-f**, DI of overexpression *A. thaliana* carrying different genotypes inoculated with *V. dahliae*. Different letters indicate significant difference at α=0.05 level via One-way ANOVA analysis. Fungal biomass was measured using qRT-PCR. **g**, Overexpression of two artificial constructs, *GhRVD1_R/S* and *GhRVD1_S/R*. Schematic diagram of artificial gene construction (top) and overexpression *A. thaliana* carrying different genotypes inoculated with *V. dahliae* (bottom)*. GhRVD1_R/S* indicats a construction by recombination of TIR1-TIR2-R into *GhRVD1_S* background and *GhRVD1_S/R* indicats a construction by recombination of TIR1-TIR2-S into *GhRVD1_R* background.


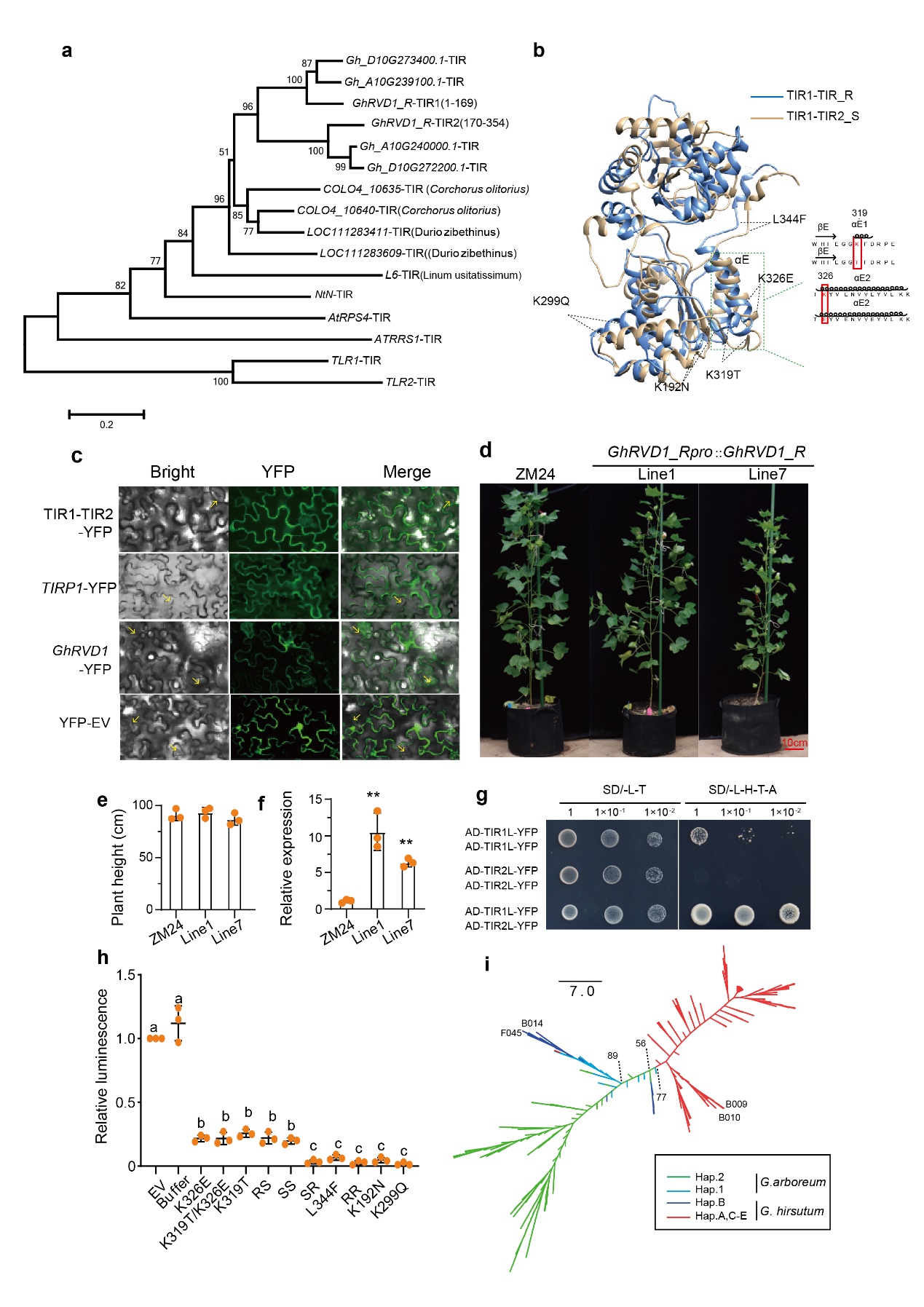


**Fig. S6 Phylogenetic tree of TNL^TIR^, 3D structures of TIR1-TIR2 and** **functional verification of *GhRVD1*. a,** Phylogenetic tree of TIR1, TIR2 and other homologous sequence from *G. hirsutum,* Malvaceae (*Corchorus olitorius*, *Durio zibethinu*s and *Linum usitatissimum*), *Nicotiana tabacum*, and *A. thaliana*. TIR domains from TLR1 and TLR2 were used as outgroup. **b**, Predicted 3D structure of the TIR1-TIR2_R and TIR1-TIR2_S derived from homology modeling. Allelic variations and differences in the secondary structure in α-helix E are shown with black residues and green squares, respectively. The positions of the secondary structure elements are shown on the right. **c**, Fluorescence microscopy images (48 h post-infiltration) of the localization of the TIR1-TIR2-YFP, *GhRVD1*-YFP, and *TIRP1*-YFP in transiently transformed *N. benthamiana* leaf epidermal cells; YFP was used as control. **d**, *GhRVD1_R* under the native *GhRVD1_R* promoter. **e-f**, Plant height and expression of *GhRVD1*. Significance level *P*< 0.01 is represented by **. **g**, Growth of yeast cells co-expressing AD and BD fusions on synthetic media. **h**, Luciferase avtivity was detected using luciferase assay system (Promega, USA) 2 days post-inoculation. Different letters indicate significant difference at α=0.05 level via One-way ANOVA analysis. **i**, SNPs from *GhRVD1* were used to construct the SNP-based phylogenetic tree composed of upland cotton and *G.arboreum*.


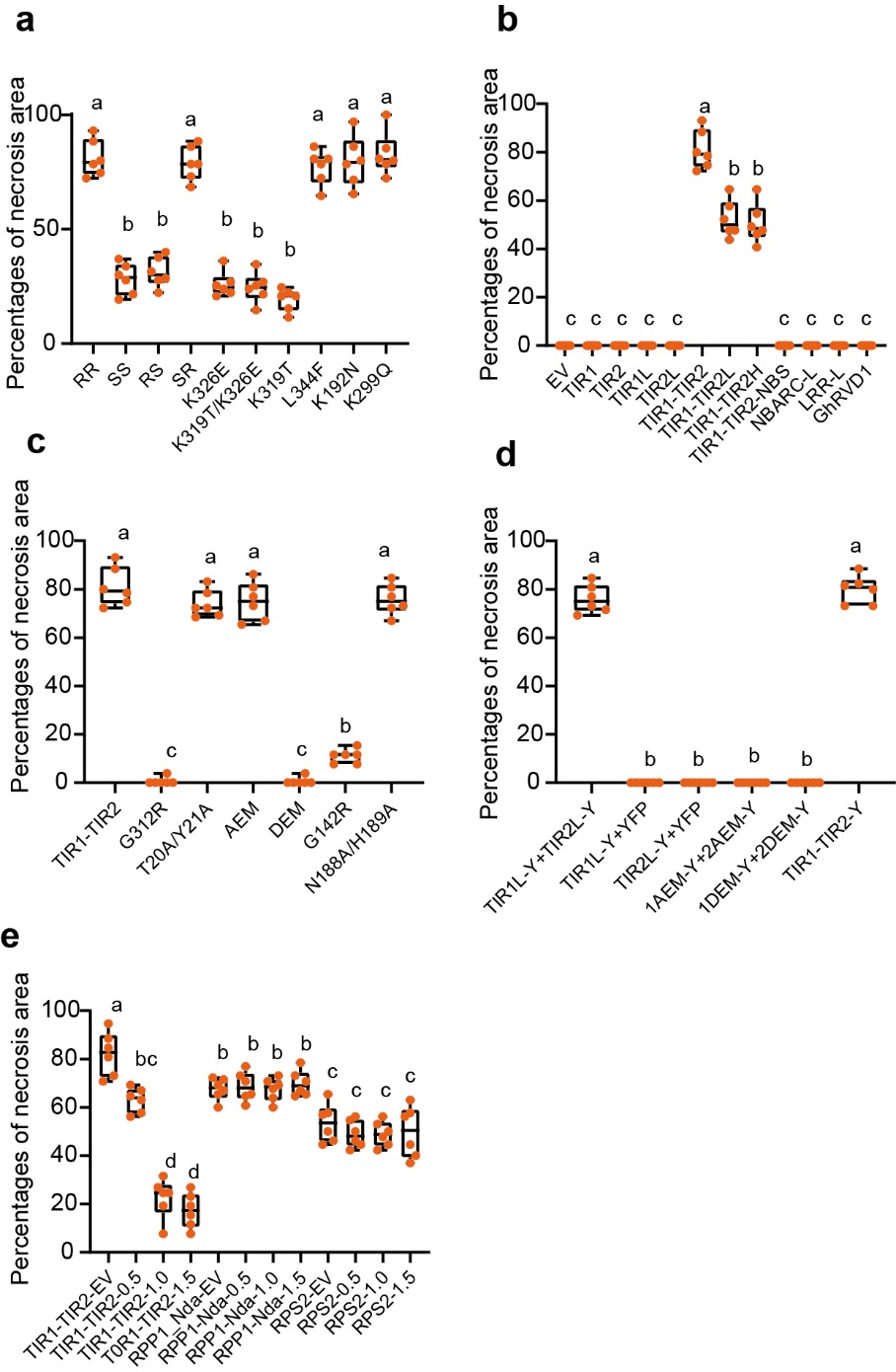


**Fig. S7 Statistical of HR necrosis.** The percentage of necrosis area accounting for the infiltration area for Fig. 4b (**a**), Fig. 4i (**b**), Fig. 5a (**c**), Fig. 5f (**d**) and Fig. 6e (**e**). Different letters indicate significant difference at α=0.05 level using One-way ANOVA analysis.


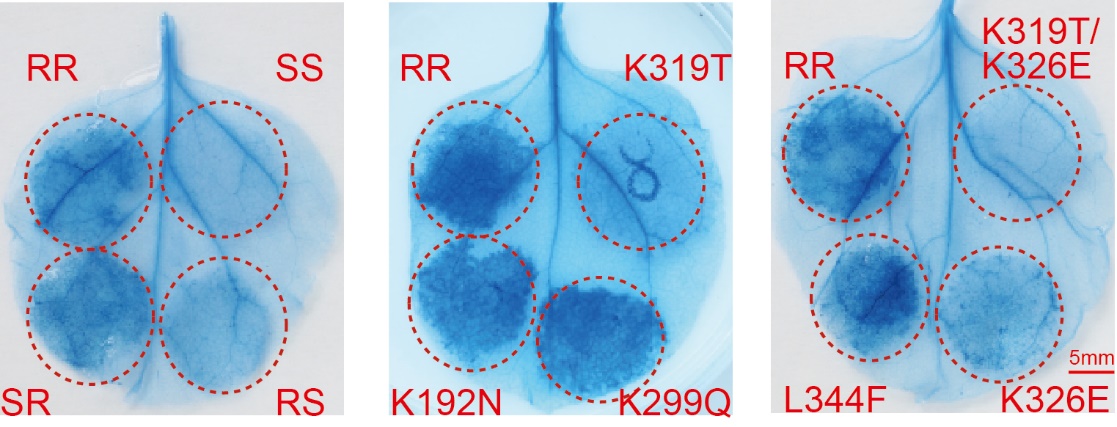


**Fig. S8** **Trypan blue staining of HR necrosis area.** Trypan blue staining to detect dead cells induced by HR response for the infiltration area for Fig. 4b.


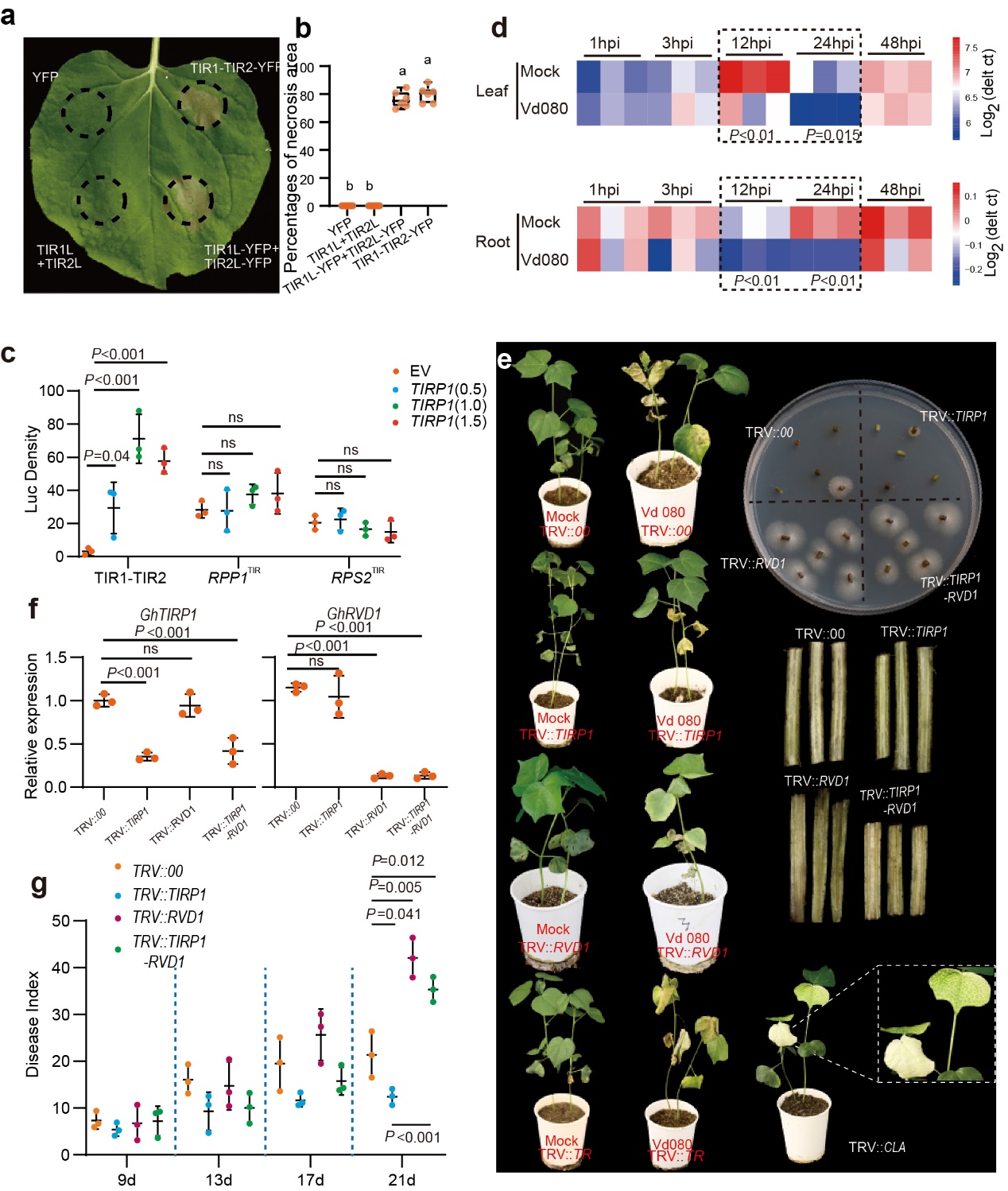


**Fig. S9 Gh*TIRP1* inhibits acquired *V. dahliae* resistance from *GhRVD1* a-b**, TIR1L_R and TIR2L_R with or without YFP-HA. Co-inoculation of TIR1L_R-YFP-HA and TIR2L_R-YFP-HA elicited a HR response. The percentages of necrosis area accounting for the infiltration area for the phenotype. Different letters indicate significant difference at α=0.05 level via One-way ANOVA analysis**. c,** LUC activity within the region co-transformed with LUC plasmid, *TIRP1,* and plant TNL^TIR^ constructs. **d**, Relative expression was measured by qPCR assays, relative expression is indicated on the heat map. Expression patterns of *GhTIRP1* at 1, 3, 12, 24, and 48 hpi with *V. dahliae* strain Vd080 in the roots and leaves of ZZ2. Three independent experiments are represented. **e-g.** Silencing of *TIRP1*, *GhRVD1*, and *TIRP1*-*GhRVD1* (i.e., knockdown the expression of *TIRP1* and *GhRVD1*) in cotton seedlings and inoculated with Vd080. TRV::*00* was used as a negative control, and TRV::*CLA1* was used as a positive control. White leaves were observed in TRV::*CLA1* at approximately 10 dpi. Plant wilt phenotype and stem browning symptoms were photographed at 3 weeks post-inoculation. The expression of target genes was significantly reduced at the transcript level. DI was the mean value of three independent experiments and was measured at 21 dpi. Significant differences were measured between different groups (Student’s t-test).
