# Supplementary material for: Genome-wide association analysis reveals a novel pathway mediated by a dual-TIR domain protein for pathogen resistance in cotton
Source: Genome Biol. 2023 May 10;24:111. doi: 10.1186/s13059-023-02950-9 (PMC10170703; doi:10.1186/s13059-023-02950-9)
Supplement: Supplementary file 2 — Additional file 2: Table S1. Details of lead SNP in each locus. Table S2. Primer sequences used in this study. Table S3. Verticillium wilt phenotyping data of a core collection of upland cotton used in GWAS. Table S4. Gene annotation of candidate genes locating in the introgressed fragment. Table S5. The conserved domain information of TIR-NBS-LRR genes. Table S6. Gene expression profile of an introgressed line under 9- and 72-hour post inoculation of V. dahliae. R1, R2 and R3 represent the three biological replicates. Table S7. Result of systematic Y2H library screening sequencing. Table S8. Exotic introgression analysis of G. hirsutum on chromosome A10. [file 13059_2023_2950_MOESM2_ESM.docx]

| **Table S1 Details of lead SNP in each locus** | | | | | |
| --- | --- | --- | --- | --- | --- |
| Chr. | Lead SNP | *P* value | -log (*P)* | source | ID |
| A10 | A10_113173513 | 2.23E-08 | 7.650 | AYDP | *AYDP1* |
| A13 | A13_87969421 | 2.27E-08 | 7.644 | AYDP | *AYDP2* |
| D03 | D03_3258636 | 4.58E-06 | 5.339 | AYDP | *AYDP3* |
| D07 | D07_10622767 | 9.55E-07 | 6.020 | AYDP | *AYDP4* |
| A05 | A05_14382936 | 8.35E-07 | 6.079 | AYDI | *AYDI1* |
| A09 | A09_78908249 | 1.84E-06 | 5.735 | AYDI | *AYDI2* |
| A13 | A13_87969421 | 8.10E-07 | 6.091 | AYDI | *AYDI3* |
| A08 | A08_115319002 | 2.76E-06 | 5.558 | SHZDP | *SHZDP1* |
| A10 | A10_113173513 | 9.13E-10 | 9.039 | SHZDP | *SHZDP2* |
| A10 | A10_113173420 | 1.51E-06 | 5.820 | SHZDI | *SHZDI1* |
| D05 | D05_1013506 | 4.40E-07 | 6.357 | SHZDI | *SHZDI2* |
| D07 | D07_10578992 | 2.19E-06 | 5.659 | SHZDI | *SHZDI3* |
| D11 | D11_57662952 | 3.42E-06 | 5.466 | SHZDI | *SHZDI4* |
| D12 | D12_56918658 | 4.26E-06 | 5.371 | SHZDI | *SHZDI5* |

**Table S2 Primer sequences used in this study**

| Forward primer name | Forward primer sequence | Reverse primer name | Reverse primer sequence | Experiment |
| --- | --- | --- | --- | --- |
| 156-CG01-F | AAGGTTACCGAATTCTCTAGAAGCAAAGAGAGATATTCATGGATAA | 156-CG01-R | CGTGAGCTCGGTACCGGATCCGAAAAACAAGAGGGTGCCG | VIGS |
| 156-CG02-F | AAGGTTACCGAATTCTCTAGAGTTTTACGATTACCGGAAGGAGG | 156-CG02-R | CGTGAGCTCGGTACCGGATCCAATTCACCGCCATGCGAAA | VIGS |
| 156-CG03-F | AAGGTTACCGAATTCTCTAGAATGCCTCCACGTCCTTTCTTC | 156-CG03-R | CGTGAGCTCGGTACCGGATCCGTCACTTCATGCTCAACCCTTTC | VIGS |
| 156-CG04-F | AAGGTTACCGAATTCTCTAGAATGGCTTCTTCTTCATCTTCTTG | 156-CG04-R | CGTGAGCTCGGTACCGGATCCGTGAAAGTTTCTCCCCTTTCTCTA | VIGS |
| 156-CG05-F | AAGGTTACCGAATTCTCTAGAGCCGATAAGCACGCTACCA | 156-CG05-R | CGTGAGCTCGGTACCGGATCCCTCCCTGCCAAACACCACC | VIGS |
| 156-CG06-F | AAGGTTACCGAATTCTCTAGATTAGAACAGTGGCGGAAAGAAAA | 156-CG06-R | CGTGAGCTCGGTACCGGATCCTTACGCCTCGAACTGCTCAAT | VIGS |
| 156-CG07-F | AAGGTTACCGAATTCTCTAGAGGGGAACCCATAAAACGAAC | 156-CG07-R | CGTGAGCTCGGTACCGGATCCGCACTTACCAAGGGTTTTAGATTC | VIGS |
| 156-CG08-F | AAGGTTACCGAATTCTCTAGAATGCTTCCTCAAAATCATGCCT | 156-CG08-R | CGTGAGCTCGGTACCGGATCCCTTTCCATCGCTTCACTTCATC | VIGS |
| 156-CG09-F | AAGGTTACCGAATTCTCTAGACAATACAATCCACTTGCTCTAAAA | 156-CG09-R | CGTGAGCTCGGTACCGGATCCGCTAGACTTTGCGCCATTATAA | VIGS |
| 156-CG1-F | AAGGTTACCGAATTCTCTAGAAAAGAGTGGGAGAGCGAAGTG | 156-CG1-R | CGTGAGCTCGGTACCGGATCCGTCCTTACCAATCTCCTCAAGC | VIGS |
| 156-CG11-F | AAGGTTACCGAATTCTCTAGAGCTTGGGAGCTATATGTGGGG | 156-CG11-R | CGTGAGCTCGGTACCGGATCCCCTTGTCCTTGTTGAAAATGATG | VIGS |
| 156-CG12-F | AAGGTTACCGAATTCTCTAGACAAGAAAAAAAAGCAGAAACCG | 156-CG12-R | CGTGAGCTCGGTACCGGATCCAGGATATAATACGATGCAACCGAT | VIGS |
| 156-CG13-F | AAGGTTACCGAATTCTCTAGAAGGAGGGTAATAAGGTGTTTTTGG | 156-CG13-R | CGTGAGCTCGGTACCGGATCCCCACGACTCCAAAACTCGC | VIGS |
| 156-CG14-F | AAGGTTACCGAATTCTCTAGATTTCCAGAAATCCCAAGAAGCT | 156-CG14-R | CGTGAGCTCGGTACCGGATCCCTTTTTGGGATTTCTGGGAATT | VIGS |
| Q-CG01-F | TACTTATGCTACAAAGAGCAAAGA | Q-CG01-R | ATCAGTCCCTTGATAAAATGTG | q-PCR |
| Q-CG02-F | AAGGGAAACGGTGTTGAATTC | Q-CG02-R | GACGGCTCGCCAGAGTAGA | q-PCR |
| Q-CG03-F | ATGGCTACTCACTCTCGACCTC | Q-CG03-R | GTCGAGTTGAGCCAGAATCGT | q-PCR |
| Q-CG04-F | AGCTTCAGAGGTGAAGACACG | Q-CG04-R | GAGAGAGTGCATGTGAAAGTTTC | q-PCR |
| Q-CG05-F | TGTCAAAGGAAAGAATGGGAAGA | Q-CG05-R | CACGGCAATATGCAAAGCA | q-PCR |
| Q-CG06-F | GATGAAGGTTGAGGAAGACCG | Q-CG06-R | TAATGTCAAATTTTGCGACCG | q-PCR |
| Q-CG07-F | GCTGCTTCTTCATCATCTTCTAG | Q-CG07-R | ATTGCTCGAGAAAGTGCTTGT | q-PCR |
| Q-CG08-F | GGAGTGAATGTCTTCTTCGATGA | Q-CG08-R | ATGATTTTGAGGAAGCATAGTTTAC | q-PCR |
| Q-CG09-F | ATGGGTGTTAGACATTTTGGTGA | Q-CG09-R | AGACCTTTTATATAGTTGGGAACCC | q-PCR |
| Q-CG1-F | GAAATTACGATATGGCAATATGG | Q-CG1-R | AAGGTCCATATGATTATCTTCGC | q-PCR |
| Q-CG11-F | TGACAAGGGCAATATTCTACATG | Q-CG11-R | AAAGCACCATACCTTGTCTTCC | q-PCR |
| Q-CG12-F | CTCATCGGTTGCATCGTATTATAT | Q-CG12-R | TAACTTTGACGAGACCACCAAAC | q-PCR |
| Q-CG13-F | ATCGAAAGGGTTAGTTGAAATTACC | Q-CG13-R | GGCTTGTACCAAAAACACCTTATTA | q-PCR |
| Q-CG14-F | ACGAAATGAACTCCTTTCTAAACTA | Q-CG14-R | GTTCAACACCCATAAAATCTATTTG | q-PCR |
| 2300-GhRVD1-F | AGAACACGGGGGACTCTAGAATGAAGTATCATGTTTTCTTGAGCT | 2300-GhRVD1-R | TAGTCAGGCGCGCCGGTACCTCATAGCTGAAGCCTCGGTT | pCambia2300-RVD1 |
| QBV3-C-3224-F | GCTCAGGGAAGCTTATCGATGAGATGTCGTCCTCTTCTTCTTCTT | QBV3-C-A3224J1-R | TCTTTGTAGTCCTCGACGATATCTTCTTGCTCAATCAGACCCA | QBV3-C-GA10G322400(PCD) |
| QBV3-C-RVD1-F | GCTCAGGGAAGCTTATCGATATGAAGTATCATGTTTTCTTGAGCT | QBV3-C-RVD1J1-R | TCTTTGTAGTCCTCGACGATGATTCCAACCAATTCATCTGAAG | QBV3-C-RVD1J1 |
| QBV3-C-3208-F | GCTCAGGGAAGCTTATCGATATGGCTTCTTCTTCTTCTTCTTCTTC | QBV3-C-3208J1-R | TCTTTGTAGTCCTCGACGATACCCATTCCGTGAAGTCCTAAT | QBV3-C-3208J1 |
| QBV3-C-3210-F | GCTCAGGGAAGCTTATCGATATGGCTTCTTCTTCTTCTTCTACTC | QBV3-C-3210J1-R | TCTTTGTAGTCCTCGACGATACCACCCATTCCCCAAAA | QBV3-C-3210J1 |
| QBV3-C-3222-F | GCTCAGGGAAGCTTATCGATATGGCTTCTTCTTCTTCTACTTATCATG | QBV3-C-3222J1-R | TCTTTGTAGTCCTCGACGATCATTCCCCAAAGTCCTAATACACG | QBV3-C-3222J1 |
| QBV3-C-3197-F | GCTCAGGGAAGCTTATCGATATGGCGTCTTCTTCATCTTCTCC | QBV3-C-3197J1-R | TCTTTGTAGTCCTCGACGATCATTCTTGGCGAACAATGTCCT | QBV3-C-3197J1 |
| QBV3-C-3201-F | GCTCAGGGAAGCTTATCGATATGGCTTCTTCTTCATCTTCTTGTT | QBV3-C-3201J1-R | TCTTTGTAGTCCTCGACGATGCGAAGGCATCTTTCCATCG | QBV3-C-3201J1 |
| QBV3-C-3204-F | GCTCAGGGAAGCTTATCGATATGATGCTACTAATGATTCTTGTCA | QBV3-C-3204J1-R | TCTTTGTAGTCCTCGACGATTCCCCAGAGTCCTATTACACG | QBV3-C-3204J1 |
| QBV3-C-3220-F | GCTCAGGGAAGCTTATCGATATGTCGTCGTCTTCTTCTTCTTTTC | QBV3-C-3220J1-R | TCTTTGTAGTCCTCGACGATCAAGAAGATTGTCGGGTAATAGGAC | QBV3-C-3220J1 |
| QBV3-C-3224-F | GCTCAGGGAAGCTTATCGATGAGATGTCGTCCTCTTCTTCTTCTT | QBV3-C-3224J1-R | TCTTTGTAGTCCTCGACGATATCTTCTTGCTCAATCAGACCCA | QBV3-C-3224J1 |
| QBV3-C-3233-F | GCTCAGGGAAGCTTATCGATATGGCGTCTTCTTCGTCTTCTC | QBV3-C-3233J1-R | TCTTTGTAGTCCTCGACGATCCCAAAGTCCTATTACACGACTGT | QBV3-C-3233J1 |
| QBV3-C-3237-F | GCTCAGGGAAGCTTATCGATATGGCTGCTTCCTCTTCCTCTT | QBV3-C-3237J1-R | TCTTTGTAGTCCTCGACGATTCCTATAAGACGACTGTCTTCCTGC | QBV3-C-3237J1 |
| QBV3-C-3239-F | GCTCAGGGAAGCTTATCGATATGGCTGCTTCCTCTTCCTCTT | QBV3-C-3239J1-R | TCTTTGTAGTCCTCGACGATACCGACTTCAGCAAAGGCAGT | QBV3-C-3239J1 |
| QBV3-C-3244-F | GCTCAGGGAAGCTTATCGATATGGCTTTTTCCTCTTCTTCTCG | QBV3-C-3244J1-R | TCTTTGTAGTCCTCGACGATTTGATCATCTTTTCCAACAAATTCT | QBV3-C-3244J1 |
| QBV3-C-3246-F | GCTCAGGGAAGCTTATCGAATGATGAATGTCATCTTTCATGATG | QBV3-C-3246J1-R | TCTTTGTAGTCCTCGACGATCCTCAAAATCGTCTTTTTCTGATC | QBV3-C-3246J1 |
| QBV3-C-3193-F | GCTCAGGGAAGCTTATCGATATGTCTTCTTCTTCTTCATCTTCTACT | QBV3-C-3193J1-R | TCTTTGTAGTCCTCGACGATCAATCAGACCCAGAATTATCTTTT | QBV3-C-3193J1 |
| QBV3-C-3194-F | GCTCAGGGAAGCTTATCGATATGGATATTTTACGCTTTTCTTCTT | QBV3-C-3194J1-R | TCTTTGTAGTCCTCGACGATTGTAATAGGGCTTTGGGGAA | QBV3-C-3194J1 |
| QBV3-C-3196-F | GCTCAGGGAAGCTTATCGATATGGCGTCTTCTTCAACTTCTTCT | QBV3-C-3196J1-R | TCTTTGTAGTCCTCGACGATTCCTAATACACGACAGTCTTTTTGC | QBV3-C-3196J1 |
| QBV3-C-3199-F | GCTCAGGGAAGCTTATCGATATGGCGTCTTCTTCATCTTCTTCT | QBV3-C-3199J1-R | TCTTTGTAGTCCTCGACGATGATGCCCGCTTGCTCAATT | QBV3-C-3199J1 |
| QBV3-C-3206-F | GCTCAGGGAAGCTTATCGATATGCTCTTCGGCAATTTAGC | QBV3-C-3206J1-R | TCTTTGTAGTCCTCGACGATTACACGACTGCCTTTTTTCTCA | QBV3-C-3206J1 |
| QBV3-C-3216-F | GCTCAGGGAAGCTTATCGATATGGCCTCGCCTTCTTCTT | QBV3-C-3216J1-R | TCTTTGTAGTCCTCGACGATTTTCTCTTTCTGGTCATCTATTCC | QBV3-C-3216J1 |
| QBV3-C-3217-F | GCTCAGGGAAGCTTATCGATATGGCTTCTTCTTCTTCTTGTTCA | QBV3-C-3217J1-R | TCTTTGTAGTCCTCGACGATTCCTATTACACGGATGTCCGC | QBV3-C-3217J1 |
| QBV3-C-3218-F | GCTCAGGGAAGCTTATCGATATGCCTTCTTCTTCATCTTCTTGTT | QBV3-C-3218J1-R | TCTTTGTAGTCCTCGACGATAAATCCTATTACACGGATGTCCG | QBV3-C-3218J1 |
| QBV3-C-3219-F | GCTCAGGGAAGCTTATCGATATGGCTTCTTCTTCATCTTCATCTT | QBV3-C-3219J1-R | TCTTTGTAGTCCTCGACGATACGACTGTCTTCTTGATCAATCAAT | QBV3-C-3219J1 |
| QBV3-C-3230-F | GCTCAGGGAAGCTTATCGATATGGAGTCTTCTTCATCTTCTTCTC | QBV3-C-3230J1-R | TCTTTGTAGTCCTCGACGATAAGCCCTATTACACGACAATCTT | QBV3-C-3230J1 |
| QBV3-C-3231-F | GCTCAGGGAAGCTTATCGATATGGCGTCTTCGTCTGCTC | QBV3-C-3231J1-R | TCTTTGTAGTCCTCGACGATTATTACACGACTGTCCGCTTGC | QBV3-C-3231J1 |
| QBV3-C-3232-F | GCTCAGGGAAGCTTATCGATATGGCTGCTTCTTCATCATCTTCT | QBV3-C-3232J1-R | TCTTTGTAGTCCTCGACGATACAGTCATTTTGCTTAATCAGCTTCA | QBV3-C-3232J1 |
| QBV3-C-3234-F | GCTCAGGGAAGCTTATCGATATGGCTGCTTCTTCTTCTTCTTC | QBV3-C-3234J1-R | TCTTTGTAGTCCTCGACGATAAGTCCTATTAGACGACTGTCTTCCT | QBV3-C-3234J1 |
| QBV3-C-3243-F | GCTCAGGGAAGCTTATCGATATGGCTGCTTCTTCTTCTTCTTC | QBV3-C-3243J1-R | TCTTTGTAGTCCTCGACGATATGGCTGCTTCTTCTTCTTCTTC | QBV3-C-3243J1 |
| RS-F(SR-F) | AGCATCTCGGATAGACTTGAAACC | RS-R(SR-R) | GGTTTCAAGTCTATCCGAGATGCT | Mutant |
| TIR1-TIR2-K192N –F | CACCTAGTCAATGCTTTGGAAAAAG | TIR1-TIR2-K192N –R | TTCCAAAGCATTGACTAGGTGATTG | Mutant |
| TIR1-TIR2-K299Q-F | ATAGTACAACAATGGAAATCTGCTTTTGC | TIR1-TIR2- K299Q-R | ATTTCCATTGTTGTACTATATCAGCTGG | Mutant |
| TIR1-TIR2- K319T-F | AAGGAGGGACATTTGACAGACCTG | TIR1-TIR2- K319T-R | TGTCAAATGTCCCTCCTTCTATATGC | Mutant |
| TIR1-TIR2- K326E-F | CCTGAAACCGAGTACGTTGAAAATG | TIR1-TIR2- K326E-R | CAACGTACTCGGTTTCAGGTCTGTC | Mutant |
| QBV3-C-TIR1-TIR2-F | GCTCAGGGAAGCTTATCGATATGAAGTATCATGTTTTCTTGAGCTTCAGAGGTGAAG | QBV3-C-TIR1-TIR2- L326F-R | TCTTTGTAGTCCTCGACGATGATTCCAACCAATTCATCTGAAGCACTTCTAAACT | Mutant |
| TIR1-TIR2-NH188189AA-F | AACTTCTCCGCTGCCCTAGTCAAAG | TIR1-TIR2-NH188189AA-R | CTTTGACTAGGGCAGCGGAGAAGTT | Mutant |
| TIR1-TIR2-TY2021AA-F | AATTTCACCGCTGCCTTACTTCAAGCTTTGG | TIR1-TIR2-TY2021AA-F | TTGAAGTAAGGCAGCGGTGAAATTAAGGCG | Mutant |
| TIR1-TIR2-G142R-F | GTAAATTAAAACGGTGGCCTATAGA | TIR1-TIR2-G142R-R | TCTATAGGCCACCGTTTTAATTTAC | Mutant |
| TIR1-TIR2-G312R-F | CGGTAAATTAAAACGGTGGCATA | TIR1-TIR2-G312R-R | TATGCCACCGTTTTAATTTACCG | Mutant |
| TIR1-TIR2-E77A-F | CATGCTTGGCTGCACTCTCTGACAT | TIR1-TIR2-E77A-R: | ATGTCAGAGAGTGCAGCCAAGCATG | Mutant |
| TIR1-TIR2-E245A-F | GATATCGAGCTTGGTTGCACTTTCT | TIR1-TIR2-E245A-R: | AGAAAGTGCAACCAAGCTCGATATC | Mutant |
| QBV3-C-RVD1-F | GCTCAGGGAAGCTTATCGATATGAAGTATCATGTTTTCTTGAGCT | QBV3-C-TIR1-R | TCTTTGTAGTCCTCGACGATCAACTTTTGCATAACATGCGC | 100-TIR1 |
| QBV3-C-TIR2-F | GCTCAGGGAAGCTTATCGATATGAAGCATCAAGTTTTCGTAAGC | QBV3-C-TIR1-TIR2-R | TCTTTGTAGTCCTCGACGATGATTCCAACCAATTCATCTGAAG | 100-TIR2 |
| QBV3-C-RVD1-F | GCTCAGGGAAGCTTATCGATATGAAGTATCATGTTTTCTTGAGCT | QBV3-C-TIR1H-R | TCTTTGTAGTCCTCGACGATTAGGTGATTGGAGAAGTTGAGGC | 100-TIR1L |
| QBV3-C-TIR2L-F | GCTCAGGGAAGCTTATCGATATGTCGGATAGACTTGAAACCACG | QBV3-C-TIR2L-R | TCTTTGTAGTCCTCGACGATACAGTCTTTTTGCTTAATCCGC | 100-TIR2L |
| QBV3-C-RVD1-F | GCTCAGGGAAGCTTATCGATATGAAGTATCATGTTTTCTTGAGCT | QBV3-C-AJ2-R | TCTTTGTAGTCCTCGACGATACAGTCTTTTTGCTTAATCCGC | 100-AJ2 |
| QBV3-C-RVD1-F | GCTCAGGGAAGCTTATCGATATGAAGTATCATGTTTTCTTGAGCT | QBV3-C-AJ3-R | TCTTTGTAGTCCTCGACGATTCCAAGAAACCGATGACTTTCA | 100-AJ3 |
| QBV3-C-RVD1-F | GCTCAGGGAAGCTTATCGATATGAAGTATCATGTTTTCTTGAGCT | QBV3-C-RVD1J4-R | TCTTTGTAGTCCTCGACGATTTCTGGTTCTAGCCCATGATAAC | 100-AJ4 |
| qbv3C- RVD1-347-F | GCTCAGGGAAGCTTATCGATATGGCTTCAGATGAATTGGTTGGAATC | qbv3C- RVD1-653-R | TCTTTGTAGTCCTCGACGATCTCTTTGCTGTTTGTAGCATAAGTAG | 100-NBS |
| qbv3C- RVD1LR645-F | GCTCAGGGAAGCTTATCGATATGACTTATGCTACAAACAGCAAAGAGA | QBV3-C-RVD1-R | TCTTTGTAGTCCTCGACGATTAGCTGAAGCCTCGGTTGTG | 100-LRR |
| QBV3-C-RVD1-F | GCTCAGGGAAGCTTATCGATATGAAGTATCATGTTTTCTTGAGCT | QBV3-C-RVD1-R | TCTTTGTAGTCCTCGACGATTAGCTGAAGCCTCGGTTGTG | 100-GhRVD1 |
| 4myc-TIR1-TIR2-F | ATTTGAATGGCTCCGGATCCATGAAGTATCATGTTTTCTTGAGCTTCAGA | 4myc-TIR1-TIR2-R | GAAGACAGAGCTAGTTACATGGATCCTTAGATTCCAACCAATTCATCTGA | 4myc-TIR1-TIR2 |
| TIR1-TIR2-3HA-F | AACACGGGGGACTTTGCAACGGATCCATGAAGTATCATGTTTTCTT | TIR1-TIR2-3HA-R | AGGAACATCGTATGGGTACATGGATCCGATTCCAACCAATTCATCTGAA | TIR1-TIR2-3HA |
| T1L-3HA-F | CACGGGGGACTTTGCAACGGATCCATGAAGTATCATGTTTTCTTG | T1L-3HA-R | GAACATCGTATGGGTACATGGATCCTAGGTGATTGGAGAAGTTGAGGCGT | TIR1-TIR2-3HA |
| T2L-4Myc-F | AGAACACGGGGGACTTTGCAACATGTCGGATAGACTTGAAACCACG | T2L-4Myc-R | TCACCGTTAATCAAACCCATACAGTCTTTTTGCTTAATCCGC | TIR1-TIR2-4Myc |
| TIRP1-GFP-F | TGGAGAGAACACGGGGGACTTTGCAACATGGCAACTTCGTTTAGCTCTCC | TIRP1-GFP-R | CACTCCCTGAAGCGGCCGCTGTACATGCAGGTGCAATGGAGACTTTATCA | TIRP1-GFP |
| T1L-3HA-F | AACACGGGGGACTTTGCAACGGATCCATGAAGTATCATGTTTTCTT | T1L-AEM-3HA-R | GAACATCGTATGGGTACATGGATCCTAGGGCAGCGGAGAAGTTGAG | T1H-3HA(AE-M) |
| QBV3-RVD1-F | CAAAAAAGCAGGCTCAGGGGATATCATGAAGTATCATGTTTTCTTGAGCT | QBV3-TIR1-TIR2-R | AAAGCTGGGTGCAGGGCGATATCGATTCCAACCAATTCATCTGAAG | 101-TIR1-TIR2 |
| QBV3-RVD1-F | CAAAAAAGCAGGCTCAGGGGATATCATGAAGTATCATGTTTTCTTGAGCT | QBV3-T1L-R | AAAGCTGGGTGCAGGGCGATATCTAGGTGATTGGAGAAGTTGAGGC | 101-T1L |
| QBV3-T2L-F | CAAAAAAGCAGGCTCAGGGGATATCATGTCGGATAGACTTGAAACCACG | QBV3-T2L-R | AAAGCTGGGTGCAGGGCGATATCACAGTCTTTTTGCTTAATCCGC | 101-T2L |
| QBV3-RVD1-F | CAAAAAAGCAGGCTCAGGGGATATCATGAAGTATCATGTTTTCTTGAGCT | QBV3-T1L-AEM-R | AAAGCTGGGTGCAGGGCGATATCTAGGGCAGCGGAGAAGTTGAG | T1L-YFP-HA(AE-M) |
| AD-YFP-F | GCCATGGAGGCCAGTGAATTCATGGTGAGCAAGGGCGAGGAG | AD-YFP-R | TGCAGCTCGAGCTCGATGGATCCCCTTGTACAGCTCGTCCATGCC | AD-YFP |
| BD-YFP-F | CATATGGCCATGGAGGCCGAATTCATGGTGAGCAAGGGCGAGGAG | BD-YFP-R | TGCGGCCGCTGCAGGTCGACGCTTGTACAGCTCGTCCATGCC | BD-YFP |
| AD-RVD1-F | GCCATGGAGGCCAGTGAATTCATGAAGTATCATGTTTTCTTGAGCT | AD-T1L-R | TGCAGCTCGAGCTCGATGGATCCCTAGGTGATTGGAGAAGTTGAGGC | AD-T1L |
| BD-T2L-F | CATATGGCCATGGAGGCCGAATTCATGTCGGATAGACTTGAAACCACG | BD-T2L-R | TGCGGCCGCTGCAGGTCGACGACAGTCTTTTTGCTTAATCCGC | BD-T2L |
| AD-RVD1-F | GCCATGGAGGCCAGTGAATTCATGAAGTATCATGTTTTCTTGAGCT | AD-T1L-YFP-R | TGCAGCTCGAGCTCGATGGATCCCCTTGTACAGCTCGTCCATGCC | AD-T1L-YFP |
| BD-T2L-F | CATATGGCCATGGAGGCCGAATTCATGTCGGATAGACTTGAAACCACG | BD-T2L-YFP-R | TGCGGCCGCTGCAGGTCGACGCTTGTACAGCTCGTCCATGCC | BD-T2L-YFP |
| AD-RVD1-F | GCCATGGAGGCCAGTGAATTCATGAAGTATCATGTTTTCTTGAGCT | AD-T1L-R | TGCAGCTCGAGCTCGATGGATCCCTAGGTGATTGGAGAAGTTGAGGC | AD-T1L-AEM-YFP |
| NLUC-RVD1-F | GAGAACACGGGGGACGAGCTCATGAAGTATCATGTTTTCTTGAGCT | NLUC-TIR1-TIR2-R | CGCGTACGAGATCTGGTCGACGATTCCAACCAATTCATCTGAAG | NLUC-TIR1-TIR2 |
| CLUC-RVD1-F | TACGCGTCCCGGGGCGGTACCATGAAGTATCATGTTTTCTTGAGCT | CLUC-TIR1-TIR2-R | ACGAAAGCTCTGCAGGTCGACGATTCCAACCAATTCATCTGAAG | CLUC-TIR1-TIR2 |
| NLUC-T2L-F | GAGAACACGGGGGACGAGCTCATGTCGGATAGACTTGAAACCACG | NLUC-T2L-R | CGCGTACGAGATCTGGTCGACACAGTCTTTTTGCTTAATCCGC | NLUC-T2L |
| CLUC-RVD1-F | TACGCGTCCCGGGGCGGTACCATGAAGTATCATGTTTTCTTGAGCT | CLUC-T1L-R | ACGAAAGCTCTGCAGGTCGACTCATAGGTGATTGGAGAAGTTGAGGC | CLUC-T1L |
| CLUC-RVD1-F | TACGCGTCCCGGGGCGGTACCATGAAGTATCATGTTTTCTTGAGCT | CLUC-T1L(AE-M)-R | ACGAAAGCTCTGCAGGTCGACTCATAGGGCAGCGGAGAAGTTGAG | CLUC-T1L(AE-M) |
| T7 | TAATACGACTCACTATAGGGC | 3'AD | AGATGGTGCACGATGCACAG | Y2H MATING |
| AD-TIRP1-F | GCCATGGAGGCCAGTGAATTCATGGCAACTTCGTTTAGCTCTC | AD-TIRP1-R | TGCAGCTCGAGCTCGATGGATCCCTGCAGGTGCAATGGAGACTT | AD-TIRP1 |
| BD-RVD1-F | CATATGGCCATGGAGGCCGAATTCATGAAGTATCATGTTTTCTTGAGCT | BD-TIR1-TIR2-R | TGCGGCCGCTGCAGGTCGACGGATTCCAACCAATTCATCTGAAG | BD-TIR1-TIR2 |
| BD- L629-F | CATATGGCCATGGAGGCCGAATTCAAAGACTCAATCGTCAACGATGA | BD- L6248-R | TGCGGCCGCTGCAGGTCGACGTAATACGGCTGTTATGTGATCATCA | BD-L6(29-248) |
| BD-RPP1-F | CATATGGCCATGGAGGCCGAATTCATGGGTTCTGCAATGAGCTTG | BD-RPP1J1-R | TGCGGCCGCTGCAGGTCGACGATCCAACATGTTTGAAACATCAGTG | BD-RPP1_Nad(1-254) |
| BD- RPS4-F | CATATGGCCATGGAGGCCGAATTCATGGAGACATCATCTATTTCCACTG | BD- RPS4183-R | TGCGGCCGCTGCAGGTCGACGACTTCCCTCCGGTGGTATTCC | BD-RPS4(1-183) |
| qbv3c- L629-F | GCTCAGGGAAGCTTATCGATAAAGACTCAATCGTCAACGATGA | qbv3c- L6248-R | TCTTTGTAGTCCTCGACGATGTAATACGGCTGTTATGTGATCATCA | 100-L6(29-248) |
| qbv3c-RPP1-F | GCTCAGGGAAGCTTATCGATATGGGTTCTGCAATGAGCTTG | qbv3c-RPP1J1-R | TCTTTGTAGTCCTCGACGATGATCCAACATGTTTGAAACATCAGTG | 100-RPP1_Nad(1-254) |
| qbv3c- RPS4-F | GCTCAGGGAAGCTTATCGATATGGAGACATCATCTATTTCCACTG | qbv3c- RPS4183-R | TCTTTGTAGTCCTCGACGATGACTTCCCTCCGGTGGTATTCC | 100-RPS4(1-183) |
| 2300-TIR1-TIR2-GFP-F | CTGTACAAGGGTACCATGAAGTATCATGTTTTCTTGAGCT | 2300-TIR1-TIR2-GFP-R | CTGCTCAGGCGCGCCGATTCCAACCAATTCATCTGAAG | 2300-TIR1-TIR2-YFP |
| 2300-TIRP1-GFP-F | CTGTACAAGGGTACCATGGCAACTTCGTTTAGCTCTC | 2300-TIRP1-GFP-R | CTGCTCAGGCGCGCCTGCAGGTGCAATGGAGACTT | 2300-TIRP1-YFP |
| 2300-RVD1-GFP-F | CTGTACAAGGGTACCATGAAGTATCATGTTTTCTTGAGCT | 2300-RVD1-GFP-R | CTGCTCAGGCGCGCCTAGCTGAAGCCTCGGTTGTG | 2300-RVD1-YFP |
| Q-TIRP1-F | GCCTAATGGCATAAGGTACTATGAG | Q-TIRP1-R | TATCAACAAATACTTCACCTCTGCT | qrt-pcr |
| 156-TIRP1-F | AAGGTTACCGAATTCTCTAGAATCCACCACCATCTCCTAAAT | 156-TIRP1-R | CGTGAGCTCGGTACCGGATCCAGAAACTTCAATGCGAGTCTTAA | 156-TIRP1 |
| RVD1-F | CAAGACAGGCTCAACAAGACC | RVD1-TIRP1-R | GGTCTTGTTGAGCCTGTCTTGAGAAACTTCAATGCGAGTCTTAA | 156-TIRP1-GhRVD1 |
| 104-TIRP1-F | CAATTACAGGTACCCGGGGATCCATGGCAACTTCGTTTAGCTCTC | 104-TIRP1-R | CTGCCACCGCCGTCGACTCTAGATGCAGGTGCAATGGAGACTT | TIRP1-Cyfp |
| 106-RVD1-F | CATCGAGGACGCCGGCGGATCCATGAAGTATCATGTTTTCTTGAGCT | 106-RVD1-R | GAAAGCTCTGCAGGTCGACTCTAGATAGCTGAAGCCTCGGTTGTG | Nyfp-RVD1 |
| 106-RVD1-F | CATCGAGGACGCCGGCGGATCCATGAAGTATCATGTTTTCTTGAGCT | 106-TIR1-TIR2-R | GAAAGCTCTGCAGGTCGACTCTAGAGATTCCAACCAATTCATCTGAAG | Nyfp-TIR1-TIR2 |
| CG1-RS-F | GAAGCGGATTAAGCAAAAAGACTGT | CG1-RS-R | ACAGTCTTTTTGCTTAATCCGCTTC | 2300-GhRVD1_R/S |
| 067-10proH3-F | GCCAGTGCCAAGCTAAGCTTGCAGTTAGAAGACTTCAGTATATGCTG | 067-10proH3-R | ATGGATCCTCTAGAGTCGACCTGCAGTAGAGTAGAAGATGAAGAAGAAGCCAC | Native pro- GhRVD1 |
| 067P10-CG1-F | CATCTTCTACTCTACTGCAGATGAAGTATCATGTTTTCTTGAGCT | 067P10-CG1J5-R | ATGGATCCTCTAGAgtcgacTAGCTGAAGCCTCGGTTGTG | Native pro- GhRVD1 |

**Table S3 *Verticillium* wilt phenotyping data of a core collection of upland cotton used in GWAS**

| Taxa ID | Source | AYDP(%) | AYDI | SHZDP(%) | SHZDI |
| --- | --- | --- | --- | --- | --- |
| L044 | Henan, CHN | 70.59 | 39.71 | 100.00 | 38.51 |
| F016 | Missouri, USA | 100.00 | 48.75 | 94.74 | 67.11 |
| L017 | Henan, CHN | 100.00 | 54.76 | 95.24 | 79.17 |
| D077 | Henan, CHN | 94.44 | 37.50 | 97.44 | 71.15 |
| L087 | Henan, CHN | 94.44 | 50.00 | 100.00 | 85.71 |
| L099 | Henan, CHN | 100.00 | 51.25 | 70.73 | 46.34 |
| D019 | Hebei, CHN | 95.00 | 47.50 | 85.71 | 68.57 |
| L014 | Shandong, CHN | 95.00 | 51.25 | 92.86 | 70.83 |
| D038 | Shandong, CHN | 100.00 | 47.22 | 81.82 | 51.52 |
| D081 | Shanghai, CHN | 92.86 | 37.50 | 69.44 | 40.28 |
| L057 | Shanxi, CHN | 94.74 | 61.84 | 78.95 | 48.68 |
| F079 | Jiangsu, CHN | 100.00 | 51.39 | 97.73 | 80.11 |
| L123 | Hunan, CHN | 100.00 | 63.16 | 95.12 | 62.80 |
| D005 | Hunan, CHN | 93.33 | 33.33 | 87.50 | 50.00 |
| L047 | Hubei, CHN | 88.24 | 54.41 | 100.00 | 46.32 |
| L073 | Hubei, CHN | 100.00 | 47.83 | 72.73 | 36.36 |
| L012 | Sichuan, CHN | 83.33 | 29.17 | 82.35 | 41.91 |
| D072 | Jiangsu, CHN | 100.00 | 44.74 | 97.44 | 71.15 |
| D014 | Shanghai, CHN | 95.24 | 42.86 | 76.32 | 43.42 |
| L001 | Zhejiang, CHN | 95.24 | 60.71 | 91.67 | 63.19 |
| D002 | Zhejiang, CHN | 95.24 | 41.67 | 75.86 | 25.86 |
| D041 | Jiangxi, CHN | 94.44 | 58.33 | 78.57 | 44.05 |
| L002 | Yunnan, CHN | 82.35 | 35.29 | 77.78 | 29.63 |
| L056 | Hunan, CHN | 73.68 | 36.84 | 86.84 | 42.11 |
| L069 | Shaanxi, CHN | 70.83 | 26.04 | 96.77 | 41.94 |
| D090 | Henan, CHN | 85.00 | 35.00 | 70.37 | 23.15 |
| L075 | Hubei, CHN | 95.00 | 41.25 | 66.67 | 26.67 |
| L100 | Shaanxi, CHN | 95.00 | 66.25 | 100.00 | 68.06 |
| D008 | Hubei, CHN | 95.24 | 38.10 | 85.29 | 50.00 |
| D073 | Jiangsu, CHN | 100.00 | 55.88 | 72.73 | 40.91 |
| D071 | Jiangsu, CHN | 88.89 | 40.28 | 78.79 | 36.36 |
| D050 | Jiangsu, CHN | 80.00 | 45.00 | 94.12 | 30.15 |
| L065 | Anhui, CHN | 94.44 | 44.44 | 91.89 | 57.43 |
| L109 | Hebei, CHN | 95.00 | 56.25 | 97.56 | 72.56 |
| L007 | Shanghai, CHN | 90.00 | 41.25 | 59.46 | 37.84 |
| B065 | Hebei, CHN | 85.71 | 39.29 | 86.67 | 57.50 |
| D097 | Henan, CHN | 92.00 | 37.00 | 68.57 | 42.14 |
| L062 | Hebei, CHN | 73.33 | 35.00 | 100.00 | 30.47 |
| D016 | Hebei, CHN | 100.00 | 43.06 | 81.82 | 48.48 |
| D007 | Hubei, CHN | 94.44 | 62.50 | 70.73 | 57.32 |
| L015 | Hubei, CHN | 94.12 | 38.24 | 62.16 | 45.27 |
| D100 | Henan, CHN | 93.75 | 42.19 | 100.00 | 43.29 |
| D093 | Henan, CHN | 88.24 | 45.59 | 100.00 | 46.70 |
| D031 | Beijing, CHN | 93.33 | 36.67 | 100.00 | 37.90 |
| D032 | Beijing, CHN | 93.75 | 48.44 | 100.00 | 49.50 |
| L103 | Shandong, CHN | 100.00 | 68.42 | 75.61 | 54.27 |
| L088 | Henan, CHN | 100.00 | 50.00 | 100.00 | 74.29 |
| L086 | Henan, CHN | 95.00 | 52.50 | 97.44 | 58.97 |
| D101 | Henan, CHN | 95.00 | 48.75 | 80.56 | 59.03 |
| L045 | Guangxi, CHN | 38.89 | 19.44 | 50.00 | 19.38 |
| L079 | Pakistan | 94.74 | 51.32 | 92.31 | 80.77 |
| D052 | Jiangsu, CHN | 27.27 | 7.96 | 40.63 | 13.28 |
| D026 | Shanxi, CHN | 83.33 | 51.39 | 62.79 | 42.44 |
| L005 | Xinjiang, CHN | 100.00 | 51.32 | 100.00 | 52.40 |
| L059 | Xinjiang, CHN | 47.37 | 15.79 | 94.74 | 32.89 |
| L010 | USA | 89.47 | 38.16 | 100.00 | 40.00 |
| B070 | Beijing, CHN | 82.35 | 39.71 | 90.00 | 70.00 |
| L040 | Shandong, CHN | 72.22 | 26.39 | 62.16 | 44.59 |
| L126 | Hubei, CHN | 94.12 | 38.24 | 95.00 | 39.30 |
| D096 | Henan, CHN | 100.00 | 50.00 | 79.41 | 60.29 |
| L021 | Liaoning, CHN | 100.00 | 43.42 | 100.00 | 44.50 |
| D022 | Liaoning, CHN | 100.00 | 56.25 | 74.36 | 48.08 |
| L094 | Shandong, CHN | 94.12 | 66.18 | 100.00 | 41.67 |
| F019 | Bulgaria | 93.75 | 50.00 | 89.19 | 64.86 |
| D036 | Liaoning, CHN | 100.00 | 44.12 | 80.00 | 44.29 |
| D098 | Henan, CHN | 100.00 | 61.11 | 86.84 | 52.63 |
| L072 | Henan, CHN | 89.47 | 36.84 | 91.67 | 72.22 |
| L093 | Liaoning, CHN | 85.71 | 29.76 | 88.10 | 25.00 |
| B015 | Hebei, CHN | 100.00 | 57.50 | 88.89 | 71.67 |
| D044 | Shaanxi, CHN | 100.00 | 56.94 | 100.00 | 61.31 |
| D045 | Shaanxi，CHN | 100.00 | 48.33 | 90.20 | 65.69 |
| D003 | Sichuan, CHN | 100.00 | 60.94 | 90.91 | 81.25 |
| B075 | Hebei, CHN | 100.00 | 51.79 | 100.00 | 55.00 |
| L090 | Hubei, CHN | 86.36 | 34.09 | 71.05 | 35.53 |
| B081 | Henan, CHN | 100.00 | 50.00 | 93.02 | 61.63 |
| D082 | Shanxi, CHN | 73.68 | 26.32 | 70.59 | 36.03 |
| D079 | Shanxi, CHN | 93.75 | 42.19 | 75.00 | 33.75 |
| D080 | Shanxi, CHN | 77.27 | 36.36 | 74.29 | 36.43 |
| D035 | Liaoning, CHN | 88.89 | 45.83 | 54.05 | 32.43 |
| L032 | Xinjiang, CHN | 95.24 | 42.86 | 63.16 | 26.97 |
| L031 | Xinjiang, CHN | 95.24 | 36.90 | 91.43 | 47.86 |
| L033 | Xinjiang, CHN | 100.00 | 42.19 | 100.00 | 48.21 |
| D012 | Hebei, CHN | 94.74 | 50.00 | 86.84 | 39.47 |
| D076 | Henan, CHN | 100.00 | 42.19 | 63.64 | 31.06 |
| L034 | Xinjiang, CHN | 76.19 | 35.71 | 78.13 | 35.16 |
| B073 | Hebei, CHN | 95.24 | 41.67 | 100.00 | 42.70 |
| B034 | Hebei, CHN | 78.95 | 50.00 | 71.05 | 36.18 |
| D063 | Xinjiang, CHN | 87.50 | 56.25 | 100.00 | 78.95 |
| L036 | Shaanxi, CHN | 31.82 | 10.23 | 57.89 | 32.24 |
| L042 | Henan, CHN | 83.33 | 52.78 | 90.24 | 35.37 |
| L105 | Henan, CHN | 94.12 | 52.94 | 97.14 | 60.00 |
| L043 | Guizhou, CHN | 100.00 | 63.33 | 87.80 | 40.24 |
| L053 | Xinjiang, CHN | 88.24 | 29.41 | 88.57 | 29.29 |
| D042 | Shaanxi，CHN | 60.00 | 23.75 | 94.44 | 44.44 |
| L125 | Shanxi, CHN | 100.00 | 47.62 | 85.29 | 51.47 |
| L121 | Jiangsu, CHN | 83.33 | 40.28 | 100.00 | 45.20 |
| L018 | Guangxi, CHN | 100.00 | 38.16 | 91.67 | 67.36 |
| L019 | Guangxi, CHN | 100.00 | 46.05 | 91.67 | 50.00 |
| L016 | Yunnan, CHN | 100.00 | 41.25 | 83.33 | 36.90 |
| D069 | Xinjiang, CHN | 94.44 | 50.00 | 69.44 | 37.50 |
| B079 | Jiangsu, CHN | 71.43 | 35.71 | 51.52 | 16.67 |
| D075 | Henan, CHN | 100.00 | 37.50 | 78.05 | 37.80 |
| B013 | Hebei, CHN | 94.12 | 27.94 | 54.35 | 24.46 |
| B021 | Shanxi, CHN | 94.12 | 32.35 | 92.30 | 33.40 |
| B060 | Beijing, CHN | 100.00 | 37.50 | 74.36 | 34.62 |
| L023 | Jiangsu, CHN | 95.45 | 35.23 | 71.05 | 51.32 |
| L028 | Sichuan, CHN | 90.91 | 34.09 | 86.67 | 63.33 |
| L122 | Jiangsu, CHN | 95.00 | 33.75 | 93.94 | 55.30 |
| D067 | Xinjiang, CHN | 94.44 | 68.06 | 94.44 | 47.22 |
| L111 | Xinjiang, CHN | 100.00 | 45.24 | 80.56 | 68.75 |
| D046 | Shaanxi，CHN | 81.25 | 34.38 | 97.44 | 66.67 |
| D010 | Jiangxi, CHN | 94.12 | 39.71 | 72.22 | 45.14 |
| L089 | Beijing, CHN | 94.12 | 41.18 | 100.00 | 42.40 |
| D059 | Jiangsu, CHN | 62.50 | 28.13 | 94.74 | 38.16 |
| D057 | Jiangsu, CHN | 56.25 | 25.00 | 53.85 | 25.00 |
| L082 | Jiangsu, CHN | 93.75 | 39.06 | 86.67 | 57.50 |
| B049 | Henan, CHN | 88.89 | 30.56 | 64.00 | 33.00 |
| B050 | Henan, CHN | 100.00 | 40.48 | 87.50 | 57.50 |
| B025 | Liaoning, CHN | 100.00 | 38.16 | 70.73 | 49.39 |
| L095 | Shandong, CHN | 95.00 | 58.75 | 94.74 | 38.16 |
| L102 | Liaoning, CHN | 78.95 | 48.68 | 94.44 | 81.25 |
| L115 | Shanxi, CHN | 85.00 | 46.25 | 84.62 | 41.67 |
| B055 | Henan, CHN | 52.63 | 13.16 | 51.61 | 19.35 |
| D013 | Henan, CHN | 90.00 | 37.50 | 92.50 | 69.38 |
| D027 | Gansu, CHN | 94.74 | 43.42 | 81.08 | 43.24 |
| B020 | Shanxi, CHN | 100.00 | 34.09 | 80.56 | 31.25 |
| D089 | Henan, CHN | 90.48 | 32.14 | 67.74 | 35.48 |
| D064 | Xinjiang, CHN | 94.44 | 66.67 | 66.67 | 36.67 |
| D065 | Xinjiang, CHN | 100.00 | 55.88 | 90.32 | 70.97 |
| D023 | Shanxi, CHN | 94.74 | 30.26 | 100.00 | 59.72 |
| D068 | Xinjiang, CHN | 85.00 | 46.25 | 85.00 | 49.38 |
| L071 | Hebei, CHN | 100.00 | 46.05 | 100.00 | 66.22 |
| L081 | Hubei, CHN | 100.00 | 36.90 | 94.44 | 63.19 |
| L030 | Hubei, CHN | 95.00 | 61.25 | 100.00 | 84.48 |
| D048 | Shaanxi, CHN | 83.33 | 50.00 | 100.00 | 70.63 |
| L112 | Hebei, CHN | 100.00 | 60.53 | 96.43 | 79.46 |
| D049 | Shaanxi，CHN | 100.00 | 38.10 | 97.14 | 73.57 |
| L052 | Sichuan, CHN | 66.67 | 33.33 | 97.50 | 66.88 |
| D021 | Jiangsu, CHN | 61.90 | 32.14 | 79.31 | 34.48 |
| L054 | Shaanxi，CHN | 90.91 | 42.05 | 56.25 | 32.03 |
| L050 | Guizhou, CHN | 78.95 | 39.47 | 77.50 | 46.88 |
| L003 | Shandong, CHN | 94.74 | 51.32 | 88.57 | 41.43 |
| L009 | Beijing, CHN | 87.50 | 39.58 | 97.22 | 38.89 |
| L008 | Hebei, CHN | 70.00 | 33.75 | 82.35 | 26.47 |
| B076 | Henan, CHN | 100.00 | 65.28 | 82.05 | 34.62 |
| L067 | Hebei, CHN | 91.30 | 53.26 | 83.33 | 36.81 |
| D011 | Jiangxi, CHN | 80.00 | 36.25 | 86.49 | 33.11 |
| D091 | Henan, CHN | 55.56 | 25.00 | 93.10 | 31.03 |
| D092 | Henan, CHN | 31.58 | 10.53 | 97.30 | 54.05 |
| D020 | Hebei, CHN | 80.00 | 35.00 | 89.47 | 50.66 |
| B007 | Sichuan, CHN | 40.00 | 11.25 | 70.27 | 18.92 |
| D006 | Hubei, CHN | 70.00 | 43.75 | 89.74 | 53.21 |
| D029 | Xinjiang, CHN | 95.00 | 65.00 | 100.00 | 59.72 |
| L055 | Shanghai, CHN | 83.33 | 45.83 | 100.00 | 85.14 |
| B008 | Hubei, CHN | 83.33 | 30.56 | 82.86 | 35.00 |
| L024 | Hunan, CHN | 90.91 | 47.73 | 97.44 | 67.31 |
| L006 | Guangxi, CHN | 63.64 | 31.82 | 75.68 | 20.95 |
| L117 | Sichuan, CHN | 78.57 | 35.71 | 85.71 | 34.29 |
| L046 | Guangxi, CHN | 83.33 | 33.33 | 51.28 | 32.05 |
| D061 | Jiangsu, CHN | 37.50 | 10.94 | 35.00 | 12.50 |
| L113 | Hubei, CHN | 85.00 | 45.00 | 77.50 | 45.00 |
| B071 | Hubei, CHN | 88.89 | 58.33 | 93.55 | 49.19 |
| L098 | Hubei, CHN | 90.48 | 35.71 | 94.87 | 51.92 |
| L118 | Sichuan, CHN | 83.33 | 34.72 | 100.00 | 43.94 |
| B011 | Hubei, CHN | 100.00 | 53.95 | 77.78 | 36.81 |
| L119 | Hubei, CHN | 88.00 | 50.00 | 85.37 | 42.07 |
| L092 | Liaoning, CHN | 90.91 | 30.68 | 82.35 | 34.68 |
| L029 | Liaoning, CHN | 78.95 | 42.11 | 94.12 | 44.85 |
| L048 | Jiangsu, CHN | 100.00 | 52.78 | 87.88 | 64.39 |
| L060 | Xinjiang, CHN | 83.33 | 40.28 | 82.50 | 56.25 |
| D094 | Shanghai, CHN | 94.12 | 50.00 | 97.22 | 44.44 |
| B012 | Hubei, CHN | 94.12 | 57.35 | 86.11 | 50.69 |
| L066 | Hubei, CHN | 94.74 | 34.21 | 100.00 | 39.50 |
| B051 | Henan, CHN | 73.68 | 43.42 | 85.29 | 32.35 |
| F037 | Jiangsu, CHN | 94.74 | 59.21 | 95.45 | 50.00 |
| L114 | Xinjiang, CHN | 100.00 | 56.25 | 62.50 | 34.38 |
| D086 | Henan, CHN | 50.00 | 16.25 | 92.11 | 37.50 |
| B042 | Henan, CHN | 94.74 | 46.05 | 92.31 | 50.00 |
| D030 | Xinjiang, CHN | 100.00 | 53.75 | 88.10 | 62.50 |
| D070 | Xinjiang, CHN | 75.00 | 37.50 | 53.66 | 29.27 |
| L022 | Xinjiang, CHN | 90.91 | 51.14 | 78.05 | 37.20 |
| B033 | Hebei, CHN | 19.05 | 4.76 | 19.05 | 7.67 |
| L083 | Sichuan, CHN | 100.00 | 61.11 | 94.87 | 50.00 |
| D004 | Hunan, CHN | 100.00 | 69.12 | 97.30 | 64.19 |
| F007 | Jiangsu, CHN | 100.00 | 65.28 | 94.12 | 66.91 |
| L039 | Liaoning, CHN | 83.33 | 51.39 | 78.95 | 49.34 |
| B078 | Jiangsu, CHN | 76.19 | 41.67 | 100.00 | 35.80 |
| L080 | Henan, CHN | 36.84 | 13.16 | 83.33 | 33.33 |
| B024 | Hubei, CHN | 72.73 | 36.36 | 89.74 | 33.97 |
| L104 | Shandong, CHN | 94.74 | 39.47 | 80.49 | 28.66 |
| D058 | Jiangsu, CHN | 78.95 | 23.68 | 62.50 | 27.34 |
| B056 | Henan, CHN | 100.00 | 58.75 | 92.86 | 50.60 |
| D066 | Xinjiang, CHN | 94.44 | 48.61 | 84.21 | 55.92 |
| D104 | Henan, CHN | 95.24 | 58.33 | 100.00 | 60.58 |
| D095 | Shanghai, CHN | 65.00 | 31.25 | 82.86 | 27.86 |
| L020 | Henan, CHN | 100.00 | 54.17 | 100.00 | 55.49 |
| L026 | Henan, CHN | 95.45 | 42.05 | 95.24 | 61.90 |
| L013 | Hubei, CHN | 90.48 | 52.38 | 97.44 | 66.03 |
| L124 | Shanxi, CHN | 100.00 | 52.78 | 94.29 | 49.29 |
| B045 | Jiangsu, CHN | 94.44 | 61.11 | 97.50 | 66.88 |
| F082 | USA | 95.00 | 57.50 | 100.00 | 67.26 |
| B039 | Xinjiang, CHN | 94.12 | 51.47 | 100.00 | 60.26 |
| L070 | Henan, CHN | 100.00 | 56.67 | 100.00 | 52.78 |
| D039 | Shandong, CHN | 86.36 | 34.09 | 80.43 | 50.00 |
| D099 | Henan, CHN | 100.00 | 60.71 | 92.31 | 39.74 |
| B026 | Shandong, CHN | 94.74 | 44.74 | 90.91 | 34.09 |
| D105 | Beijing, CHN | 76.19 | 44.05 | 97.67 | 36.63 |
| B010 | Hubei, CHN | 89.47 | 47.37 | 91.67 | 31.25 |
| D047 | Shaanxi，CHN | 94.44 | 34.72 | 74.36 | 53.85 |
| D040 | Shandong, CHN | 89.47 | 50.00 | 100.00 | 57.81 |
| B066 | Henan, CHN | 73.68 | 34.21 | 89.47 | 38.16 |
| B041 | USA | 63.16 | 23.68 | 94.44 | 29.86 |
| B083 | Jiangsu, CHN | 94.44 | 41.67 | 45.45 | 16.67 |
| B043 | Anhui, CHN | 68.42 | 35.53 | 94.29 | 26.43 |
| B053 | Henan, CHN | 83.33 | 43.06 | 87.50 | 32.50 |
| B077 | Jiangsu, CHN | 84.21 | 46.05 | 34.21 | 13.16 |
| L041 | Shandong, CHN | 78.95 | 28.95 | 45.95 | 22.30 |
| L074 | Chad | 75.00 | 41.25 | 97.22 | 34.72 |
| D087 | Henan, CHN | 76.47 | 41.18 | 92.31 | 26.28 |
| L049 | Liaoning, CHN | 56.25 | 18.75 | 86.11 | 21.53 |
| F102 | The Soviet Union | 78.26 | 39.13 | 100.00 | 79.03 |
| D028 | Gansu, CHN | 87.50 | 39.06 | 95.12 | 42.07 |
| B022 | Shanxi, CHN | 57.89 | 31.58 | 84.85 | 33.33 |
| F014 | Uganda | 33.33 | 12.50 | 33.33 | 9.55 |
| F101 | The Soviet Union | 11.11 | 2.78 | 66.67 | 18.06 |
| F046 | USA | 71.43 | 39.29 | 91.43 | 26.43 |
| F089 | USA | 60.00 | 31.25 | 100.00 | 60.71 |
| F039 | USA | 63.64 | 35.23 | 100.00 | 31.82 |
| F092 | USA | 59.09 | 23.86 | 48.57 | 22.86 |
| F068 | The Soviet Union | 81.25 | 40.63 | 92.11 | 25.66 |
| B064 | Bulgaria | 72.22 | 37.50 | 96.67 | 51.67 |
| D062 | Zhejiang, CHN | 38.10 | 11.90 | 59.46 | 31.08 |
| B031 | Hebei, CHN | 25.00 | 8.75 | 80.00 | 24.29 |
| B023 | Shanxi, CHN | 63.64 | 21.59 | 97.44 | 25.64 |
| F086 | Sudan | 85.00 | 36.25 | 100.00 | 43.13 |
| F032 | USA | 90.00 | 37.50 | 97.50 | 26.25 |
| F099 | The Soviet Union | 75.00 | 35.00 | 45.95 | 15.54 |
| F083 | USA | 86.96 | 43.48 | 92.00 | 32.00 |
| F073 | Australia | 35.00 | 13.75 | 82.05 | 25.64 |
| F028 | France | 54.55 | 23.86 | 94.44 | 34.03 |
| B029 | Henan, CHN | 100.00 | 50.00 | 94.59 | 42.57 |
| B057 | Henan, CHN | 63.16 | 17.11 | 81.40 | 23.84 |
| F047 | USA | 61.11 | 19.44 | 86.49 | 29.05 |
| B016 | Hebei, CHN | 23.53 | 7.35 | 75.00 | 22.92 |
| B028 | Shandong, CHN | 80.00 | 23.75 | 45.45 | 21.97 |
| F031 | USA | 95.45 | 54.55 | 93.55 | 41.94 |
| B082 | Hubei, CHN | 80.95 | 32.14 | 56.25 | 17.19 |
| F070 | USA | 90.91 | 54.55 | 75.00 | 23.13 |
| B063 | Henan, CHN | 86.96 | 45.65 | 62.16 | 17.57 |
| B019 | Shanxi, CHN | 78.26 | 46.74 | 56.10 | 17.68 |
| F081 | USA | 94.74 | 65.79 | 97.50 | 57.50 |
| F072 | Australia | 82.35 | 39.71 | 64.29 | 16.07 |
| F022 | USA | 83.33 | 37.50 | 82.35 | 28.68 |
| B062 | Henan, CHN | 100.00 | 63.04 | 86.49 | 26.35 |
| F094 | The Soviet Union | 66.67 | 21.43 | 69.44 | 44.44 |
| F035 | USA | 90.91 | 44.32 | 76.92 | 21.79 |
| B037 | Hunan, CHN | 83.33 | 31.25 | 70.37 | 27.78 |
| F071 | USA | 95.24 | 61.90 | 95.12 | 50.00 |
| F008 | Pakistan | 100.00 | 47.62 | 100.00 | 45.12 |
| F065 | USA | 57.89 | 22.37 | 30.56 | 7.64 |
| F015 | Uganda | 100.00 | 59.21 | 100.00 | 54.81 |
| F078 | USA | 70.59 | 36.76 | 86.84 | 27.63 |
| B038 | Hunan, CHN | 100.00 | 45.00 | 80.00 | 20.63 |
| F052 | USA | 77.78 | 38.89 | 95.45 | 69.89 |
| B032 | Hebei, CHN | 68.42 | 40.79 | 91.89 | 37.84 |
| L064 | Henan, CHN | 50.00 | 19.44 | 53.49 | 29.07 |
| B046 | Beijing, CHN | 50.00 | 18.75 | 97.62 | 35.12 |
| D055 | Jiangsu, CHN | 40.00 | 13.75 | 35.00 | 16.88 |
| L025 | Henan, CHN | 39.13 | 14.13 | 52.63 | 23.68 |
| B030 | Hebei, CHN | 100.00 | 36.96 | 69.23 | 45.51 |
| L110 | Xinjiang, CHN | 52.94 | 23.53 | 100.00 | 33.75 |
| B058 | Henan, CHN | 89.47 | 46.05 | 78.57 | 25.00 |
| F024 | USA | 100.00 | 54.17 | 88.10 | 36.90 |
| F053 | USA | 85.00 | 43.75 | 95.24 | 54.17 |
| F050 | Australia | 92.31 | 32.69 | 83.78 | 50.00 |
| F045 | USA | 21.43 | 5.36 | 20.00 | 4.20 |
| F104 | Henan, CHN | 64.71 | 27.94 | 82.93 | 37.20 |
| F054 | USA | 34.78 | 10.87 | 33.00 | 9.70 |
| F090 | USA | 90.91 | 40.91 | 100.00 | 48.26 |
| F004 | Pakistan | 50.00 | 27.50 | 41.67 | 14.58 |
| F036 | USA | 17.65 | 7.35 | 53.66 | 18.29 |
| F069 | The Soviet Union | 54.55 | 34.09 | 95.56 | 50.56 |
| F088 | USA | 31.82 | 9.09 | 87.18 | 39.10 |
| F084 | USA | 66.67 | 28.57 | 97.14 | 47.86 |
| F056 | Pakistan | 57.14 | 20.24 | 73.81 | 23.21 |
| F057 | USA | 11.11 | 5.56 | 35.14 | 12.84 |
| D037 | Liaoning, CHN | 100.00 | 66.67 | 80.39 | 65.20 |
| F018 | Turkey | 78.95 | 38.16 | 77.50 | 33.13 |
| F076 | Uzbekistan | 65.00 | 31.25 | 69.05 | 30.95 |
| F077 | Jiangsu, CHN | 13.04 | 5.44 | 52.27 | 18.75 |
| F044 | USA | 54.55 | 29.55 | 67.44 | 33.14 |
| F067 | USA | 66.67 | 28.57 | 50.00 | 18.13 |
| F013 | USA | 62.50 | 25.00 | 54.05 | 36.49 |
| B035 | Jiangsu, CHN | 85.00 | 55.00 | 97.22 | 52.08 |
| F055 | USA | 65.00 | 25.00 | 44.44 | 13.89 |
| F002 | The Soviet Union | 76.47 | 33.82 | 80.00 | 36.67 |
| F023 | USA | 65.00 | 42.50 | 69.70 | 27.27 |
| F025 | Spain | 68.75 | 20.31 | 63.64 | 18.94 |
| L120 | Shanxi, CHN | 25.00 | 6.25 | 54.05 | 18.24 |
| F100 | The Soviet Union | 50.00 | 15.00 | 43.18 | 15.91 |
| F005 | Pakistan | 65.00 | 30.00 | 81.82 | 39.20 |
| F091 | USA | 57.89 | 27.63 | 51.35 | 15.54 |
| F062 | USA | 52.38 | 19.05 | 66.67 | 25.00 |
| L051 | Jiangsu, CHN | 58.82 | 20.59 | 61.54 | 18.59 |
| F040 | USA | 13.04 | 3.26 | 100.00 | 33.11 |
| F048 | USA | 42.86 | 13.10 | 47.37 | 16.45 |
| L004 | The Soviet Union | 55.00 | 31.25 | 80.00 | 58.75 |
| F026 | USA | 36.84 | 13.16 | 77.50 | 41.25 |
| F093 | USA | 88.24 | 29.41 | 74.07 | 31.48 |
| F027 | USA | 61.11 | 20.83 | 86.84 | 53.95 |
| F066 | USA | 47.06 | 19.12 | 81.82 | 46.97 |
| F017 | Mexico | 35.00 | 16.25 | 41.46 | 18.90 |
| F051 | USA | 57.89 | 21.05 | 61.11 | 41.67 |
| F097 | The Soviet Union | 84.21 | 40.79 | 69.77 | 31.40 |
| B061 | Beijing, CHN | 50.00 | 23.61 | 9.76 | 4.88 |
| F060 | USA | 57.89 | 17.11 | 73.81 | 49.40 |
| F087 | The Soviet Union | 50.00 | 14.77 | 15.38 | 5.13 |
| F006 | Pakistan | 66.67 | 40.28 | 25.64 | 7.69 |
| F074 | Brazil | 58.82 | 26.47 | 60.00 | 18.13 |
| D102 | Henan, CHN | 77.27 | 35.23 | 84.21 | 34.21 |
| F020 | USA | 50.00 | 20.00 | 47.06 | 13.97 |
| F034 | Pakistan | 100.00 | 68.75 | 91.43 | 60.71 |
| B040 | Xinjiang, CHN | 83.33 | 34.72 | 83.33 | 47.50 |
| L097 | Japan | 66.67 | 25.00 | 100.00 | 37.50 |
| L096 | Nanjing, CHN | 40.00 | 11.25 | 89.47 | 32.24 |
| F043 | Russia | 73.68 | 26.32 | 70.27 | 29.05 |
| F098 | The Soviet Union | 61.54 | 32.69 | 66.70 | 34.30 |
| L038 | Liaoning, CHN | 23.81 | 9.52 | 36.36 | 15.91 |
| L084 | USA | 68.42 | 31.58 | 29.27 | 15.85 |
| L027 | Sichuan, CHN | 64.71 | 20.59 | 65.79 | 35.53 |
| D034 | Hunan, CHN | 100.00 | 51.56 | 88.37 | 59.88 |
| F003 | The Soviet Union | 100.00 | 52.94 | 74.47 | 50.53 |
| F085 | USA | 61.11 | 38.89 | 84.62 | 55.77 |
| F096 | The Soviet Union | 94.44 | 41.67 | 67.50 | 29.38 |
| B006 | Henan, CHN | 44.44 | 20.83 | 78.57 | 35.71 |
| F064 | The Soviet Union | 89.47 | 42.11 | 74.36 | 19.87 |
| F033 | USA | 54.55 | 27.27 | 75.00 | 21.53 |
| F080 | USA | 100.00 | 46.05 | 83.78 | 30.41 |
| F041 | USA | 80.95 | 40.48 | 77.27 | 26.70 |
| F001 | The Soviet Union | 66.67 | 23.61 | 88.89 | 30.56 |
| F075 | Jiangxi, CHN | 71.43 | 38.10 | 95.00 | 38.13 |
| F095 | USA | 95.24 | 52.38 | 91.67 | 30.56 |
| F030 | USA | 89.47 | 56.58 | 71.43 | 38.10 |
| L076 | Hebei, CHN | 100.00 | 67.19 | 71.43 | 33.33 |
| F029 | Pakistan | 88.89 | 54.17 | 48.65 | 18.24 |
| F049 | Australia | 76.47 | 36.76 | 83.33 | 34.17 |
| F042 | Russia | 76.19 | 29.76 | 72.50 | 23.13 |
| D088 | Henan, CHN | 19.05 | 5.95 | 37.21 | 10.47 |
| B014 | Hebei, CHN | 9.52 | 2.38 | 27.78 | 9.72 |
| F009 | Henan, CHN | 58.82 | 23.53 | 72.22 | 21.53 |
| F012 | USA | 58.82 | 16.18 | 57.80 | 17.20 |
| B002 | Henan, CHN | 73.68 | 26.32 | 75.00 | 27.40 |
| B005 | Henan, CHN | 52.63 | 22.37 | 25.00 | 6.94 |
| L085 | Hunan, CHN | 13.04 | 5.44 | 31.58 | 8.55 |
| L037 | Liaoning, CHN | 61.11 | 29.17 | 66.67 | 25.64 |
| B067 | Henan, CHN | 15.00 | 5.00 | 37.50 | 11.25 |
| F058 | Henan, CHN | 73.68 | 27.63 | 39.47 | 17.11 |
| F061 | USA | 25.00 | 10.94 | 25.00 | 9.03 |
| F038 | Henan, CHN | 33.33 | 9.72 | 55.56 | 17.36 |
| B068 | Henan, CHN | 66.67 | 27.78 | 42.42 | 10.61 |
| B069 | Henan, CHN | 33.33 | 18.06 | 39.47 | 11.84 |
| D083 | Henan, CHN | 20.00 | 7.50 | 34.88 | 12.79 |
| F059 | Henan, CHN | 18.75 | 4.69 | 13.89 | 6.94 |
| B054 | Henan, CHN | 33.33 | 11.11 | 22.50 | 7.50 |
| D056 | Jiangsu, CHN | 59.09 | 34.09 | 97.44 | 53.21 |
| L061 | Anhui, CHN | 85.71 | 46.43 | 70.45 | 57.95 |
| L101 | Henan, CHN | 25.00 | 7.50 | 12.20 | 3.05 |
| F010 | Henan, CHN | 11.11 | 2.78 | 30.56 | 11.81 |
| D054 | Jiangsu, CHN | 52.94 | 16.18 | 34.38 | 21.09 |
| D043 | Shaanxi，CHN | 100.00 | 41.67 | 70.59 | 34.56 |
| B003 | Henan, CHN | 16.67 | 5.56 | 57.89 | 21.71 |
| L107 | USA | 77.78 | 38.89 | 70.73 | 21.95 |
| D060 | Jiangsu, CHN | 95.65 | 51.09 | 95.00 | 52.30 |
| L063 | Shanghai, CHN | 94.44 | 34.72 | 100.00 | 36.80 |
| L106 | Henan, CHN | 90.00 | 53.75 | 72.97 | 43.92 |
| F011 | Henan, CHN | 45.00 | 12.50 | 21.43 | 6.55 |
| D084 | Henan, CHN | 55.00 | 20.00 | 45.00 | 18.75 |
| B001 | Henan, CHN | 6.25 | 1.56 | 24.32 | 6.08 |
| D085 | Henan, CHN | 35.00 | 13.75 | 44.74 | 18.42 |
| F105 | Henan, CHN | 42.11 | 18.42 | 78.95 | 39.47 |
| L116 | Henan, CHN | 25.00 | 8.75 | 36.36 | 22.73 |
| B059 | Henan, CHN | 38.89 | 11.11 | 0.00 | 0.00 |
| L077 | Henan, CHN | 41.18 | 16.18 | 17.65 | 9.56 |
| L078 | Henan, CHN | 11.76 | 4.41 | 9.09 | 4.55 |
| B017 | Hebei, CHN | 16.67 | 5.56 | 21.21 | 8.33 |
| D033 | Xinjiang, CHN | 95.00 | 46.25 | 71.79 | 47.44 |
| L035 | Henan, CHN | 100.00 | 64.29 | 86.67 | 54.17 |
| D051 | Jiangsu, CHN | 60.00 | 35.00 | 97.37 | 31.58 |
| F103 | Uganda | 50.00 | 23.75 | 35.14 | 10.81 |
| L011 | Jiangsu, CHN | 50.00 | 26.79 | 45.95 | 24.32 |
| D001 | Beijing, CHN | 58.82 | 16.18 | 26.47 | 9.56 |
| D103 | Henan, CHN | 46.67 | 13.33 | 40.63 | 28.13 |
| B044 | Jiangsu, CHN | 42.11 | 11.84 | 33.33 | 21.21 |
| L091 | USA | 66.67 | 23.81 | 62.86 | 30.71 |
| D015 | Hebei, CHN | 83.33 | 29.17 | 48.48 | 24.24 |
| F063 | USA | 44.44 | 13.89 | 83.33 | 57.50 |
| L058 | Hebei, CHN | 68.18 | 26.14 | 58.82 | 35.29 |
| L068 | Henan, CHN | 24.00 | 7.00 | 79.49 | 38.46 |
| D074 | Chongqing, CHN | 20.83 | 6.25 | 63.33 | 31.67 |
| L108 | Henan, CHN | 31.82 | 9.09 | 31.43 | 13.57 |
| D018 | Hebei, CHN | 54.55 | 19.32 | 58.33 | 34.72 |
| B004 | Jiangsu, CHN | 70.00 | 31.25 | 76.47 | 26.47 |
| F021 | Australia | 57.89 | 22.37 | 57.58 | 26.52 |
| B074 | Hebei, CHN | 62.50 | 17.19 | 59.38 | 30.47 |
| B018 | Shanxi, CHN | 66.67 | 23.61 | 68.75 | 28.91 |
| B052 | Henan, CHN | 69.57 | 26.09 | 51.52 | 14.39 |
| D078 | Henan, CHN | 90.91 | 40.91 | 58.82 | 32.35 |
| B080 | Henan, CHN | 60.87 | 17.39 | 25.00 | 12.50 |
| D024 | Shanxi, CHN | 45.45 | 14.77 | 26.47 | 16.18 |
| D009 | Jiangxi, CHN | 85.00 | 26.25 | 100.00 | 30.40 |
| B036 | Jiangsu, CHN | 75.00 | 39.06 | 75.00 | 54.17 |
| D053 | Jiangsu, CHN | 95.00 | 37.50 | 31.25 | 15.63 |
| B047 | Henan, CHN | 55.00 | 16.25 | 60.00 | 17.30 |
| B027 | Shandong, CHN | 84.21 | 56.58 | 100.00 | 50.58 |
| D025 | Shanxi, CHN | 95.24 | 35.71 | 75.00 | 32.03 |
| B009 | Hubei, CHN | 100.00 | 37.50 | 85.00 | 43.92 |
| B048 | Henan, CHN | 90.48 | 34.52 | 75.76 | 35.61 |
| D017 | Hebei, CHN | 77.27 | 23.86 | 90.00 | 33.40 |
| B072 | Hubei, CHN | 94.74 | 59.21 | 95.65 | 48.91 |

**Table S4 Gene annotation of candidate genes locating in the introgressed fragment**

| GeneID | Pfam_annotation | GeneID | Pfam_annotation |
| --- | --- | --- | --- |
| GA10G318500.1 | -- | GA10G322200.1 | TIR domain |
| GA10G318600.1 | -- | GA10G322300.1 | -- |
| GA10G318700.1 | Ankyrin repeats (3 copies) | GA10G322400.1 | TIR domain |
| GA10G318800.1 | Ankyrin repeats (3 copies) | GA10G322500.1 | gag-polypeptide of LTR copia-type |
| GA10G318900.1 | -- | GA10G322600.1 | Dirigent-like protein |
| GA10G319000.1 | Microsomal signal peptidase 25 kDa subunit (SPC25) | GA10G322700.1 | Dirigent-like protein |
| GA10G319100.1 | Mitochondrial carrier protein | GA10G322800.1 | gag-polypeptide of LTR copia-type |
| GA10G319200.1 | C2 domain | GA10G322900.1 | Leucine Rich repeats (2 copies) |
| GA10G319300.1 | NB-ARC domain | GA10G323000.1 | NB-ARC domain |
| GA10G319400.1 | NB-ARC domain | GA10G323100.1 | NB-ARC domain |
| GA10G319500.1 | B3 DNA binding domain | GA10G323200.1 | NB-ARC domain |
| GA10G319600.1 | NB-ARC domain | GA10G323300.1 | TIR domain |
| GA10G319700.1 | TIR domain | GA10G323400.1 | NB-ARC domain |
| GA10G319800.1 | Leucine Rich repeats (2 copies) | GA10G323500.1 | NB-ARC domain |
| GA10G319900.1 | NB-ARC domain | GA10G323600.1 | Leucine Rich repeats (2 copies) |
| GA10G320000.1 | Leucine Rich repeats (2 copies) | GA10G323700.1 | TIR domain |
| GA10G320100.1 | TIR domain | GA10G323800.1 | -- |
| GA10G320200.1 | Ankyrin repeats (3 copies) | GA10G323900.1 | TIR domain |
| GA10G320300.1 | Reverse transcriptase (RNA-dependent DNA polymerase) | GA10G324000.1 | NB-ARC domain |
| GA10G320400.1 | TIR domain | GA10G324100.1 | Leucine Rich repeats (2 copies) |
| GA10G320500.1 | NB-ARC domain | GA10G324200.1 | Leucine Rich repeats (2 copies) |
| GA10G320600.1 | NB-ARC domain | GA10G324300.1 | NB-ARC domain |
| GA10G320700.1 | Reverse transcriptase (RNA-dependent DNA polymerase) | GA10G324400.1 | TIR domain |
| GA10G320800.1 | TIR domain | GA10G324500.1 | NB-ARC domain |
| GA10G320900.1 | Leucine Rich repeats (2 copies) | GA10G324600.1 | TIR domain |
| GA10G321000.1 | TIR domain | GA10G324700.1 | Leucine Rich Repeat |
| GA10G321100.1 | Leucine Rich repeats (2 copies) | GA10G324800.1 | Transferase family |
| GA10G321200.1 | NB-ARC domain | GA10G324900.1 | Transferase family |
| GA10G321300.1 | TIR domain | GA10G325000.1 | Homeobox domain |
| GA10G321400.1 | Terpene synthase family, metal binding domain | GA10G325100.1 | JAB1/Mov34/MPN/PAD-1 ubiquitin protease |
| GA10G321500.1 | -- | GA10G325200.1 | Major intrinsic protein |
| GA10G321600.1 | NB-ARC domain | GA10G325300.1 | Major intrinsic protein |
| GA10G321700.1 | NB-ARC domain | GA10G325400.1 | Major intrinsic protein |
| GA10G321800.1 | NB-ARC domain | GA10G325500.1 | Tetratricopeptide repeat |
| GA10G321900.1 | NB-ARC domain | GA10G325600.1 | Hs1pro-1 protein C-terminus |
| GA10G322000.1 | NB-ARC domain | GA10G325700.1 | Ribosomal protein L10 |
| GA10G322100.1 | Integrase core domain | GA10G325800.1 | Syntaxin |

**Table S5 The conserved domain information of *TIR-NBS-LRR* genes**

| Gene | Domain1 | Domain2 | Domain3 | Domain4 | Gene | Domain1 | Domain2 | Domain3 | Domain4 |
| --- | --- | --- | --- | --- | --- | --- | --- | --- | --- |
| GA10G319300.1 | TIR1 |  | NBS-ARC | | GA10G322000.1 | TIR1 | TIR2 | NBS-ARC | |
| GA10G319400.1 | TIR1 |  | NBS-ARC | LRR | GA10G322200.1 | TIR1 |  | NBS-ARC | |
| GA10G319600.1 | TIR1 |  | NBS-ARC | | GA10G322400.1 | TIR1 |  |  | LRR |
| GA10G319700.1 | TIR1 |  |  |  | GA10G322900.1 | | |  | LRR |
| GA10G319800.1 | | |  | LRR | GA10G323000.1 | TIR1 |  | NBS-ARC | LRR |
| GA10G319900.1 | TIR1 |  | NBS-ARC | | GA10G323100.1 | TIR1 |  | NBS-ARC | |
| GA10G320000.1 | | |  | LRR | GA10G323200.1 | TIR1 |  | NBS-ARC | |
| GA10G320100.1 | TIR1 |  |  |  | GA10G323300.1 | TIR1 |  | NBS-ARC | |
| GA10G320400.1 | TIR1 |  | NBS-ARC | | GA10G323400.1 | TIR1 |  | NBS-ARC | |
| GA10G320500.1 | | | NBS-ARC | | GA10G323500.1 | | | NBS-ARC | |
| GA10G320600.1 | TIR1 |  | NBS-ARC | | GA10G323600.1 | | |  | LRR |
| GA10G320800.1 | TIR1 | TIR2 | NBS-ARC | | GA10G323700.1 | TIR1 |  | NBS-ARC | |
| GA10G320900.1 | | |  | LRR | GA10G323900.1 | TIR1 |  |  |  |
| GA10G321000.1 | TIR1 | TIR2 | NBS-ARC | | GA10G324000.1 | | | NBS-ARC | |
| GA10G321100.1 | | | NBS-ARC | LRR | GA10G324100.1 | | |  | LRR |
| GA10G321200.1 | | | NBS-ARC | | GA10G324200.1 | | |  | LRR |
| GA10G321300.1 | TIR1 | TIR2 | NBS-ARC | LRR | GA10G324300.1 | TIR1 |  | NBS-ARC | LRR |
| GA10G321600.1 | TIR1 |  | NBS-ARC | | GA10G324400.1 | TIR1 |  |  |  |
| GA10G321700.1 | TIR1 |  | NBS-ARC | | GA10G324500.1 | | | NBS-ARC | |
| GA10G321800.1 | TIR1 |  | NBS-ARC | | GA10G324600.1 | TIR1 |  | NBS-ARC | |
| GA10G321900.1 | TIR1 |  | NBS-ARC | | GA10G324700.1 | | |  | LRR |

**Table S6 Gene expression profile of an introgressed line under 9- and 72-hour post inoculation of *V. dahliae****.* R1, R2 and R3 represent the three biological replicates.

| Gene ID | Water treatment (hpi) | | | | | | | | | | *V. dahliae* treatment (hpi) | | | | | | | | | | | | |
| --- | --- | --- | --- | --- | --- | --- | --- | --- | --- | --- | --- | --- | --- | --- | --- | --- | --- | --- | --- | --- | --- | --- | --- |
|  | 9-R1 | 9-R2 | | 9-R3 | 72-R1 | | 72-R2 | | 72-R3 | | 9-R1 | | 9-R2 | | 9-R3 | | 72-R1 | | 72-R2 | | | 72-R3 | |
| GA10G321300(CG01) | 2.7 | 2.6 | 2.0 | | | 3.0 | | 3.4 | | 3.8 | | 4.7 | | 5.2 | | 5.7 | | 2.7 | | 2.9 | 3.5 | |  |
| GA10G324900(CG02) | 3.1 | 2.5 | 3.7 | | | 7.1 | | 5.2 | | 6.2 | | 6.0 | | 7.4 | | 8.8 | | 7.3 | | 7.6 | 7.9 | |  |
| GA10G319200(CG03) | 8.7 | 10.0 | 7.4 | | | 8.0 | | 8.3 | | 8.2 | | 36.6 | | 46.9 | | 57.2 | | 5.8 | | 5.2 | 4.7 | |  |
| GA10G320100(CG04 | 0.2 | 0.2 | 0.3 | | | 0.2 | | 0.5 | | 0.3 | | 3.1 | | 2.9 | | 2.7 | | 0.1 | | 0.1 | 0.2 | |  |
| GA10G320200(CG05 | 0.1 | 0.1 | 0.2 | | | 0.0 | | 0.0 | | 0.0 | | 4.0 | | 5.3 | | 6.6 | | 0.0 | | 0.0 | 0.0 | |  |
| GA10G318600(CG06) | 0.0 | 0.0 | 0.0 | | | 0.0 | | 0.0 | | 0.0 | | 14.9 | | 16.2 | | 17.5 | | 0.0 | | 0.0 | 0.0 | |  |
| GA10G323200(CG07) | 0.0 | 0.0 | 0.0 | | | 0.0 | | 0.0 | | 0.0 | | 2.5 | | 2.8 | | 3.2 | | 0.0 | | 0.0 | 0.0 | |  |
| GA10G324400(CG08) | 0.0 | 0.0 | 0.0 | | | 0.0 | | 0.0 | | 0.0 | | 5.7 | | 6.6 | | 7.6 | | 0.0 | | 0.0 | 0.0 | |  |
| GA10G320500(CG09 | 0.0 | 0.0 | 0.0 | | | 0.0 | | 0.0 | | 0.0 | | 1.4 | | 2.2 | | 3.0 | | 0.0 | | 0.0 | 0.1 | |  |
| GA10G321700(CG1) | 5.4 | 5.3 | 8.1 | | | 14.8 | | 14.4 | | 14.6 | | 4.5 | | 4.2 | | 4.1 | | 4.8 | | 6.6 | 8.1 | |  |
| GA10G325400(CG11) | 332.1 | 387.7 | 298.0 | | | 395.8 | | 378.4 | | 346.1 | | 298.0 | | 333.1 | | 318.9 | | 168.9 | | 186.5 | 204.2 | |  |
| GA10G319000(CG12) | 11.2 | 12.2 | 10.2 | | | 12.6 | | 8.6 | | 15.6 | | 15.2 | | 14.8 | | 19.2 | | 26.3 | | 28.3 | 23.4 | |  |
| GA10G325600(CG13) | 10.4 | 10.8 | 12.0 | | | 13.7 | | 16.1 | | 14.9 | | 18.4 | | 16.2 | | 14.0 | | 63.6 | | 57.7 | 51.9 | |  |
| GA10G318500 | 23.2 | 21.3 | 22.0 | | | 25.5 | | 23.5 | | 25.0 | | 24.2 | | 21.4 | | 25.2 | | 18.0 | | 18.9 | 19.9 | |  |
| GA10G325200 | 386.5 | 422.7 | 359.4 | | | 530.7 | | 492.6 | | 460.2 | | 285.8 | | 281.3 | | 340.1 | | 220.4 | | 382.8 | 260.6 | |  |
| GA10G325300 | 95.8 | 97.8 | 93.7 | | | 214.2 | | 194.2 | | 204.2 | | 45.7 | | 59.7 | | 40.2 | | 137.3 | | 127.8 | 137.3 | |  |
| GA10G325700 | 139.8 | 118.1 | 112.5 | | | 70.8 | | 56.1 | | 70.7 | | 116.5 | | 137.7 | | 121.6 | | 68.8 | | 71.9 | 75.1 | |  |
| GA10G318900 | 45.6 | 47.5 | 57.3 | | | 85.3 | | 78.2 | | 65.8 | | 35.6 | | 34.4 | | 33.2 | | 52.7 | | 48.9 | 48.6 | |  |
| GA10G325100 | 20.9 | 20.0 | 18.5 | | | 15.7 | | 14.4 | | 14.0 | | 15.8 | | 19.4 | | 15.9 | | 18.4 | | 20.3 | 17.5 | |  |
| GA10G319100 | 7.2 | 7.6 | 6.9 | | | 8.1 | | 7.6 | | 7.9 | | 10.9 | | 11.0 | | 13.9 | | 11.2 | | 15.2 | 13.2 | |  |
| GA10G321800 | 4.2 | 4.2 | 5.3 | | | 10.0 | | 8.5 | | 6.8 | | 4.6 | | 3.0 | | 4.1 | | 5.2 | | 5.7 | 5.3 | |  |
| GA10G325800 | 6.2 | 5.0 | 5.2 | | | 4.9 | | 5.0 | | 5.0 | | 5.3 | | 5.8 | | 7.7 | | 4.6 | | 5.6 | 3.5 | |  |
| GA10G324800 | 4.4 | 3.8 | 3.2 | | | 5.1 | | 3.6 | | 4.3 | | 3.4 | | 5.1 | | 6.9 | | 5.7 | | 6.6 | 4.7 | |  |
| GA10G323100 | 3.6 | 4.1 | 3.1 | | | 4.7 | | 4.0 | | 4.4 | | 2.8 | | 2.8 | | 2.8 | | 2.5 | | 2.0 | 2.7 | |  |
| GA10G323400 | 2.1 | 2.6 | 2.6 | | | 3.3 | | 3.7 | | 3.3 | | 2.2 | | 2.4 | | 2.4 | | 2.0 | | 2.5 | 2.3 | |  |
| GA10G320600 | 1.0 | 0.0 | 0.0 | | | 0.0 | | 0.0 | | 0.0 | | 1.4 | | 0.2 | | 2.9 | | 0.0 | | 0.0 | 0.0 | |  |
| GA10G323500 | 1.7 | 1.5 | 2.2 | | | 2.9 | | 2.7 | | 2.8 | | 1.1 | | 1.9 | | 1.1 | | 1.3 | | 1.5 | 2.1 | |  |
| GA10G325000 | 1.6 | 2.5 | 2.1 | | | 1.2 | | 1.3 | | 1.1 | | 1.5 | | 1.3 | | 2.1 | | 0.9 | | 1.1 | 1.3 | |  |
| GA10G321600 | 0.3 | 0.4 | 0.8 | | | 0.6 | | 0.7 | | 0.6 | | 0.4 | | 0.6 | | 0.3 | | 2.5 | | 1.2 | 2.5 | |  |
| GA10G324300 | 1.7 | 1.7 | 1.8 | | | 1.9 | | 1.9 | | 2.4 | | 1.8 | | 1.7 | | 2.0 | | 2.0 | | 2.1 | 2.3 | |  |
| GA10G323700 | 0.5 | 0.4 | 0.7 | | | 0.6 | | 0.6 | | 0.6 | | 1.7 | | 1.6 | | 1.4 | | 0.2 | | 0.1 | 0.0 | |  |
| GA10G323900 | 0.7 | 0.8 | 0.6 | | | 0.9 | | 1.0 | | 0.9 | | 1.4 | | 0.5 | | 1.7 | | 0.5 | | 0.5 | 0.6 | |  |
| GA10G323600 | 0.0 | 0.0 | 0.0 | | | 0.0 | | 0.0 | | 0.0 | | 1.2 | | 1.2 | | 1.1 | | 1.7 | | 1.5 | 1.6 | |  |
| GA10G324200 | 1.0 | 0.6 | 0.3 | | | 0.6 | | 0.4 | | 0.5 | | 1.6 | | 0.4 | | 1.0 | | 0.1 | | 0.2 | 0.2 | |  |
| GA10G320800 | 0.0 | 0.0 | 0.1 | | | 0.1 | | 0.1 | | 0.8 | | 0.9 | | 1.2 | | 1.4 | | 0.0 | | 0.7 | 0.1 | |  |
| GA10G324000 | 0.7 | 0.1 | 0.2 | | | 0.2 | | 0.0 | | 0.1 | | 1.2 | | 1.3 | | 1.3 | | 0.1 | | 0.1 | 0.0 | |  |
| GA10G324600 | 0.0 | 0.0 | 0.0 | | | 0.0 | | 0.0 | | 0.0 | | 1.3 | | 1.2 | | 1.2 | | 0.1 | | 0.1 | 0.0 | |  |
| GA10G320000 | 0.1 | 0.0 | 0.1 | | | 0.1 | | 0.1 | | 0.1 | | 0.0 | | 0.2 | | 0.0 | | 1.0 | | 0.2 | 0.4 | |  |
| GA10G318700 | 0.8 | 0.6 | 0.9 | | | 0.5 | | 0.3 | | 0.5 | | 0.6 | | 0.8 | | 0.5 | | 0.7 | | 0.4 | 0.3 | |  |
| GA10G321000 | 0.4 | 0.3 | 0.9 | | | 0.7 | | 0.6 | | 0.6 | | 0.5 | | 0.3 | | 0.6 | | 0.8 | | 0.3 | 0.7 | |  |
| GA10G323800 | 0.9 | 0.0 | 0.3 | | | 0.2 | | 0.1 | | 0.7 | | 0.9 | | 0.0 | | 0.7 | | 0.0 | | 0.6 | 0.0 | |  |
| GA10G318800 | 0.5 | 0.0 | 0.2 | | | 0.1 | | 0.0 | | 0.1 | | 0.9 | | 0.5 | | 0.7 | | 0.0 | | 0.0 | 0.0 | |  |
| GA10G323000 | 0.6 | 0.5 | 0.4 | | | 0.3 | | 0.4 | | 0.2 | | 0.8 | | 0.8 | | 0.8 | | 0.5 | | 0.5 | 0.4 | |  |
| GA10G324100 | 0.8 | 0.1 | 0.2 | | | 0.1 | | 0.2 | | 0.1 | | 0.7 | | 0.4 | | 0.5 | | 0.1 | | 0.7 | 0.1 | |  |
| GA10G321200 | 0.5 | 0.2 | 0.6 | | | 0.4 | | 0.7 | | 0.5 | | 0.3 | | 0.3 | | 0.4 | | 0.6 | | 0.3 | 0.7 | |  |
| GA10G322000 | 0.1 | 0.3 | 0.1 | | | 0.5 | | 0.6 | | 0.1 | | 0.1 | | 0.2 | | 0.0 | | 0.2 | | 0.0 | 0.1 | |  |
| GA10G319300 | 0.2 | 0.0 | 0.0 | | | 0.0 | | 0.0 | | 0.2 | | 0.2 | | 0.1 | | 0.6 | | 0.0 | | 0.4 | 0.0 | |  |
| GA10G320400 | 0.0 | 0.0 | 0.0 | | | 0.0 | | 0.0 | | 0.0 | | 0.0 | | 0.0 | | 0.0 | | 0.5 | | 0.0 | 0.0 | |  |
| GA10G321900 | 0.2 | 0.0 | 0.0 | | | 0.0 | | 0.0 | | 0.4 | | 0.5 | | 0.1 | | 0.4 | | 0.0 | | 0.0 | 0.0 | |  |
| GA10G322700 | 0.0 | 0.2 | 0.0 | | | 0.2 | | 0.0 | | 0.0 | | 0.5 | | 0.4 | | 0.4 | | 0.1 | | 0.1 | 0.0 | |  |
| GA10G319700 | 0.0 | 0.0 | 0.0 | | | 0.1 | | 0.4 | | 0.0 | | 0.0 | | 0.3 | | 0.0 | | 0.3 | | 0.1 | 0.4 | |  |
| GA10G322600 | 0.0 | 0.0 | 0.0 | | | 0.0 | | 0.0 | | 0.0 | | 0.1 | | 0.0 | | 0.4 | | 0.0 | | 0.0 | 0.0 | |  |
| GA10G324700 | 0.2 | 0.0 | 0.0 | | | 0.0 | | 0.0 | | 0.2 | | 0.3 | | 0.0 | | 0.3 | | 0.0 | | 0.1 | 0.0 | |  |
| GA10G320900 | 0.1 | 0.0 | 0.0 | | | 0.0 | | 0.0 | | 0.2 | | 0.1 | | 0.0 | | 0.2 | | 0.0 | | 0.1 | 0.0 | |  |
| GA10G322200 | 0.1 | 0.1 | 0.1 | | | 0.1 | | 0.2 | | 0.2 | | 0.2 | | 0.1 | | 0.2 | | 0.1 | | 0.1 | 0.1 | |  |
| GA10G319400 | 0.0 | 0.0 | 0.0 | | | 0.0 | | 0.0 | | 0.1 | | 0.1 | | 0.0 | | 0.2 | | 0.0 | | 0.2 | 0.0 | |  |
| GA10G323300 | 0.0 | 0.1 | 0.0 | | | 0.1 | | 0.0 | | 0.1 | | 0.1 | | 0.0 | | 0.2 | | 0.0 | | 0.0 | 0.0 | |  |
| GA10G319500 | 0.1 | 0.0 | 0.0 | | | 0.0 | | 0.0 | | 0.1 | | 0.0 | | 0.0 | | 0.2 | | 0.0 | | 0.0 | 0.0 | |  |
| GA10G321100 | 0.1 | 0.0 | 0.1 | | | 0.0 | | 0.1 | | 0.1 | | 0.0 | | 0.0 | | 0.1 | | 0.0 | | 0.1 | 0.0 | |  |
| GA10G321400 | 0.1 | 0.0 | 0.0 | | | 0.1 | | 0.0 | | 0.0 | | 0.0 | | 0.0 | | 0.0 | | 0.1 | | 0.0 | 0.0 | |  |
| GA10G324500 | 0.0 | 0.0 | 0.0 | | | 0.0 | | 0.0 | | 0.0 | | 0.1 | | 0.0 | | 0.1 | | 0.0 | | 0.1 | 0.0 | |  |
| GA10G322400(CG14) | 0.0 | 0.0 | 0.0 | | | 0.1 | | 0.0 | | 0.0 | | 0.0 | | 0.0 | | 0.0 | | 0.0 | | 0.0 | 0.0 | |  |
| GA10G325500 | 0.0 | 0.0 | 0.0 | | | 0.0 | | 0.0 | | 0.0 | | 0.0 | | 0.0 | | 0.0 | | 0.0 | | 0.1 | 0.0 | |  |
| GA10G322900 | 0.0 | 0.0 | 0.0 | | | 0.0 | | 0.0 | | 0.0 | | 0.0 | | 0.0 | | 0.0 | | 0.0 | | 0.0 | 0.0 | |  |
| GA10G319900 | 0.0 | 0.0 | 0.0 | | | 0.0 | | 0.0 | | 0.0 | | 0.0 | | 0.0 | | 0.0 | | 0.0 | | 0.0 | 0.0 | |  |
| GA10G319600 | 0.0 | 0.0 | 0.0 | | | 0.0 | | 0.0 | | 0.0 | | 0.0 | | 0.0 | | 0.0 | | 0.0 | | 0.0 | 0.0 | |  |
| GA10G319800 | 0.0 | 0.0 | 0.0 | | | 0.0 | | 0.0 | | 0.0 | | 0.0 | | 0.0 | | 0.0 | | 0.0 | | 0.0 | 0.0 | |  |
| GA10G320300 | 0.0 | 0.0 | 0.0 | | | 0.0 | | 0.0 | | 0.0 | | 0.0 | | 0.0 | | 0.0 | | 0.0 | | 0.0 | 0.0 | |  |
| GA10G320700 | 0.0 | 0.0 | 0.0 | | | 0.0 | | 0.0 | | 0.0 | | 0.0 | | 0.0 | | 0.0 | | 0.0 | | 0.0 | 0.0 | |  |
| GA10G321500 | 0.0 | 0.0 | 0.0 | | | 0.0 | | 0.0 | | 0.0 | | 0.0 | | 0.0 | | 0.0 | | 0.0 | | 0.0 | 0.0 | |  |
| GA10G322100 | 0.0 | 0.0 | 0.0 | | | 0.0 | | 0.0 | | 0.0 | | 0.0 | | 0.0 | | 0.0 | | 0.0 | | 0.0 | 0.0 | |  |
| GA10G322300 | 0.0 | 0.0 | 0.0 | | | 0.0 | | 0.0 | | 0.0 | | 0.0 | | 0.0 | | 0.0 | | 0.0 | | 0.0 | 0.0 | |  |
| GA10G322500 | 0.0 | 0.0 | 0.0 | | | 0.0 | | 0.0 | | 0.0 | | 0.0 | | 0.0 | | 0.0 | | 0.0 | | 0.0 | 0.0 | |  |
| GA10G322800 | 0.0 | 0.0 | 0.0 | | | 0.0 | | 0.0 | | 0.0 | | 0.0 | | 0.0 | | 0.0 | | 0.0 | | 0.0 | 0.0 | |  |

**Table S7 Result of Systematic Y2H library screening sequencing**

| ID | identity | Target gene ID | Annotion |
| --- | --- | --- | --- |
| Clone-1 | 511/634 (80.6%) | Gh_A01G189300.1 | Pre-mRNA-splicing factor |
| Clone-2 | 769/769 (100%) | Gh_D08G195200.1 | Peptidyl-prolyl cis-trans isomerase FKBP17-2 |
| Clone-3 | 747/747 (100%) | Gh_D08G186200.1 | NA |
| Clone-4 | NA | NA | NA |
| Clone-5 | 851/851 (100%) | Gh_A08G198800.1 | Peptidyl-prolyl cis-trans isomerase FKBP17-2 |
| Clone-6 | NA | NA | NA |
| Clone-7 | NA | NA | NA |
| Clone-8 | NA | NA | NA |
| Clone-9 | NA | NA | NA |
| Clone-10 | NA | NA | NA |
| Clone-11 | 811/811 (100%) | Gh_D10G041000.1 | Urease accessory protein G |
| Clone-12 | NA | NA | NA |
| Clone-13 | NA | NA | NA |
| Clone-14 | NA | NA | NA |
| Clone-15 | 292/364 (80.2%) | Gh_D05G217200.1 | Glutamyl-tRNA reductase 1 |
| Clone-16 | 772/772 (100%), | Gh_D08G195200.1 | Peptidyl-prolyl cis-trans isomerase FKBP17-2 |
| Clone-17 | NA | NA | NA |
| Clone-18 | 247/311 (79.4%) | Gh_D09G238300.1 | Auxin-induced protein 22D |
| Clone-19 | 769/769 (100%) | Gh_D08G195200.1 | Peptidyl-prolyl cis-trans isomerase FKBP17-2 |
| Clone-20 | NA | NA | NA |
| Clone-21 | NA | NA | NA |
| Clone-22 | NA | NA | NA |
| Clone-23 | 75/90 (83.3%) | Gh_A06G029200.1 | Amino acid permease 6 |
| Clone-24 | NA | NA | NA |

**Table S8 Exotic introgression analysis of G. hirsutum on chromosome A10**

| Taxa ID | donor for A10 introgressions | start (A10) | end (A10) |
| --- | --- | --- | --- |
| B001 | *G. arboreum* | 112700451 | 113746497 |
| B005 | *G. arboreum* | 112700451 | 113746497 |
| B014 | *G. arboreum* | 112700451 | 113979872 |
| B017 | *G. arboreum* | 112700451 | 113979872 |
| B027 | *G. arboreum* | 112700451 | 113746497 |
| B031 | *G. arboreum* | 112700451 | 113979872 |
| B033 | *G. arboreum* | 112700451 | 113979872 |
| B050 | *G. arboreum* | 112700451 | 113979872 |
| B053 | *G. arboreum* | 112700451 | 113746497 |
| B059 | *G. arboreum* | 112700451 | 113746497 |
| B061 | *G. arboreum* | 112700451 | 113979872 |
| B066 | *G. arboreum* | 112700451 | 113746497 |
| B067 | *G. arboreum* | 112700451 | 113746497 |
| D012 | *G. arboreum* | 113645230 | 113979872 |
| D076 | *G. arboreum* | 112700451 | 113979872 |
| D084 | *G. arboreum* | 112700451 | 113746497 |
| D085 | *G. arboreum* | 112700451 | 113746497 |
| F006 | *G. arboreum* | 112700451 | 114792261 |
| F014 | *G. arboreum* | 112700451 | 113979872 |
| F017 | *G. arboreum* | 112700451 | 113746497 |
| F033 | *G. arboreum* | 112700451 | 114792261 |
| F038 | *G. arboreum* | 112700451 | 113979872 |
| F045 | *G. arboreum* | 112700451 | 113746497 |
| F050 | *G. arboreum* | 112700451 | 113979872 |
| F054 | *G. arboreum* | 112700451 | 113746497 |
| F074 | *G. arboreum* | 112700451 | 113979872 |
| F079 | *G. arboreum* | 112700451 | 114792261 |
| F086 | *G. arboreum* | 112700451 | 113979872 |
| L025 | *G. arboreum* | 112700451 | 113746497 |
| L060 | *G. arboreum* | 112700451 | 113746497 |
| L074 | *G. arboreum* | 112700451 | 114139556 |
| L084 | *G. arboreum* | 112700451 | 114792261 |
| L103 | *G. arboreum* | 112700451 | 113979872 |
| L104 | *G. arboreum* | 112700451 | 112821023 |
| L116 | *G. arboreum* | 112700451 | 113979872 |
| L122 | *G. arboreum* | 112700451 | 113746497 |
| B014 | *G. barbadense* | 110101654 | 110717328 |
| D083 | *G. barbadense* | 236 | 90981 |
| D083 | *G. barbadense* | 383034 | 3120387 |
| F022 | *G. barbadense* | 110009439 | 110048511 |
| F057 | *G. barbadense* | 81494810 | 81553422 |
| F099 | *G. barbadense* | 105262663 | 106855890 |
| L045 | *G. barbadense* | 113762756 | 113964604 |
| L104 | *G. barbadense* | 109959059 | 111401217 |
| L107 | *G. barbadense* | 12144359 | 12654533 |
