# Supplementary material for: Genome-wide association analysis reveals a novel pathway mediated by a dual-TIR domain protein for pathogen resistance in cotton
Source: Genome Biol. 2023 May 10;24:111. doi: 10.1186/s13059-023-02950-9 (PMC10170703; doi:10.1186/s13059-023-02950-9)
Supplement: Supplementary file 3 — Additional file 3. Uncropped western blot images related to Figs. 3, 4, 5 and 6 and Additional file 1: Fig. S5. [file 13059_2023_2950_MOESM3_ESM.docx]

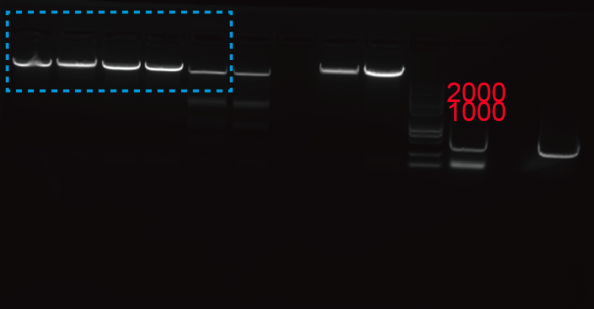


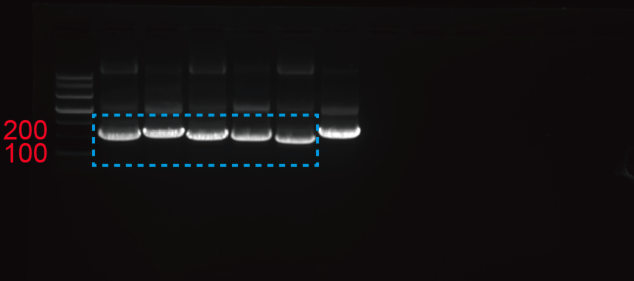


Fig. 3c Transcript detection of both GhRVD1 genotypes using semi-quantitative PCR with universal primers. GhHiston3 was used as an internal control

Anti-Actin


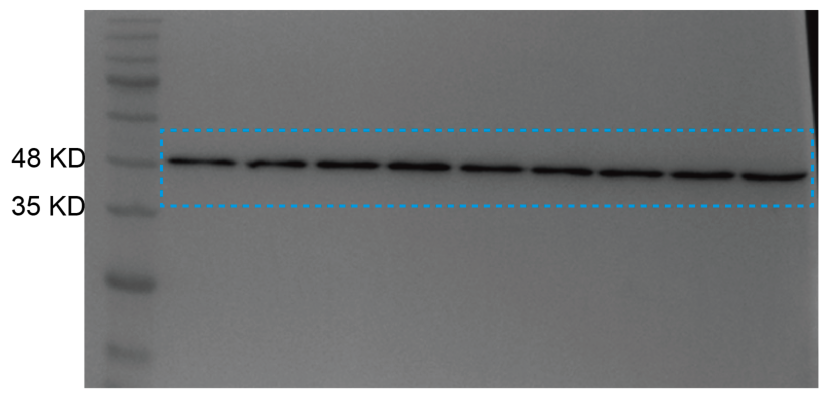


Anti-Flag


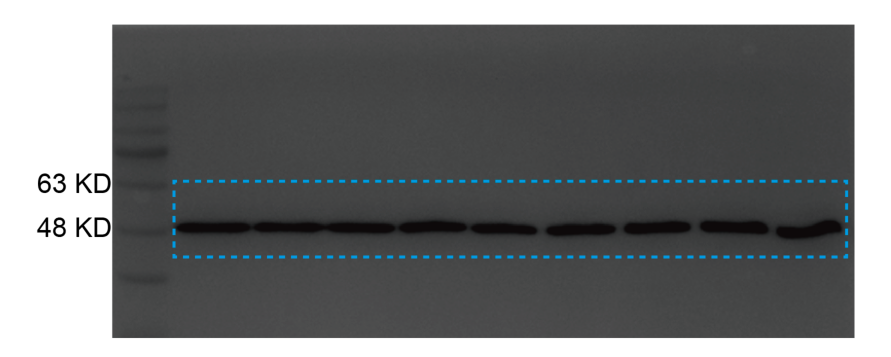


Fig. 4c Western blots of TIR1-TIR2 variants using a flag antibody, and immunoblotting of plant actin with α-actin was used as a loading control.

Anti-Falg


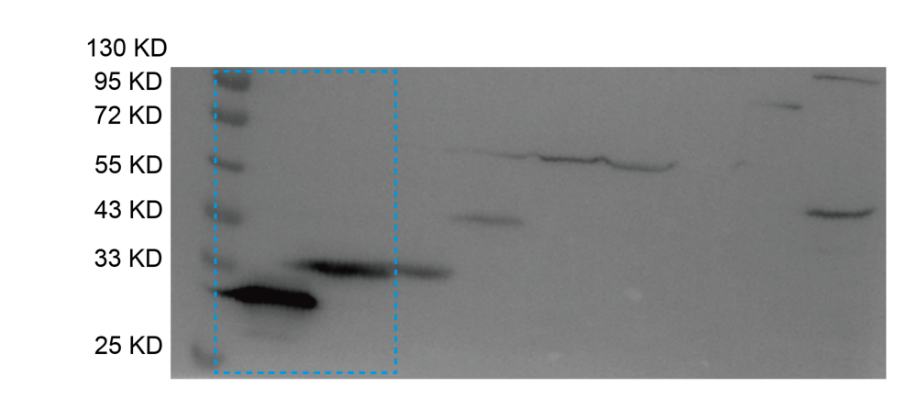


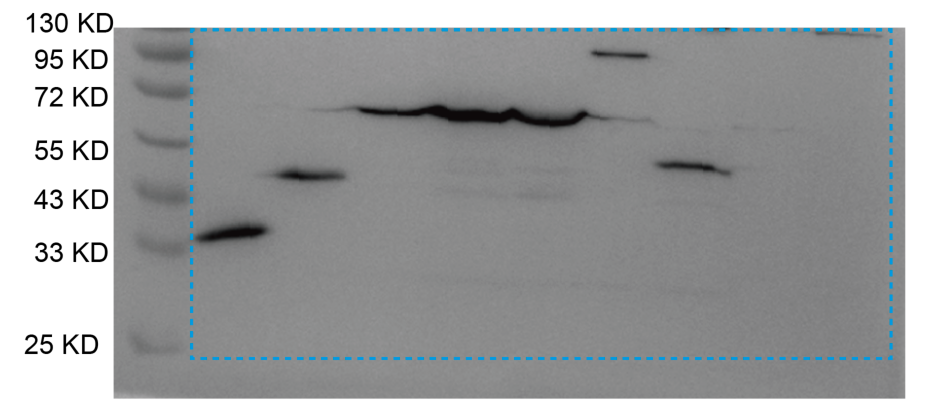


Anti-Actin


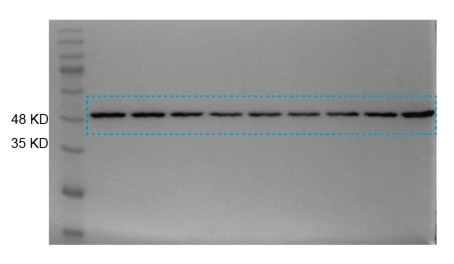


Fig. 4j Western blots of truncated derivatives of GhRVD1_R using a flag antibody, and immunoblotting of plant actin with α-actin was used as a loading control.

Anti-Flag


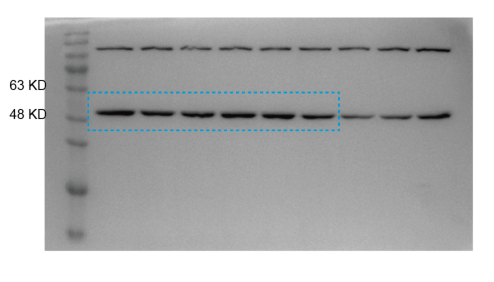


Anti-Actin


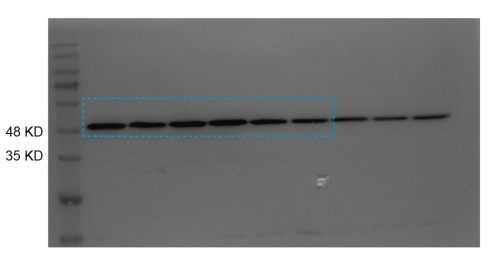


Fig. 5b Western blots of HR proteins using a flag antibody, and immunoblotting of plant actin with α-actin was used as a loading control.

Anti-HA


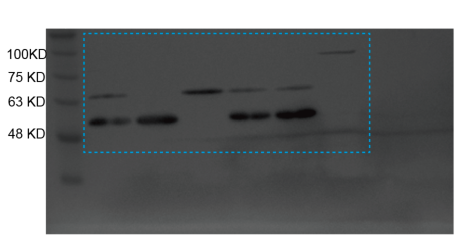


Anti-Actin


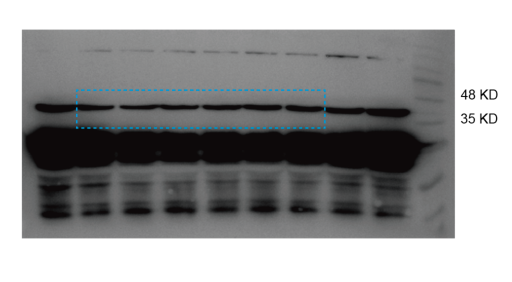


Fig. 5f Western blots of HR proteins using a HA antibody, and immunoblotting of plant actin with α-actin was used as a loading control.


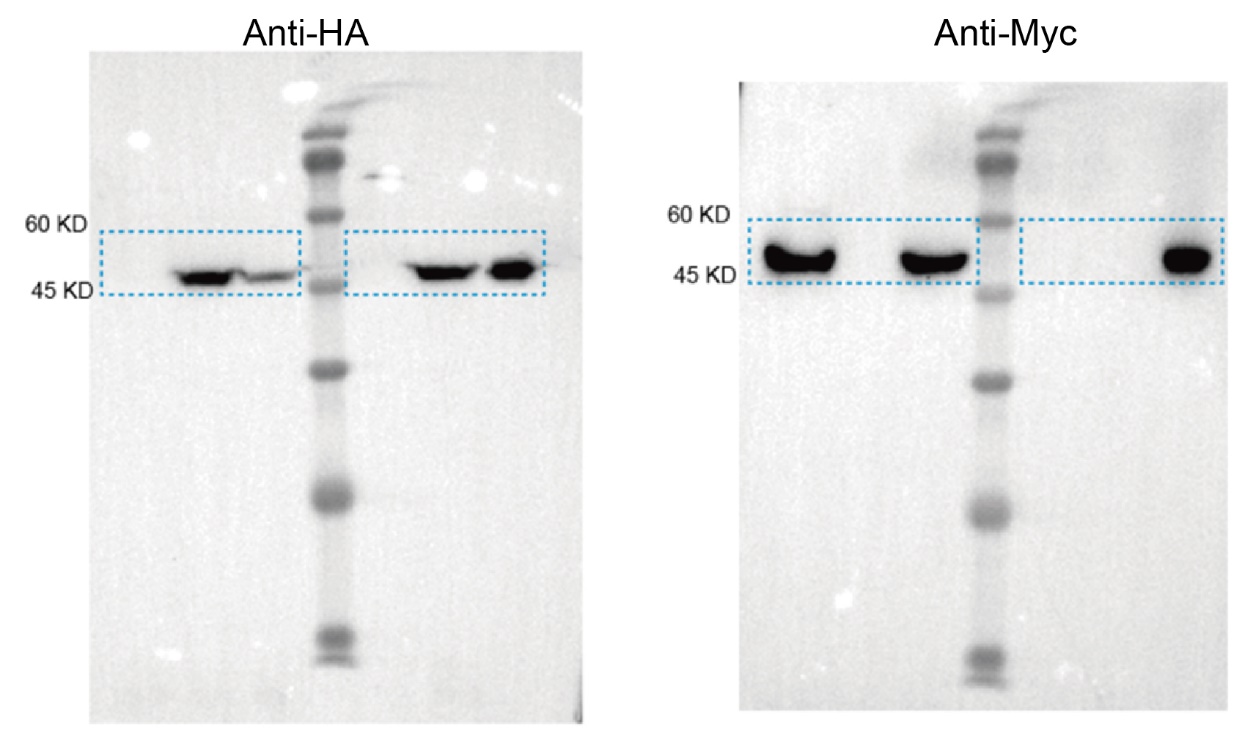


Fig. 5d-1 Coimmunoprecipitation analysis of self-association of TIR1-TIR2_R


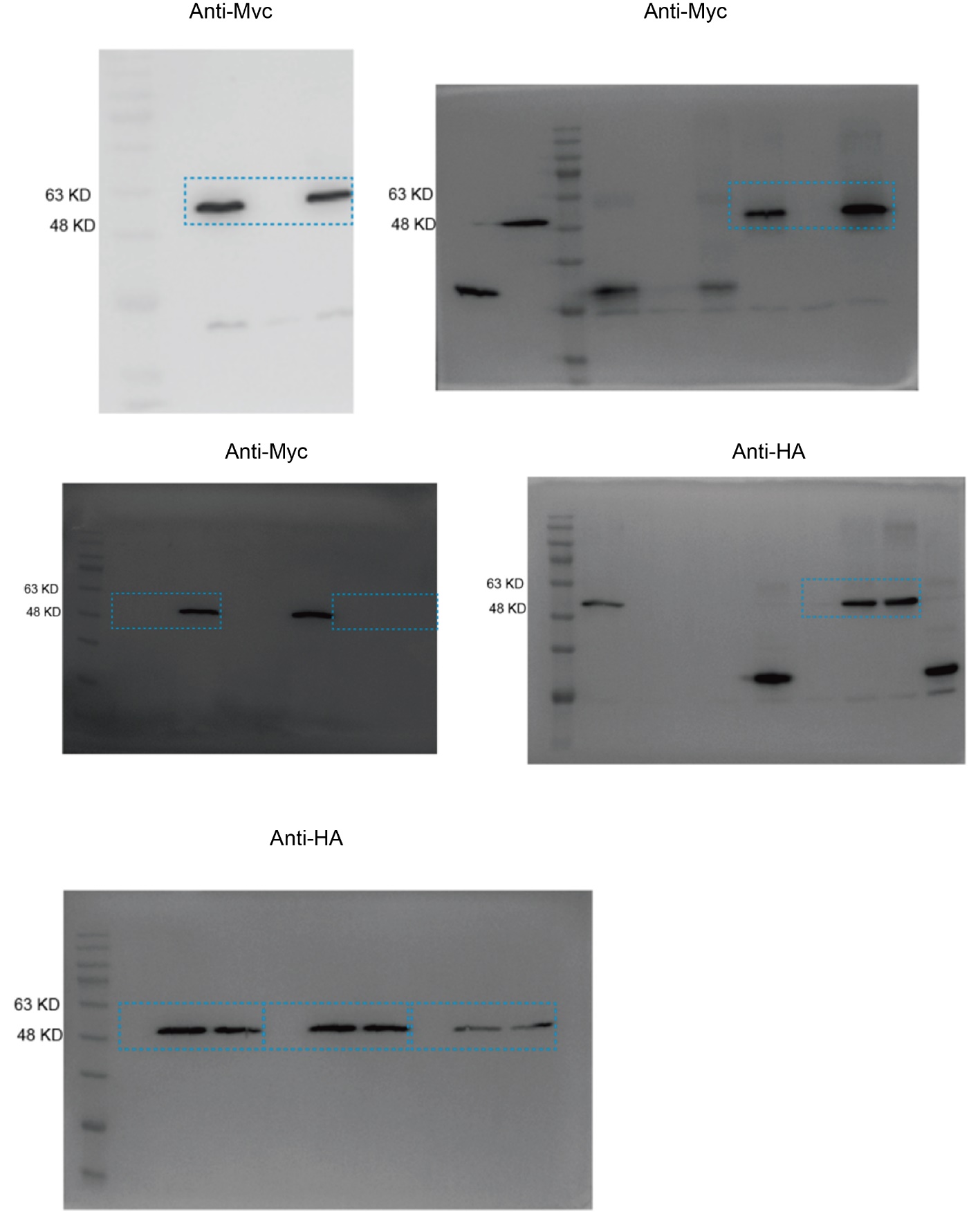


Fig. 5d-2 Coimmunoprecipitation analysis of self-association of TIR1-TIR2_R variants


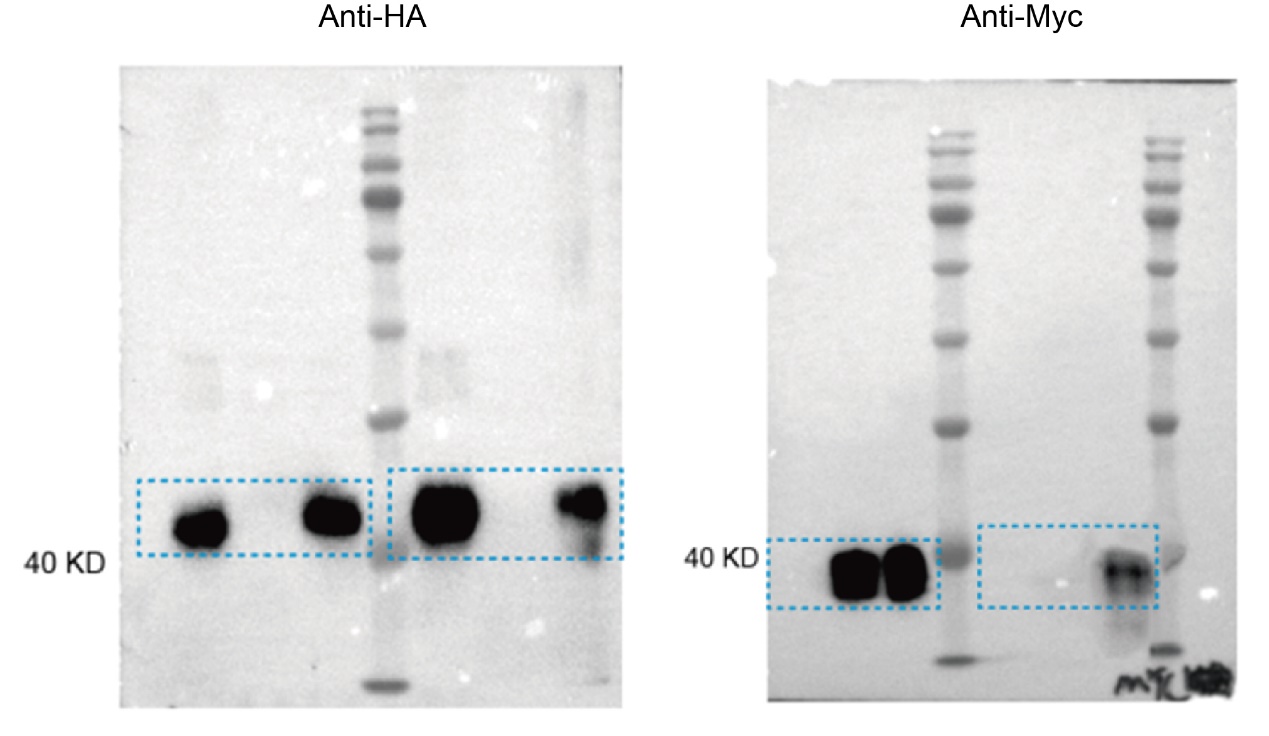


Fig. 5i-1 Coimmunoprecipitation analysis of the interaction between TIR1L_R and TIR2L_R


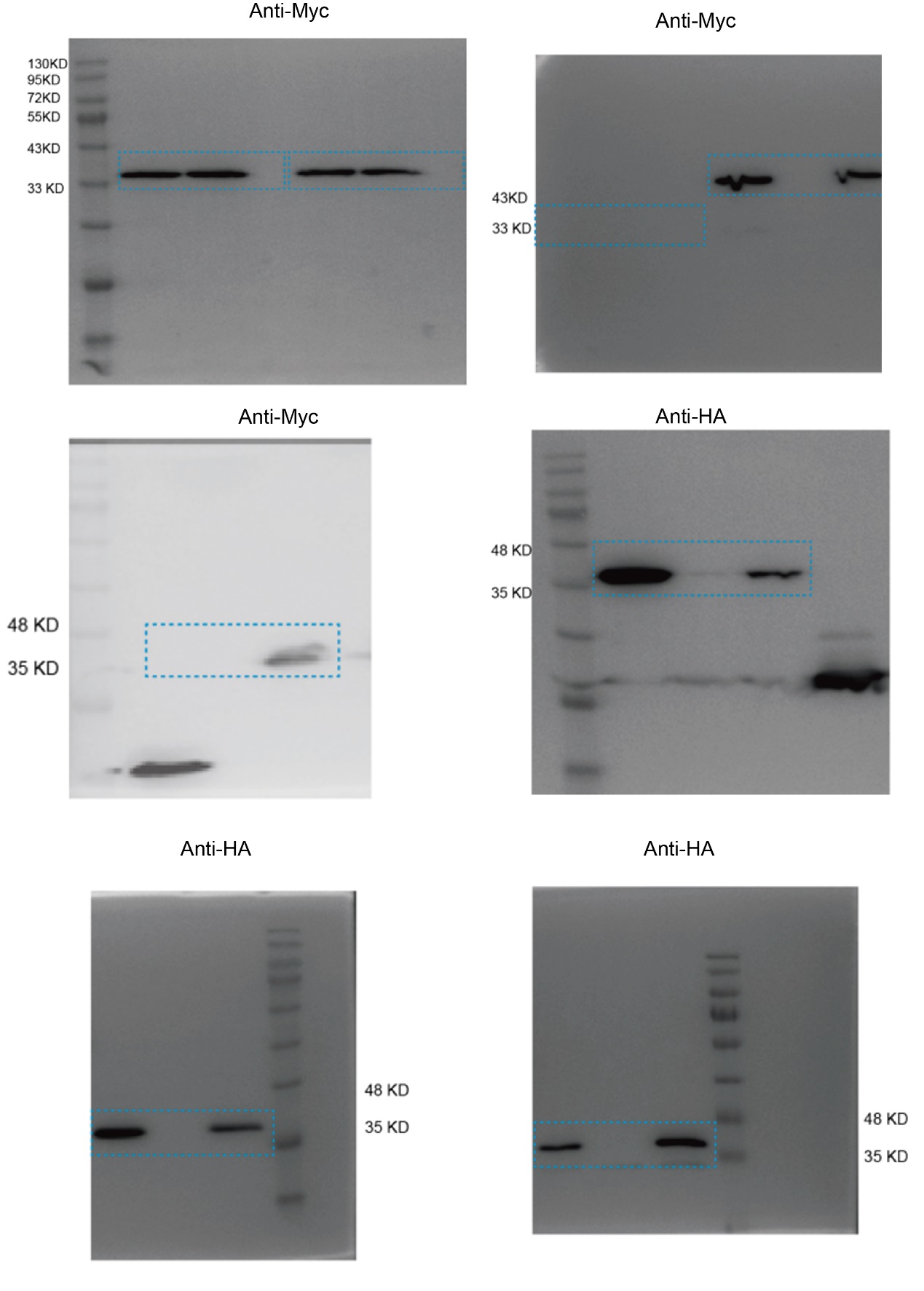


Fig. 5i-2 Coimmunoprecipitation analysis of the interaction between TIR1L_R and TIR2L_R variants


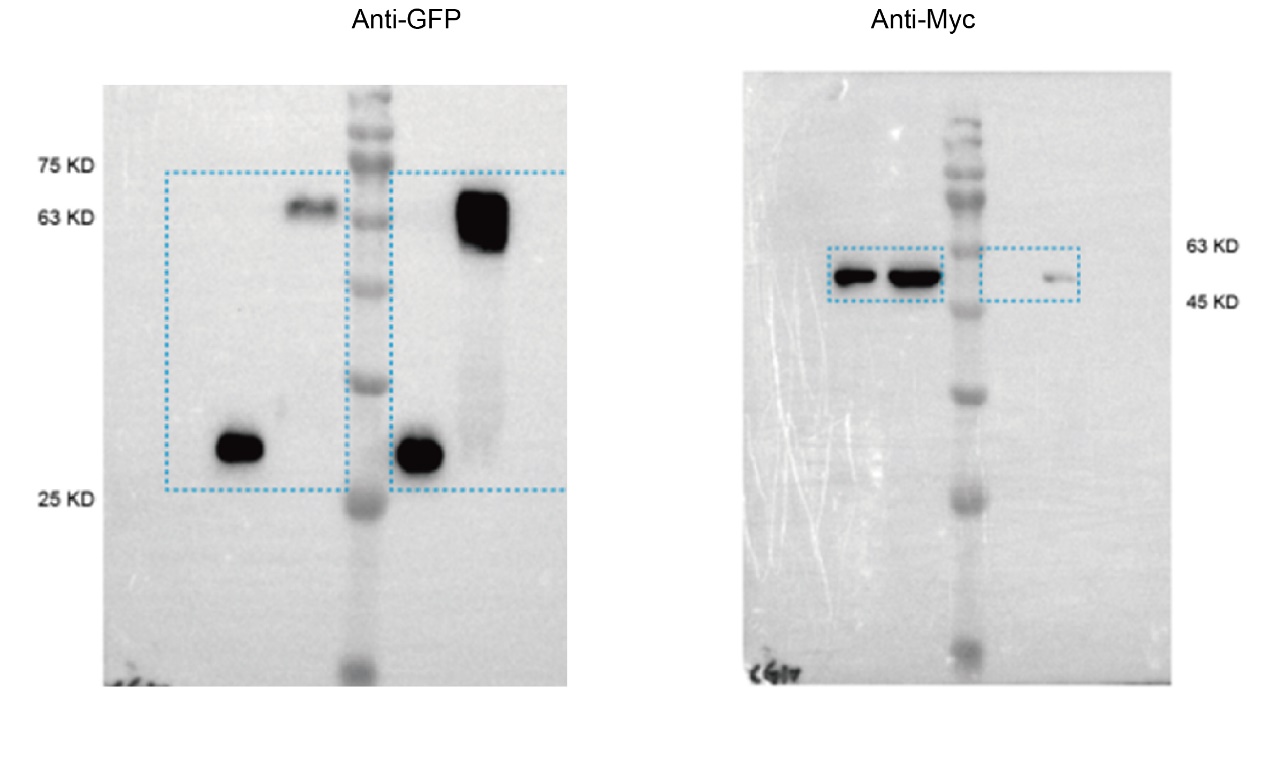


Fig. 6c Heterodimerization of TIRP1 and TIR1-TIR2_R detected by Co-IP in N. benthamiana leaves; GFP was used as a negative control and actin was used as a loading control


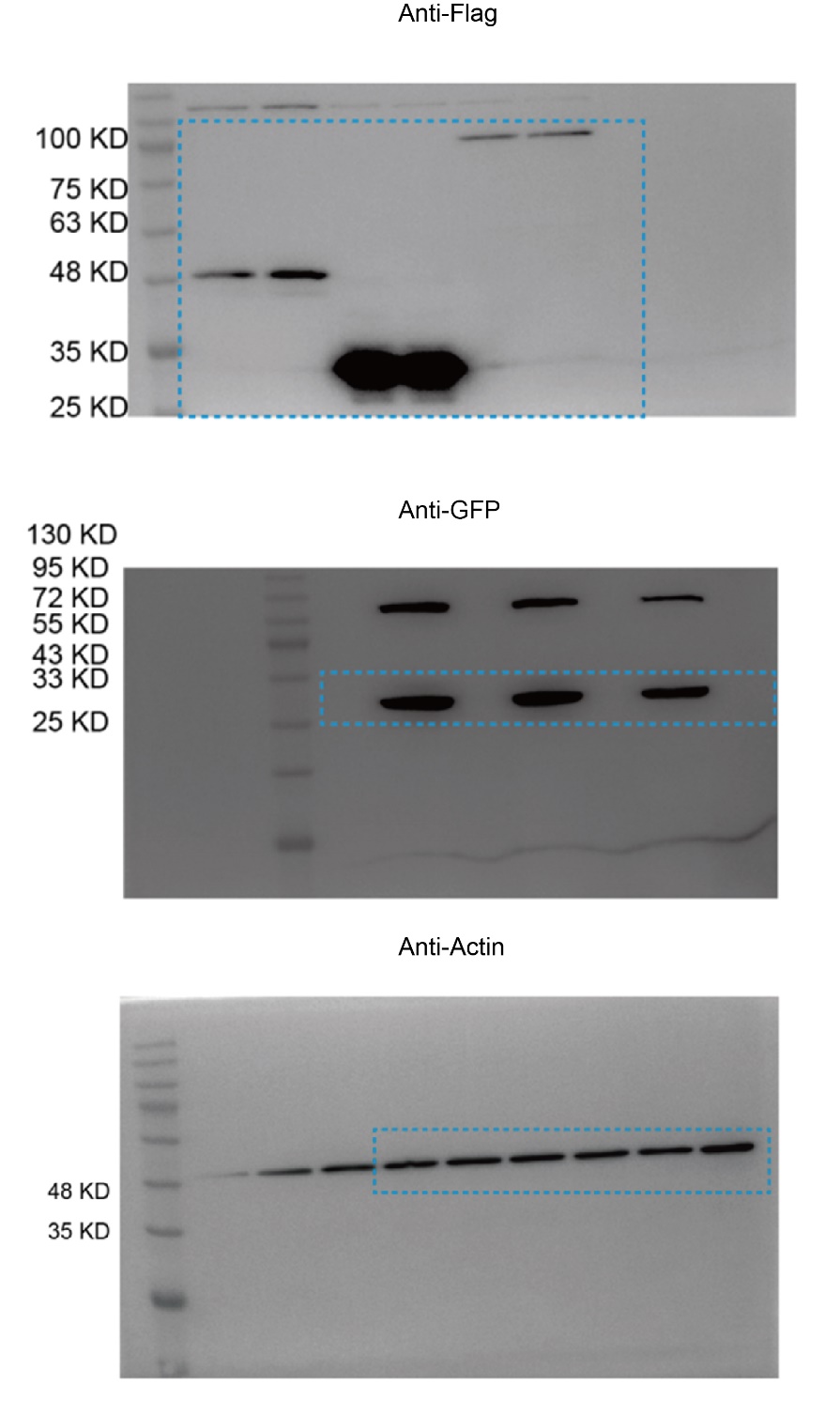


Fig. 6f Evaluation of protein expression using α-Flag (for TIR1-TIR2, RPP1_NdATIR, and RPS2) and α-GFP (for TIRP1); Immunoblotting of plant actin with α-actin was used as a loading control


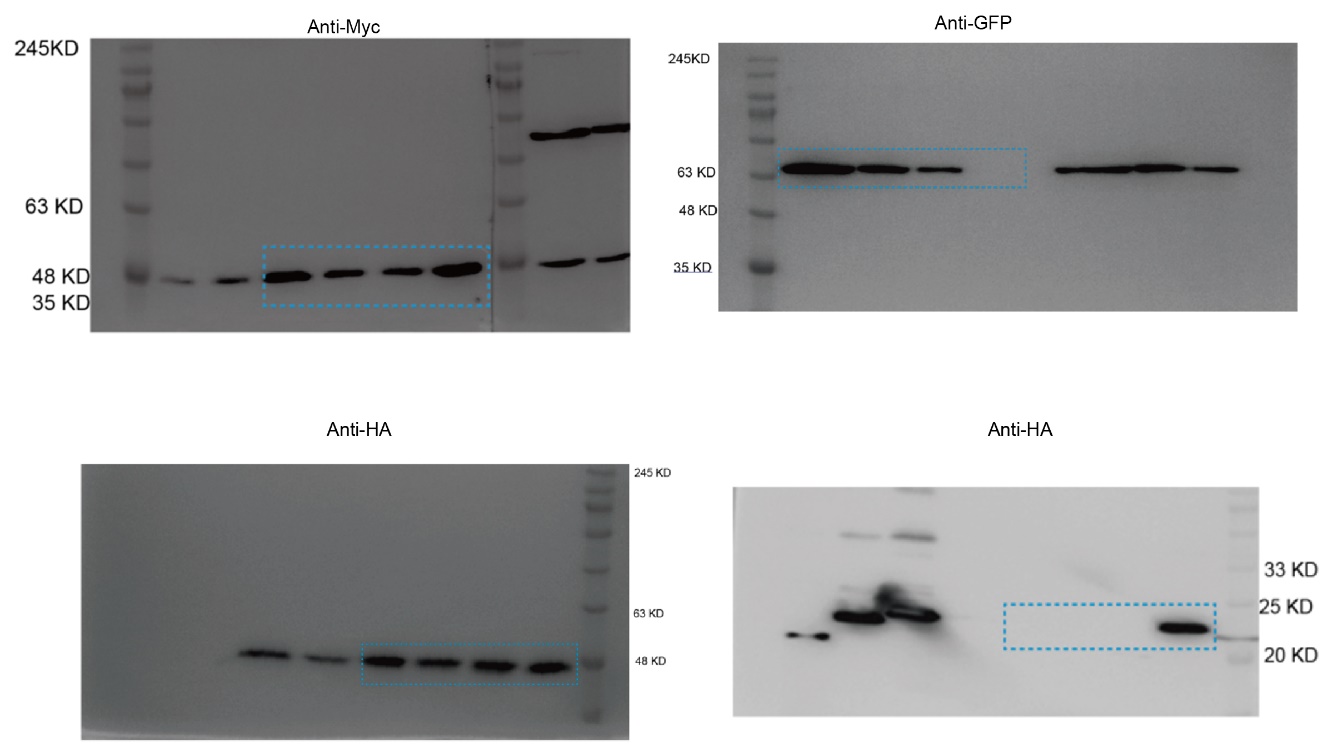


Fig. 6g-1 TIRP1 outcompetes TIR1-TIR2_R for binding to TIR1-TIR2_R in a concentration-dependent manner in *N. benthamiana* leaves, as determined by co-immunoprecipitation (Input Group).


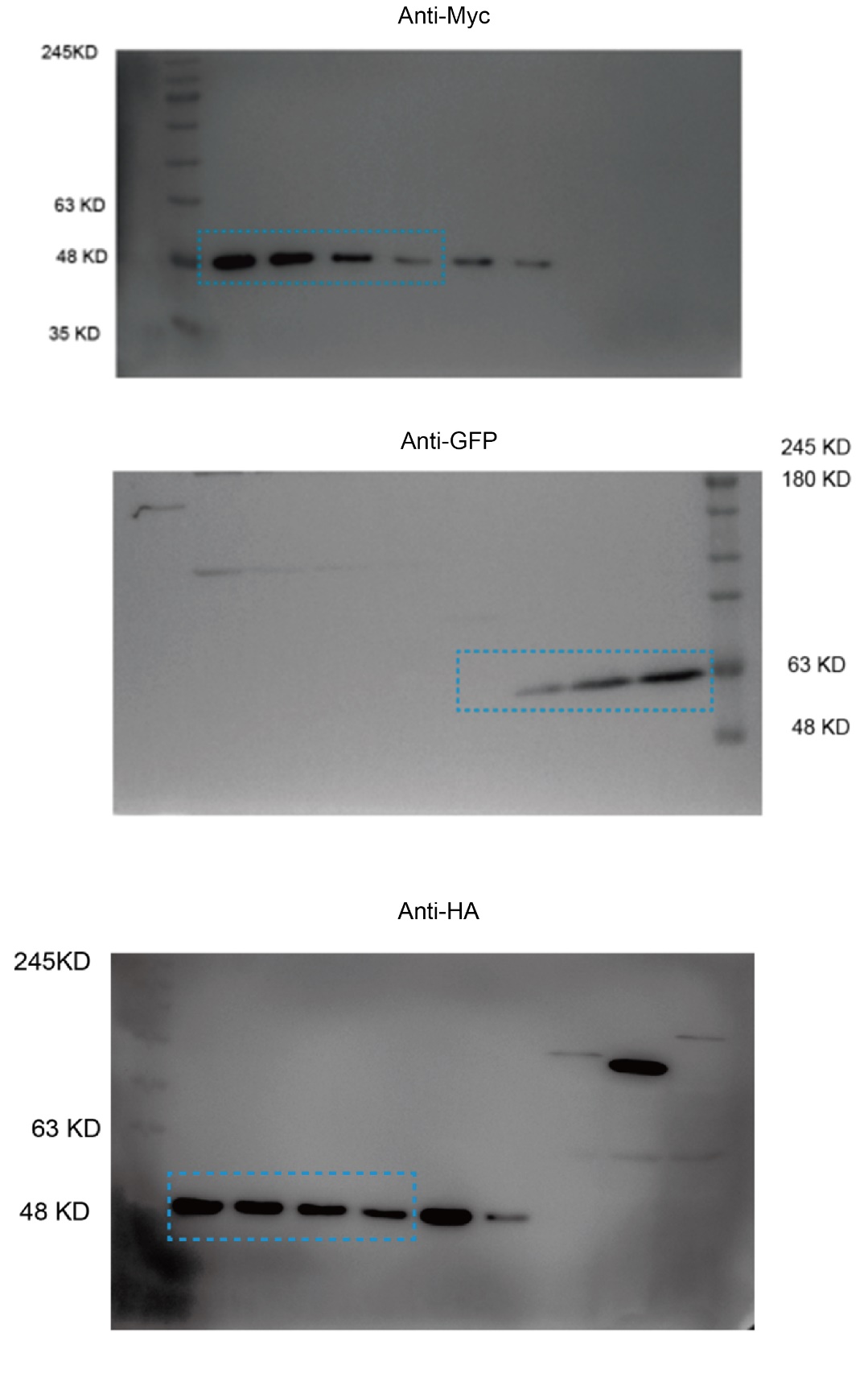


Fig. 6g-2 TIRP1 outcompetes TIR1-TIR2_R for binding to TIR1-TIR2_R in a concentration-dependent manner in *N. benthamiana* leaves, as determined by co-immunoprecipitation (IP Group).

­
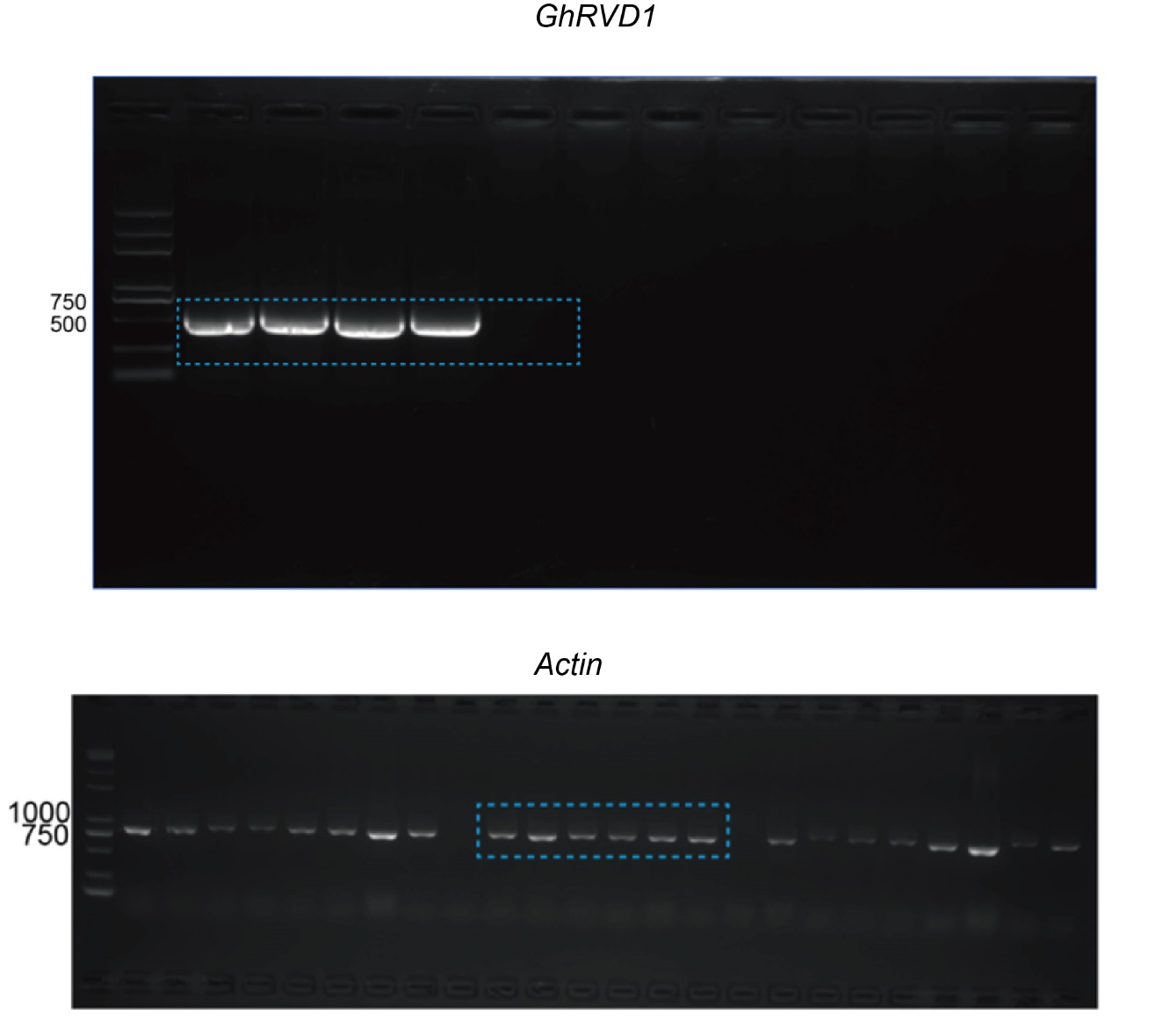


Fig. S5 Exogenous transcripts detected in both genotypes using semi-quantitative PCR.
