# Supplementary material for: Genome-wide association analysis reveals a novel pathway mediated by a dual-TIR domain protein for pathogen resistance in cotton
Source: Genome Biol. 2023 May 10;24:111. doi: 10.1186/s13059-023-02950-9 (PMC10170703; doi:10.1186/s13059-023-02950-9)
Supplement: Supplementary file 4 — Additional file 4. Review history. [file 13059_2023_2950_MOESM4_ESM.docx]

Review History

**First round of review**

**Reviewer 1**

**Were you able to assess all statistics in the manuscript, including the appropriateness of statistical tests used?**

No, I do not feel adequately qualified to assess the statistics.

**Comments to author:**

Verticillium wilt (VW) is a devasting disease causing huge economic losses for many crops. The authors identified VW resistance loci in G. hirsutum with GWAS, and interestingly found the loci was introgressed from a diploid species G. arboretum. Importantly, they further verified a new dual-TIR NLR gene GhRVD1 as the casual gene for the VW resistance. The functional analysis of GhRVD1 TIR dimerization and interaction with TIRP1 was well performed. I only have a couple of suggestions for the readers to consider.

Since the authors described "The triple hybrid (G. arboreum × G. thurberi × G. hirsutum) and Gossypium barbadense have been widely used in upland cotton breeding", why did the authors just focus on examination of introgressions from G. arboretum? How did the authors rule out the possibilities that introgressions were from the two other species?

In the GWAS analysis, which G. hirsutum genome reference was used? According to Fig. 1c, only a small percentage (5.8%) of G. hirsutum accessions harbor this introgressed genomic sequence (~1.2Mb).

For the phylogenetic tree shown in Fig. 1e, it is not clear whether the authors to use SNPs located in the putatively introgressed genomic region (highlighted in blue box in Fig. 1c). The tree is suggested to be shown with a radiation view with bootstrap values presented in some critical nodes. The colors of the branches are suggested to be consistent with the haplotypes color-coded in Fig. 3c. In addition, I would suggest to construct a gene tree based on the SNPs of the identified causal gene GhRVD1.

For Fig4b Fig4c: The protein level of K319T, K326N and K319T/K326N seems to be lower than that of proteins with other mutations. Is it possible the reason why proteins with those mutations showed weaker cell death intensity?

It is very confusing whether the authors use K326E or K326N. It is inconsistent in the main text and figures. Page10 Line 265: "but the strength of cell death was not further attenuated in K319T/K326E" & Page11 Line272: "Similarly, we found LUC activities in RS, K319T, K326E, and K319T/K326E……". As shown in Figure3a, the susceptible allele of TIR-2 on amino acid 326 was "E/Glu". It would be more reasonable to construct a K326E mutation instead of K326N. Please clarify this.

Minor:

Page4 Line109: "Similarly, AYDI and SHZDP represent DI in Anyang and Shihezi." "SHZDP" should be "SHZDI".

Page4 Line119: Are those "previously reported QTL regions" marked red in Fig 1a (QVW-A10, QF4-LS-d5, QDlnc-c17-1/Qfw-d3)? If so, then "SHZDI2" should be either "AYDP2" or "AYDI3". If not, please mark it on figure1a.

The -log(P) should be corrected as -log10(P) throughout the manuscript and in main and supplementary figures.

In the method section, I feel the methods related to bioinformatics were too brief to be one paragraph, and could be reorganized. Therefore, I would suggest the authors to merge the methods of 'SNP-based phylogenetic tree', 'Population differentiation (Fst) analysis', 'Collinearity analysis' into one paragraph as 'Evolutionary analyses'. In addition, the 'Identification of candidate genes by nucleotide polymorphism analysis' and 'Haplotype block analysis' paragraph could be combined into 'Genome-wide association study'.

For Fig. S6a, the aa ranges of R-TIR1 and R-TIR2 are different from the descriptions in the manuscript (Line: 240 - 242). In addition, the bootstrap values lower than 50 should not be shown. Latin names in the tree should be in italic.

For Fig. 4b, K319T/K226N should be corrected as K319T/K326N (or K319T/K326E?).

The format of the P value should be consistent throughout the manuscript, i.e., 'P' in italic.

For heatmaps showing the expression patterns (Fig. S2-4), please double check whether the legends are corrected (log10*(FPKM) or Z-score).

**Reviewer 2**

**Were you able to assess all statistics in the manuscript, including the appropriateness of statistical tests used?**

No, I do not feel adequately qualified to assess the statistics.

**Comments to author:**

The research identified one functional TNL protein with a dual-TIR domain that was not assessed before. The overexpression and knockdown of gene GhRVD1 with the TNL protein in cotton confirmed that GhRVD1 is one of the causal gene related the resistance to V. dahlia. The resistance response was mediated by rapid immune response, which was indued by the autoactivity and self-association of TIR1-TIR2. Then the authors found one negative regulator (GhTIRP1) that can inhibit the autoactivity and selfassociation of TIR1-TIR2 by competing for binding to it, further inhibiting acquired resistance to V. dahliae. In general, the research present some interesting findings to a broad audience of biologist, and majority of the conclusions are supported by the data. However, the authors need pay attention or explain the following several major concerns and minor concerns.

Major concerns

1. The single result "Compared with RR and SR, the intensity of cell death in SS and RS is significantly weaker" can not make the conclusion that "point mutations between TIR1-TIR2_R and TIR1-TIR2_S in the TIR2 (170-354) domain are responsible for the HR response's intensity", which easily gives the audience a hint that "point mutations between TIR1-TIR2_R and TIR1-TIR2_S in the TIR1 domain are not responsible for the HR response's intensity".

2. Please carry out the statistical analysis to compare the HR response between point mutations in TIR1 and TIR2, rather than only presenting one photo without repeats, such as Figs 4b, 4i, 5a, and so on.

3. Fig 4b described the phenomenon of cell death, why do the authors say that "It is difficult to directly observe the cell death phenotype in cotton leaves"? On the contrary, I do not think the fluorescence signal is the direct proof for cell death. Or I misunderstand something?

4. Where is the result as described in lines 367-370?

5. How did the author make the conclusion that the cell death phenotype induced by TIR1-TIR2 was more intense and took place earlier than done by RPP1_NdATIR and RPS2? We may only say that the cell death triggered by TIR1-TIR2 is independent to that by RPP1_NdATIR and RPS2 as showed in Fig.6e-f. In addition, I do not understand the relationship between line 394-394 and Sup Fig 7.

6. It is better to present the dosage effect of TIRP1 on self-association of TIR1-TIR2 in the competition model of TIRP1 and TIR1-TIR2 in Fig 7.

7. TIR1 and TIR2 are two domains in one gene, whether is it appropriate to call TIR1-TIR2 as heterodimer?

8. The format in References need to be improved, such as italic for Latin name.

9. It will be better if the authors present the significance of the finding in promoting the breeding or production of cotton.

Minor concerns

10. Line 79 "introgression efforts" rather than " introgressed efforts"?

11. Line 81 "across" rather than "cross"? "well-defined" should be "well-characterized".

12. Please precisely cite the supplementary figures, such as Fig S1b, 1c, ……, rather than just Fig S1, for the easy understanding.

13. Supplementary Fig 2 may be numbered as Supplementary Fig 4 according to its first citation in the text. If so, Supplementary Fig 3 & 4 should also be changed accordingly.

14. The points in Manhattan plot overlapped apparently between chromosomes. It's better to reduce the point size.

15. There are many language problems. For example, Line 131 "check" rather than "checked". Line 210-212, "and" should be added between clauses. Line 169 along or alone?

16. Line 149-151, 154-156, Line 157 "A total of 74 genes are located in ..." should be "A total of 74 genes locate in ..."

17. Line 164 "differentially" rather than "different".

18. I do not think that the results about artificial constructs have been described in detail so as to be understood easily in both main text (line 204-206) and legend of Sup Fig 5 (such as GhRVD1_R/S vs GhRVD1_S/R).

19. Line 220 "HR" is hypersensitive response, I guess. Please provide the full description in its first occurrence in the text.

20. Please label the statistical significance in all figures such as Fig 3g, Fig S5e-f.

21. Line 236 The last letter "t" is the misspelled "the"?

22. K319T/K326N was misspelled as K319T/K226N in Fig 4b?

23. When comparing the lesion size between RR and K192N, it seems that K192N enhanced the HR response. Please pay attention to this phenomenon.

24. Fig 4c indicated that the a-Actin is not uniform.

25. Please number the mutation positions of TIR2 in Sup Fig S5b.

26. Line 310 "G142R-G321R" is misspelled?

27. The result of Fig 5d suggest that the TIR1-TIR2 can form self-associations, but I do not think the result itself can tell us the self-association initiates downstream signaling pathways.

28. Line 338-339 It's better to clearly point out "the independent transient expression" for TIR1L-YFP and TIR2L-YFP so as to be understood easily, like "When TIR1L-YFP and TIR2L-YFP were transiently expressed alone respectively, there were no cell death phenotypes were induced".

29. What is the difference between TIR1 and TIR1L?

30. It is better to carry out the immunoblotting in the same gel for Fig 5f.

31. Please provide the standard error bar and carry out the statistical significance analysis for result of Fig 6h.

**Authors Response**

**Point-by-point responses to the reviewers’ comments:**

Reviewer #1:

Verticillium wilt (VW) is a devasting disease causing huge economic losses for many crops. The authors identified VW resistance loci in G. hirsutum with GWAS, and interestingly found the loci was introgressed from a diploid species G. arboretum. Importantly, they further verified a new dual-TIR NLR gene GhRVD1 as the casual gene for the VW resistance. The functional analysis of GhRVD1 TIR dimerization and interaction with TIRP1 was well performed. I only have a couple of suggestions for the readers to consider.

(1) Since the authors described "The triple hybrid (G. arboreum × G. thurberi × G. hirsutum) and Gossypium barbadense have been widely used in upland cotton breeding", why did the authors just focus on examination of introgressions from G. arboretum? How did the authors rule out the possibilities that introgressions were from the two other species?

*Response: Thanks very much for the positive appraisal and the helpful guidance about how to improve our study. Addressing this first comment, we have now clarified this issue in the revised text. We used two methods to assess introgressions. On the one hand, we used a recently reported introgression index method to analyzed whether a given introgression was from G. thurberi, G. barbadense, or G. arboreum (He et al., 2021, citation 26 in the main text). First, given the knowledge that G. thurberi is a diploid D-genome cotton, we can safely assume that any introgressions from it will be vastly more likely into the upland cotton Dt subgenome (i.e., than the At subgenome). Given that the introgression-of-interest in our study is located on chromosome A10 of the At subgenome, we made the assumption that G. thurberi was exceedingly unlikely to be the donor for A10 introgressions, thus narrowing the field of donors to G. arboreum and G. barbadense. Using the introgression index method from the Nature Genetics study, we separately analyzed the introgressed fragments from G. arboreum and from G. barbadense. Fortunately, this analysis indicated that that SHZDI1 shared overlap with a fragment from G. aboreum and shared no overlap with G. barbadense (See Additional file 2: Table S8), findings supporting that the introgressions are from G. arboreum. On the other hand, we also used a SNP-based phylogenetic tree method using FastTree with default parameters to further characterize these introgressions on A10 based on 7062 SNPs; our tree comprised upland cotton, G. barbadense, and G. arboreum. As you can see in Fig. S2a, the introgressed lines clustered together with G. arboreum, again supporting that the introgressed fragments are from G. arboreum. We have now added clarification and details about how we identified introgressions in both the main text and method sections of the revised manuscript (See Additional file 1: Fig. S2a and Page 6, lines 137-148).*

(2) In the GWAS analysis, which G. hirsutum genome reference was used? According to Fig. 1c, only a small percentage (5.8%) of G. hirsutum accessions harbor this introgressed genomic sequence (~1.2Mb)

*Response: Thanks very much for focusing our attention here. We have now clarified this point in both the main text and method sections of the revised manuscript (See page 5, lines 115-116; page 23, lines 612-613). Briefly, we used the latest high-quality tetraploid cotton genome (G. hirsutum ‘Texas Marker 1’) as the reference genome (Yang et al., 2019, Nature Communications). We also need to correct a mistake: a total of 36 accessions carried these introgressions, accounting for 8.5% in the GWAS panel. We wrote 5.8% in error (See Page 7, line 168). Note that the introgressed fragments were scanned “one sample by one sample” in our study, and minor allele frequency did not affect the introgression identification (See Page 6, lines 142-143; Page 24, line 644; Additional file 2: Table 8).*

(3) For the phylogenetic tree shown in Fig. 1e, it is not clear whether the authors to use SNPs located in the putatively introgressed genomic region (highlighted in blue box in Fig. 1c). The tree is suggested to be shown with a radiation view with bootstrap values presented in some critical nodes. The colors of the branches are suggested to be consistent with the haplotypes color-coded in Fig. 3c. In addition, I would suggest to construct a gene tree based on the SNPs of the identified causal gene GhRVD1.

*Response: Thanks very much for this helpful guidance. Indeed, the SNPs located in the putatively introgressed genomic region (112.70Mb-113.75Mb) were used to construct the SNP-based phylogenetic tree. We have revised the related statement in the main text, which now reads as:*

*“We next constructed a SNP-based phylogenetic tree using the SNPs from the putatively introgressed genomic region (highlighted in the blue box in Fig. 1c). The phylogenetic tree indicated that accessions carrying Hap. B were clustered with accessions carrying Hap. 1, further supporting that Hap. B is introgressed from G. arboreum” (See Page 6, lines 155-157).*

*We also now present the phylogenetic tree in a radiation view with bootstrap values presented in various critical nodes (See Fig. 1e).*

*Following your comment, we have also now constructed a gene tree based on the SNPs of the identified causal gene GhRVD1.This gene tree also supports that the introgressions are from G. arboreum, highly similar with result by the SNP-tree based on SNPs located in the putatively introgressed genomic region (See Additional file 1: Fig. S6i; Page 24, lines 654-656).*

(4) For Fig4b Fig4c: The protein level of K319T, K326N and K319T/K326N seems to be lower than that of proteins with other mutations. Is it possible the reason why proteins with those mutations showed weaker cell death intensity?

*Response: Thanks very much for bringing this issue to our attention (which we didn’t notice previously). We have now repeated this experiment. The new data demonstrate that the levels of Actin, TIR1-TIR2, and the TIR1-TIR2 variants are uniform. (See updated Fig. 4c). Thanks again for bringing this potential problem to our notice!*

(5) It is very confusing whether the authors use K326E or K326N. It is inconsistent in the main text and figures. Page10 Line 265: "but the strength of cell death was not further attenuated in K319T/K326E" & Page11 Line272: "Similarly, we found LUC activities in RS, K319T, K326E, and K319T/K326E……". As shown in Figure3a, the susceptible allele of TIR-2 on amino acid 326 was "E/Glu". It would be more reasonable to construct a K326E mutation instead of K326N. Please clarify this.

*Response: This was a typo. We intended to write K326E. We have corrected this throughout the main text and figures. (See Fig. 4a-g; Page 12, line 313).*

Minor

(1)Page4 Line109: "Similarly, AYDI and SHZDP represent DI in Anyang and Shihezi." "SHZDP" should be "SHZDI".

*Response: Thanks! We have corrected it (See Page 5, line 111).*

(2) Page4 Line119: Are those "previously reported QTL regions" marked red in Fig 1a (QVW-A10, QF4-LS-d5, QDlnc-c17-1/Qfw-d3)? If so, then "SHZDI2" should be either "AYDP2" or "AYDI3". If not, please mark it on figure1a.

*Response: Thanks! We have corrected it (See Page 5, line 123).*

(3) The -log(P) should be corrected as -log10(P) throughout the manuscript and in main and supplementary figures.

*Response: Thanks! Done (See Fig. 1a; Fig. 2a; Page 23, line 619; Additional file 1: Fig. S2b and S3a).*

(4) In the method section, I feel the methods related to bioinformatics were too brief to be one paragraph, and could be reorganized. Therefore, I would suggest the authors to merge the methods of 'SNP-based phylogenetic tree', 'Population differentiation (Fst) analysis', 'Collinearity analysis' into one paragraph as 'Evolutionary analyses'. In addition, the 'Identification of candidate genes by nucleotide polymorphism analysis' and 'Haplotype block analysis' paragraph could be combined into 'Genome-wide association study'.

*Response: Thanks very much for your suggestion. We have followed the suggestions for revisions (See Pages 23-25, lines 610-662).*

(5) For Fig. S6a, the aa ranges of R-TIR1 and R-TIR2 are different from the descriptions in the manuscript (Line: 240 - 242). In addition, the bootstrap values lower than 50 should not be shown. Latin names in the tree should be in italic.

*Response: This was a typo in Fig. S6a. Animo acid of TIR1 (1-169) and TIR2 (170-354) and other homologous sequences were used to construct a Phylogenetic tree. We revised and updated the Fig. S6a. Following your comment, we have also now updated the presence of bootstrap values (See Additional file 1: Fig. S6a). Thanks very much.*

(6) For Fig. 4b, K319T/K226N should be corrected as K319T/K326N (or K319T/K326E?).

*Response: Thanks! We corrected K319T/K326N as K319T/K326E in the main text and figures (See Fig. 4b).*

(7) The format of the P value should be consistent throughout the manuscript, i.e., 'P' in italic.

*Response: Done! (See Page 5, line 117; Page 23, line 618; Page 28, line 764).*

(8) For heatmaps showing the expression patterns (Fig. S2-4), please double check whether the legends are corrected (log10*(FPKM) or Z-score)

*Response: Thanks very much for your suggestion. All the heatmaps in Fig. S2-4 were normalized by Z-score. We have ensured that this info is correct in the revised figures (See Additional file 1: Fig. S2c-f¬, S3b-f and S4a).*

*Again, we would like to thank Reviewer #1 for constructive comments and guidance.*

Reviewer #2:

The research identified one functional TNL protein with a dual-TIR domain that was not assessed before. The overexpression and knockdown of gene GhRVD1 with the TNL protein in cotton confirmed that GhRVD1 is one of the causal gene related the resistance to V. dahlia. The resistance response was mediated by rapid immune response, which was indued by the autoactivity and self-association of TIR1-TIR2. Then the authors found one negative regulator (GhTIRP1) that can inhibit the autoactivity and selfassociation of TIR1-TIR2 by competing for binding to it, further inhibiting acquired resistance to V. dahliae. In general, the research present some interesting findings to a broad audience of biologist, and majority of the conclusions are supported by the data. However, the authors need pay attention or explain the following several major concerns and minor concerns.

Major concerns

1.The single result "Compared with RR and SR, the intensity of cell death in SS and RS is significantly weaker" can not make the conclusion that "point mutations between TIR1-TIR2_R and TIR1-TIR2_S in the TIR2 (170-354) domain are responsible for the HR response's intensity", which easily gives the audience a hint that "point mutations between TIR1-TIR2_R and TIR1-TIR2_S in the TIR1 domain are not responsible for the HR response's intensity".

*Response: We understand the problem with our unclear descriptions in the originally submitted manuscript. We have supplemented the trypan blue dyeing and luciferase activity to illustrate the cell death intensity and have now rephrased them as follows:*

*“We found the intensity of cell death caused by TIR1-TIR2_S (SS) is significantly weaker than that caused by TIR1-TIR2_R (RR). To confirm whether a single TIR or both TIRs were responsible for the difference in cell death intensity, we artificially created two mutants: TIR1-S-TIR2-R (SR) and TIR1-R-TIR2-S (RS) in TIR1-TIR2_R background. When TIR1-R was mutated to TIR1-S by mutating 5 nucleotides, the cell death intensity of the first mutant SR has no difference with that of RR. However, when TIR2-R was mutated to TIR2-S by mutating 5 nucleotides, the cell death intensity of the second mutant RS significantly decreased (Fig. 4a-b and Additional file 1: Fig. S7a). Trypan blue detection of death cells also showed that RR and SR induced higher intensity of cell death compared with both SS and RS (Additional file 1: Fig. S8). The firefly Luciferase (LUC) reporter gene system has been a powerful tool to monitor the cell viability through quantifying the fluorescence activity in many studies [47-50]. Here, we also co-expressed of the candidate proteins and LUC to perform the cell-death assay. Both the fluorescence signaling and luciferase activity again confirm that RR and SR showed stronger intensity of cell death than both SS and RS (Fig. 4d-e and Additional file 1: Fig. S6h). Viewed together, our results suggest that the HR response's intensity enhanced in a manner depended on the mutations between the TIR2-R and TIR2-S.”*

*Thanks very much! (See Fig. 4a-b; Fig. 4d-e; Additional file 1: Fig. S7a and S8; Page 12, lines 297-312)*

2.Please carry out the statistical analysis to compare the HR response between point mutations in TIR1 and TIR2, rather than only presenting one photo without repeats, such as Figs 4b, 4i, 5a, and so on.

*Response: Thanks very much for your suggestion. We have now completed inferential statistical analysis of our 6 biological replicates for each sample and added this content to the revised manuscript (See Additional file 1: Fig. S7a-e; Page 28, lines 756-757).*

3. Fig 4b described the phenomenon of cell death, why do the authors say that "It is difficult to directly observe the cell death phenotype in cotton leaves"? On the contrary, I do not think the fluorescence signal is the direct proof for cell death. Or I misunderstand something?

*Response: We need to clarify that in Fig. 4b, the experiments were performed on tobacco leaves (i.e., not on cotton leaves). Note that we have now removed the claim “It is difficult to directly observe the cell death phenotype in cotton leaves” from the text.*

*For some additional context, the LUC report gene system has been frequently employed to monitor cell death (using LUC activity as an indicator of cell viability) (see for example Chen et al., 2022; Deng et al., 2022; Wang et al., 2021; Yoshida et al., 2009). Cell death results in a low fluorescence level in N. benthamiana leaves, so the intensity of fluorescence is negatively correlated with the cell death intensity. To better understand this method, we added some relevant references in the main context (See Page 12 line 307). We have now used luciferase assay system (Promega, USA) to monitor the cell viability which is another method to detect activity of Luciferase and get a similar conclusion with those based on fluorescence signal (See Additional file 1: Fig. S6h and page 12 lines 307-310).*

*References:*

*1. Chen G, Zhang B, Ding J, Wang H, Deng C, Wang J, et al. Cloning southern corn rust resistant gene RppK and its cognate gene AvrRppK from Puccinia polysora. Nat Commun. 2022;13(1):4392.*

*2. Deng C, Leonard A, Cahill J, Lv M, Li Y, Thatcher S, et al. The RppC-AvrRppC NLR-effector interaction mediates the resistance to southern corn rust in maize. Mol Plant. 2022;15(5):904-912.*

*3. Wang Z, Huang J, Nie L, Hu Y, Zhang N, Guo Q, et al. Molecular and functional analysis of a brown planthopper resistance protein with two nucleotide-binding site domains. J Exp Bot. 2021;72(7):2657-2671.*

*4. Yoshida K, Saitoh H, Fujisawa S, Kanzaki H, Matsumura H, Yoshida K, et al. Association genetics reveals three novel avirulence genes from the rice blast fungal pathogen Magnaporthe oryzae. Plant Cell. 2009;21(5):1573-1591.*

4.Where is the result as described in lines 367-370?

*Response: The context in lines 367-370 was related to Fig. S6d-f, we have added the citation of this figure in the main context (See Page 17, line 439).*

5.How did the author make the conclusion that the cell death phenotype induced by TIR1-TIR2 was more intense and took place earlier than done by RPP1_NdATIR and RPS2? We may only say that the cell death triggered by TIR1-TIR2 is independent to that by RPP1_NdATIR and RPS2 as showed in Fig.6e-f. In addition, I do not understand the relationship between line 394-394 and Sup Fig 7.

*Response: Thank you for these helpful clarifications and suggested solutions to improve our reasoning and interpretation. Following your guidance, we have revised these statements as follows:*

*“NLR immune receptors have been classified into TNL and coiled-coil (CC) NLRs (CNL). The TIR domain of RPP1_NdA, TNL immune receptors from A. thaliana, which can trigger cell death when ectopically expressed in Nicotiana species [53]. Cell death induced by AtRPS2, which encodes a CNL, requires different signaling components to mount the ETI response than TNLs [50,53,57]. To test whether TIRP1 also inhibits the typical NLR immune receptors, the RPP1_NdATIR and AtRPS2 were taken to co-inoculate with different concentrations of TIRP1 in tobacco leaves. Interestingly, increasing concentrations of TIRP1 was not effective in reducing cell death triggered by RPP1_NdATIR and RPS2 (Fig.6e-f and Additional file 1: Fig. S7e), which was further measured by Luciferase reporter (Additional file 1: Fig. S9c), indicating that inhibition of NLR autoactivity by TIRP1 is not universal in plants. Collectively, our results suggest that TIRP1 affects the autoactivity of TIR1-TIR2 when present together.”*

*Thanks very much for your suggestion (See Page 18, lines 469-480).*

6.It is better to present the dosage effect of TIRP1 on self-association of TIR1-TIR2 in the competition model of TIRP1 and TIR1-TIR2 in Fig 7.

*Response: Thanks very much for your suggestion. Done! (See Fig. 7).*

7.TIR1 and TIR2 are two domains in one gene, whether is it appropriate to call TIR1-TIR2 as heterodimer?

*Response: Okay, the “heterodimer” has been replaced by “physical interaction” in the manuscript. Thanks very much for your suggestion (See Page 2, line 29; Page 4, line 96; Page 15, line 390; Page 16, line 423; Page 16, line 428; Page 21, line 562; Page 22, line 588; Page 43, line 1162).*

8.The format in References need to be improved, such as italic for Latin name.

*Response: We have now carefully examined the formatting of the references. Thanks very much for your suggestion (See the reference section).*

9.It will be better if the authors present the significance of the finding in promoting the breeding or production of cotton.

*Response: Thanks very much for this guidance. We have now added content to present the significance of our study in the abstract, introduction, and discussion sections of our revised manuscript (See Page 2, lines 37-38; Page 5, lines 101-102; Pages 19-20, lines 510-522; Pages 20-21, lines 543-548; Page 22, lines 579-581). For example, in the discussion section, we have added a paragraph that reads:*

*“Note that in a core panel of 419 accessions, only 36 accessions carried these introgressed fragments, indicating that this valuable resource has not been widely used in cotton breeding. We know that upland cotton was domesticated from Mexico, prior to further domestication and improvement in the United States, this history is evident in the narrow genetic background and relatively low genetic diversity of this crop species [58]. Distant hybridization breeding between diploid cotton and tetraploid cotton is understood as an effective means to increase the genetic diversity of upland cotton. Our successful identification of SHZDI1/SHZDP2/AYDP1 offers an example for how an introgressed fragments has contributed to major improvements in VW resistance in upland cotton. The locus SHZDI1/SHZDP2/AYDP1 could be developed into a molecular marker that would allow breeders to directly select this locus when seeking to improve the VW resistance trait in elite lines”.*

Minor concerns

10.Line 79 "introgression efforts" rather than " introgressed efforts"?

*Response: Thanks very much for your suggestion. We have revised it (See Page 4, line 79).*

11.Line 81 "across" rather than "cross"? "well-defined" should be "well-characterized".

*Response: We have corrected these, thanks (See Page 4, lines 82-83).*

12.Please precisely cite the supplementary figures, such as Fig S1b, 1c, ……, rather than just Fig S1, for the easy understanding.

*Response: Okay, we thoroughly checked all the figure item indications in the main context and have supplemented the figure panel information for the supplementary figures. Thanks!*

13. Supplementary Fig 2 may be numbered as Supplementary Fig 4 according to its first citation in the text. If so, Supplementary Fig 3 & 4 should also be changed accordingly.

*Response: Done! Thanks!*

14.The points in Manhattan plot overlapped apparently between chromosomes. It's better to reduce the point size.

*Response: Done! Thanks! (See Fig. 1a; Additional file 1: Fig. S2b and S3a).*

15.There are many language problems. For example, Line 131 "check" rather than "checked". Line 210-212, "and" should be added between clauses. Line 169 along or alone?

*Response: Thanks very much for your suggestion. We have invited a native English expert with a PhD in plant biology to help us polish our language (See Page 6, line 148; Page 10, line 245; See Page 8, line 190).*

16.Line 149-151, 154-156, Line 157 "A total of 74 genes are located in ..." should be "A total of 74 genes locate in ..."

*Response: We have corrected it, thanks (See Page 7, line 177; Page; Page 11, line 275; Page 11, line 276).*

17.Line 164 "differentially" rather than "different".

*Response: Done!　Thanks! (See Page 8, line 185)*

18.I do not think that the results about artificial constructs have been described in detail so as to be understood easily in both main text (line 204-206) and legend of Sup Fig 5 (such as GhRVD1_R/S vs GhRVD1_S/R).

*Response: Thanks very much for this guidance. We have supplemented the details in both the main text and legends. (See Page 9, line 232-240)*

19.Line 220 "HR" is hypersensitive response, I guess. Please provide the full description in its first occurrence in the text.

*Response: Done! (See Page 10, line 252)*

20.Please label the statistical significance in all figures such as Fig 3g, Fig S5e-f.

*Response: Thanks very much for this guidance. We have added the statistical support (See Fig. 3g; Additional file 1: Fig. S5e-f)*

21.Line 236 The last letter "t" is the misspelled "the"?

*Response: We removed letter “t”, thanks! (See Page 11, line 271)*

22.K319T/K326N was misspelled as K319T/K226N in Fig 4b?

*Response: Yes! Thanks very much for bringing this error to our attention. K326N should be K326E. We have corrected it in the revised manuscript. (See Fig. 4b)*

23. When comparing the lesion size between RR and K192N, it seems that K192N enhanced the HR response. Please pay attention to this phenomenon.

*Response: Thanks very much for bringing this to our attention. We have now considered this in greater detail. We have 6 biological replicates for each experiment, and we found no significant differences between K192N and RR in an analysis of necrosis percentages in the infiltration areas (See Additional file 1: Fig. S7a). In addition, the LUC fluorescence intensity data again supports no difference in the extent of the HR between RR and K192N (See Fig. 4e; Page 11, lines 316-317).*

24. Fig 4c indicated that the a-Actin is not uniform.

*Response: Thanks very much for bringing this issue to our attention (which we didn’t notice previously). We have now repeated this experiment. The new data demonstrate that the levels of Actin, TIR1-TIR2, and the TIR1-TIR2 variants are uniform. (See updated Fig. 4c). Thanks again for bringing this potential problem to our notice!*

25. Please number the mutation positions of TIR2 in Sup Fig S5b.

*Response: Thanks very much for your guidance. Done! (See Additional file 1: Fig. S5b)*

26. Line 310 "G142R-G321R" is misspelled?

*Response: This was a typo in the main text. G142R-G321R should have been G142R/G312R; we have now corrected it. Thanks very much (See Page 14, line 369).*

27. The result of Fig 5d suggest that the TIR1-TIR2 can form self-associations, but I do not think the result itself can tell us the self-association initiates downstream signaling pathways.

*Response: We understand the problem here, and removed the interpretation about signaling. The revised the statement reads as follows:*

*“In Co-IP assays, we immunoprecipitated TIR1-TIR2-HA proteins with an anti-HA antibody and performed immunoblotting with an anti-Myc antibody. The TIR1-TIR2-Myc protein was detected only in the precipitate from the leaves that co-expressed both TIR1-TIR2-HA and TIR1-TIR2-Myc, suggesting that TIR1-TIR2 can form self-associations” (See Page 15, line 379).*

28. Line 338-339 It's better to clearly point out "the independent transient expression" for TIR1L-YFP and TIR2L-YFP so as to be understood easily, like "When TIR1L-YFP and TIR2L-YFP were transiently expressed alone respectively, there were no cell death phenotypes were induced".

*Response: Done! Thanks for your comments (See Page 15, line 403).*

29. What is the difference between TIR1 and TIR1L?

*Response: The TIR1 domain was predicted in silico. To ensure the integrity of TIR1 domain, we extended 20 amino acids on the right side of TIR1 and named it as TIR1L. We supplemented the description in the main text (See page 15, line 396-400).*

30. It is better to carry out the immunoblotting in the same gel for Fig 5f.

*Response: Following your instruction, we have now completed a new immunoblot gel with all the relevant samples in the same gel in Fig 5f.*

31. Please provide the standard error bar and carry out the statistical significance analysis for result of Fig 6h.

*Response: Thanks for this guidance. Done! (See Fig. 6h)*

*We would like to thank Reviewer #2 for the clarifying guidance and constructive comments.*

**Second round of review**

**Reviewer 1**

The authors have addressed all my questions satisfactorily. Nice work!

**Reviewer 2**

In the revision, the quality of the manuscript has been further improved. Most of my concerns have been addressed in a satisfying way. Thank the authors for carefully answering my comments. Here, I just have two suggestions.

The reliability of 3D structure should be enhanced. The article did not resolve the 3D structure of GhRVD1 by experiment, but mainly utilize one kind of homologous modeling for GhRVD1 protein structure dissection (with Phypre2). It may not be convincing to use only one homologous model. To ensure the reliability of the identified structure, it’s better to provide an alternative prediction method as an auxiliary evidence.

The description of the model proposed in Figure 7 is too simple. After all, this is the summary of the main conclusions drawn from the whole story. In addition, it’s better to clearly indicate the difference between normal condition and V.dahliae stress for the pathway from HSR203J/HIN1 to SA to NPR1/PR1, for example, using arrows with different thickness.

**Authors Response**

**Point-by-point responses to the reviewers’ comments:**

Reviewer #1:

The authors have addressed all my questions satisfactorily. Nice work!

*Response: Thanks very much for your supporting.*

Reviewer #2:

In the revision, the quality of the manuscript has been further improved. Most of my concerns have been addressed in a satisfying way. Thank the authors for carefully answering my comments. Here, I just have two suggestions.

The reliability of 3D structure should be enhanced. The article did not resolve the 3D structure of GhRVD1 by experiment, but mainly utilize one kind of homologous modeling for GhRVD1 protein structure dissection (with Phypre2). It may not be convincing to use only one homologous model. To ensure the reliability of the identified structure, it’s better to provide an alternative prediction method as an auxiliary evidence.

*Response: Thanks very much for your suggestion. we discussed the limitations of our prediction of the 3D structure of GhRVD1 using Phyre2 in the Discussion section. Now it reads as follows: “Phyre2 uses advanced remote homology detection methods to build 3D models [64], which are considered a suite of tools available in many studies [65]. We used Phyre2 at 90% accuracy to build the model for TIR1-TIR2, which was based on a template with the best-aligned 3D structures. Actually, four out of the five-stranded parallel β-sheet and all five α-helical regions, which are well-characterized in known TIR structures, were also well predicted in TIR1 and TIR2 of GhRVD1. However, without experimental support, there is potentially some uncertainty regarding the accuracy of the predicted model. For example, the absence of the βB-sheet in TIR1 and TIR2 needs further validation through experimental techniques such as X-ray crystallography or cryo-electron microscopy”.*

The description of the model proposed in Figure 7 is too simple. After all, this is the summary of the main conclusions drawn from the whole story. In addition, it’s better to clearly indicate the difference between normal condition and V.dahliae stress for the pathway from HSR203J/HIN1 to SA to NPR1/PR1, for example, using arrows with different thickness.

*Response: Thanks very much for your suggestion. We have updated the figure legend for Figure 7 by providing a more detailed working model. The revised version now reads as follows: “Under normal growth conditions, the constitutive expression of TIRP1 mediate accumulation of sufficient protein to competitively bind to TIR1-TIR2, thus inhibiting their self-association, and subsequent immune response mediated by GhRVD1. The downregulation of TIRP1 expression after V. dahliae invasion releases the TIRP1 from TIR1-TIR2 domain of GhRVD1 and indirectly leads to increased TIR1-TIR2 self-association. Furthermore, increasing dimer of TIR1-TIR2 contributes to activate the transcription of hypersensitive response-related gene HSR203J/HIN1 and systemic acquired resistance-related gene NPR1/PR1, thus enhancing cotton resistance against V. dahliae. The width of the arrows indicates the strength of the downstream signaling. Blue circles represent molecules associated with V. dahliae infection”.*

*Additionally, We also now present the difference between normal condition and V.dahliae stress for the pathway using arrows with different thickness. Thanks very much for bringing this to our attention.*
